# User-centered design and formative evaluation of a gait dashboard for multidimensional walking assessment in multiple sclerosis

**DOI:** 10.3389/fdgth.2026.1865736

**Published:** 2026-09-08

**Authors:** Brita Sedlmayr, Katrin Trentzsch, Maria Zerlik, Sophia Grummt, Heidi Stölzer-Hutsch, Maximilian Rechenberg, Maximilian Hartmann, Caroline Glathe, Katharina Schuler, Tjalf Ziemssen

**Affiliations:** 1 Institute for Medical Informatics and Biometry, Faculty of Medicine, University Hospital Carl Gustav Carus, TUD Dresden University of Technology, Dresden, Germany; 2 Center of Clinical Neuroscience, Department of Neurology, Faculty of Medicine and University Hospital Carl Gustav Carus Dresden, TUD Dresden University of Technology, Dresden, Germany; 3 Centre for Tactile Internet with Human-in-the-Loop (CeTI), TUD Dresden University of Technology, Dresden, Germany

**Keywords:** dashboard, data visualization, digital health, gait analysis, multiple sclerosis, user-centered design

## Abstract

Digital gait analysis using wearable sensors and instrumented walkways is increasingly implemented in routine care for people with multiple sclerosis. However, multidimensional walking assessments generate longitudinal and heterogeneous outputs that are often presented in fragmented, hard-to-interpret reports, limiting their usefulness for clinical communication and shared decision-making. This study aimed to develop a patient-centered dashboard that integrates multidimensional gait data into a coherent and intuitive overview, to apply a human-centered design process aligned with routine clinical workflows, and to conduct a formative concept evaluation of the dashboard regarding comprehensibility, perceived usability, and perceived usefulness from the perspectives of medical professionals and patients. User requirements were identified through contextual interviews, workflow analysis, persona development, a stakeholder workshop, and a patient survey. Two mid-fidelity dashboard prototypes were iteratively developed and evaluated. The first prototype was assessed by medical professionals (*N* = 4) using a workshop-based walkthrough, while the revised version was evaluated by patients (*N* = 24) during clinic visits using a questionnaire following a narrated video walkthrough. Medical professionals rated the information structure, domain-based organization, and longitudinal visualizations positively and provided suggestions for improvement. In the patient evaluation, the revised prototype received high ratings across all assessed dimensions, and most participants (87.5%) preferred it over the previously used visualization, reporting improved understanding of their gait results and health status. However, differences in presentation format (narrated video vs. recall) may have influenced the comparison. Overall, the results indicate that a workflow-anchored, human-centered design approach can yield patient-centered visualizations that are understandable, well accepted, and clinically meaningful, with potential to improve communication of multidimensional gait outcomes in routine care.

## 1 Introduction

Advances in digital health technologies are increasingly shaping the monitoring of chronic neurological diseases. Sensor-based systems such as wearable inertial sensors, mobile applications, and other digital tools enable systematic capture of health-related data between clinic visits, support longitudinal analyses, and may transform complex measurements into clinically actionable information. Evidence suggests that digital biomarkers and data-driven dashboards can facilitate clinical decision-making and enrich consultations (1, 2). At the same time, research on digital health technology development consistently shows that human-centered methods from requirements elicitation to iterative prototype testing are essential for acceptance, trust, and implementation in real-world care (3). Recent reviews also emphasize that remote monitoring approaches are gaining traction in neurological disorders, yet standardization and sustained integration into routine workflows remain major challenges (4, 5).

Multiple sclerosis (MS) is a chronic, progressive neurological disease characterized by pronounced heterogeneity in symptoms, disease trajectories, and functional impairment. In addition to physical disability and mobility impairment, pwMS frequently experience cognitive dysfunction, fatigue, visual disturbances, and mental or psychological symptoms such as depression and anxiety, all of which can substantially affect quality of life, daily functioning, and participation. Mobility is a particularly central domain: even subtle gait changes can meaningfully affect daily functioning and quality of life (6–8). Consequently, multidimensional monitoring strategies that combine clinical scales, imaging, and quantitative functional mobility assessment are increasingly relevant for individualized disease management (1, 2, 9).

Gait monitoring is also one of the most methodologically diverse areas in neurological care. A recent scoping review summarized over 100 studies and reported a wide range of approaches, including wearable sensors, smartphone-based tools, force plates, and laboratory-based analyses while again highlighting persistent barriers to standardization and clinical integration (10). Against this background, a standardized, multidimensional gait analysis (Dresden Protocol for Multidimensional Walking Assessment, DMWA) has been implemented as part of routine care at the MS Center Dresden. This protocol systematically captures gait parameters related to speed, endurance, balance, and qualitative gait characteristics using wearable sensor systems (11), typically at annual intervals. Importantly, patient-reported experience measures (PREMs) suggest that acceptance and perceived quality of such assessments depend not only on the measured metrics but also on how assessments are conducted and how results are communicated (12, 13). Building on this clinical routine, prior work has further shown that routinely collected gait data can be leveraged to identify characteristic gait phenotypes (14, 15). Together, these findings highlight the need for structures that consolidate heterogeneous mobility data and present them in an intuitive and clinically meaningful way.

Despite the availability of rich multidimensional gait data, there is still a lack of consolidated, patient-centered overviews that integrate heterogeneous metrics from different measurement systems in a coherent, comprehensible, and clinical interpretable visualization. In contrast to conventional clinical result reports, such an overview organizes parameters into clinically meaningful functional domains, supports longitudinal interpretation, and presents the information in a patient-friendly manner to facilitate understanding during clinical consultations. Work on dashboard development in rare diseases has demonstrated that early and continuous stakeholder involvement is critical to make complex data clinically manageable (16). Similar principles have been reported across other health domains, where workshops and interviews with end users were key to translating needs into concrete design requirements and producing practice-oriented dashboards (17). Evidence from clinical decision support research emphasizes that transparency and iterative, user-centered development are prerequisites for trust and uptake (18, 19). Additionally, studies on the visualization of single-patient health data indicate that visualization choices can directly affect comprehension and cognitive processing of clinical information (20). Finally, analyses of contextual (user) factors indicate that digital tools only achieve sustained impact when they are meaningfully integrated into existing workflows (21).

Against this backdrop, we developed a user-centered dashboard as a pilot solution for people with multiple sclerosis (pwMS), based on our established clinical routine of multidimensional gait analysis, and evaluated it in several cycles with patients and experts. The aim was to (i) develop a patient-centered dashboard that integrates multidimensional gait data into a coherent and intuitive overview, (ii) apply a human-centered design process aligned with routine clinical workflows, and (iii) evaluate the dashboard's comprehensibility, perceived usability, and perceived usefulness from the perspectives of medical professionals and pwMS.

## 2 Materials and methods

Reporting followed the STARE-HI guidelines for evaluation studies in health informatics; the completed checklist is provided as Supplementary Material S1. The work was carried out at the MS Center Dresden at University Hospital Dresden, a tertiary outpatient clinic specializing in multiple sclerosis care, where multidimensional gait analysis is routinely performed. The overall development and evaluation process was conducted between April 2024 and June 2025.

Participants provided informed consent as part of the standardized multidimensional gait analysis conducted at the MS Center Dresden (approved by the Ethics Committee of Dresden University of Technology; ethics approval number BO-EK-320062021). In accordance with the guidance of the Ethics Committee of Dresden University of Technology, the anonymous survey component of this study did not require additional formal ethics approval because only anonymous data were collected and processed. Nevertheless, all procedures involving human participants were conducted in line with the principles of the Declaration of Helsinki. Before participation, all respondents received verbal information about the purpose and content of the study, the voluntary nature of participation, and the handling of their data. Verbal informed consent was obtained from all participants prior to data collection. Participants were informed that they could withdraw from the study at any time without providing a reason and without any consequences for their medical care or legal status.

### 2.1 Overall approach

**Figure 1:**
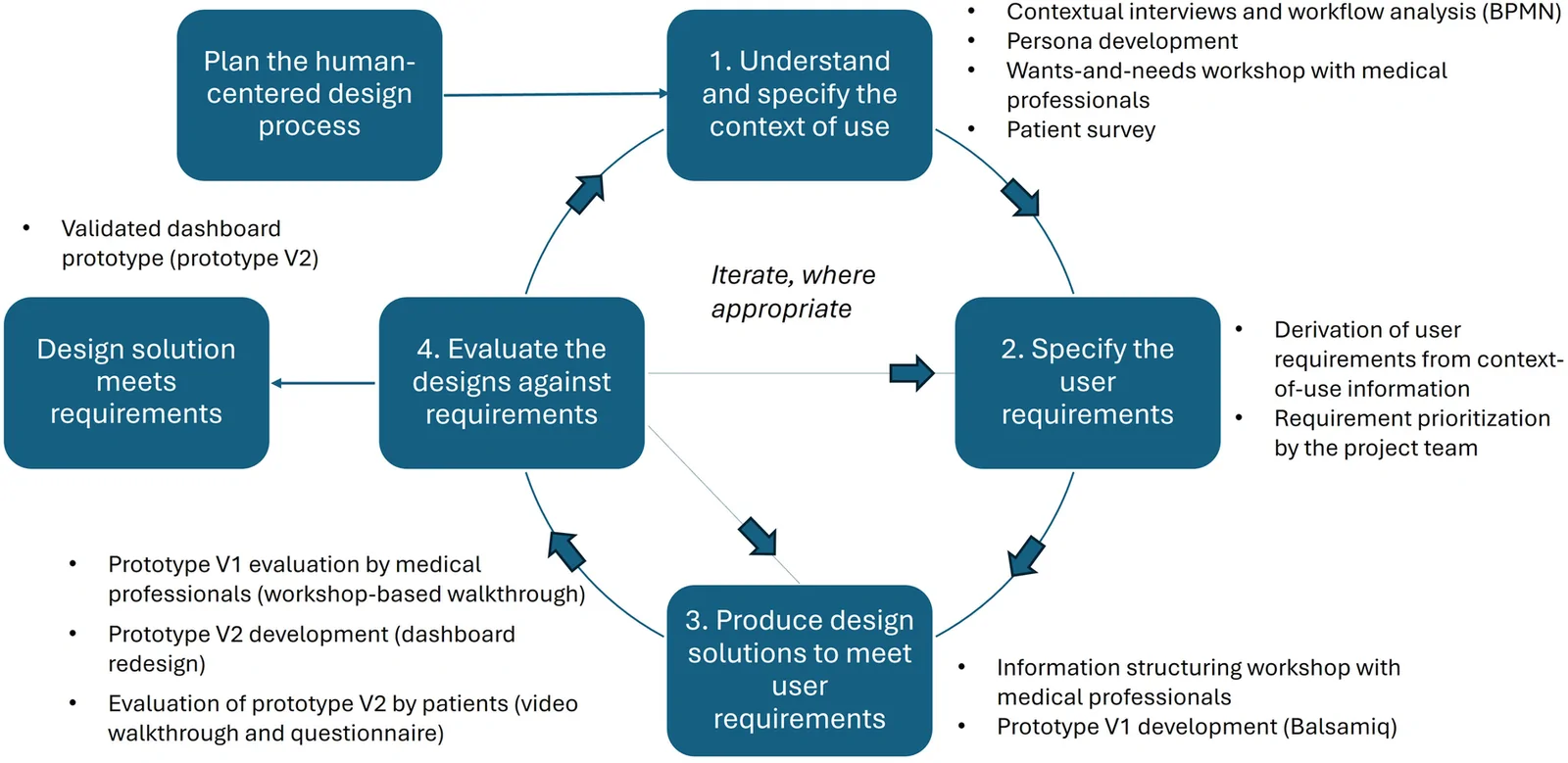
Overview of the iterative user-centered development phases and the methods applied.

To ensure the development of a user-centered and clinically relevant gait analysis dashboard, we applied a human-centered design (HCD) framework in accordance with DIN EN ISO 9241-210 (22). The process comprised four iterative phases: context analysis, requirements specification, design, and evaluation. Figure 1 summarizes the study flow from context analysis and requirements elicitation to iterative prototyping and formative evaluation with clinicians and patients.

### 2.2 Context analysis and requirements specification

The context analysis phase aimed to understand the clinical use context of the planned gait analysis dashboard. This included analyzing existing workflows and tasks related to multidimensional gait assessment, identifying relevant user groups and their characteristics, exploring informational needs and desired dashboard functionalities, and investigating patients' experiences and challenges regarding the interpretation and communication of gait analysis results in routine MS care. To achieve this, complementary human-centered design methods were applied, including workflow analysis, persona development, a wants-and-needs analysis with medical professionals, and a patient survey. Insights from these activities informed the subsequent derivation and prioritization of user requirements for the dashboard design.

#### 2.2.1 Workflow analysis

The context analysis phase aimed to understand workflows, needs, and challenges associated with gait analysis in MS care:

Workflows of the MS outpatient clinic were examined using contextual interviews (23) combining workplace observation and semi-structured interviews with medical professionals routinely involved in conducting, interpreting, and communicating gait analysis results. A three-person research team documented tasks, process steps, involved personnel, and software systems used during routine gait assessments. The workflow was represented as BPMN diagrams (https://www.bpmn.org/) to identify potential integration points for the planned gait analysis dashboard. Details of the BPMN specification were limited to core elements relevant for clinical interpretation.

#### 2.2.2 Persona development

Based on the contextual interviews and supplementary literature [e.g. (24, 25)], personas (26) were developed as archetypical stakeholder profiles. They capture key characteristics of medical professionals and pwMS relevant to gait data visualization and interpretation. Four personas were created: one physiotherapist rater and three patient personas representing different MS stages.

#### 2.2.3 Wants-and-needs analysis

A wants-and-needs analysis (27) was conducted through a workshop with medical professionals (*N* = 4) to understand how a gait analysis dashboard could best support clinical practice. Participants brainstormed relevant tasks and desirable dashboard characteristics, which were documented and then prioritized using a ‘Top 3’ booklet method. In this exercise, each participant ranked their three most important items and provided brief justifications. The ranked booklets were analyzed to identify the most frequently prioritized aspects, informing key user requirements for the dashboard.

#### 2.2.4 Patient survey

In parallel, an anonymous paper-based patient survey (*N* = 30) was conducted during routine clinic visits to explore patients' experiences and expectations regarding the presentation and interpretation of gait analysis results. Eligible participants were adults with confirmed multiple sclerosis who provided informed consent for the gait analysis and completed the questionnaire; patients with suspected acute clinical deterioration were not approached. The self-developed questionnaire addressed three areas: (a) satisfaction with current result displays (e.g., detectability, distinguishability, interpretability), (b) desired content and visualization preferences (e.g., relevant gait parameters and comparison options), and (c) expectations for interaction and accessibility (e.g., personalized result views and preferred access formats). Combining Likert-scale ratings and open-ended questions, the survey provided insights into patients' informational needs and priorities for designing a future gait analysis dashboard. Survey data were analyzed descriptively, and free-text responses were summarized thematically.

#### 2.2.5 Requirements specification

All collected information from contextual interviews, the workshop, and the survey was analyzed to identify user needs, goals, and challenges related to gait analysis data. From these insights, a set of user requirements was derived. Each requirement was documented in a structured template (‘The user should be able to see/click/enter/select X on the dashboard') with a brief explanatory note. The requirements were prioritized by the project team according to relevance and feasibility as (1) essential, (2) optional, or (3) for future projects.

### 2.3 Design and evaluation

The design and evaluation phase focused on translating the identified user requirements into iterative dashboard prototypes and evaluating them with intended end users. This included defining the dashboard's information architecture and visualization concepts, developing and refining successive prototype versions, and conducting formative evaluations with medical professionals and patients to iteratively optimize the dashboard design.

#### 2.3.1 Parameter categorization and initial design

A collaborative workshop with medical professionals (*N* = 2) was conducted to define the dashboard's information architecture. In the first part, gait parameters were compiled and grouped into categories and subcategories and prioritized using card sorting (28). In the second part, participants developed initial visualization ideas through sketching, drawing on example design patterns (29). The session was facilitated by a usability professional.

#### 2.3.2 Development of prototype V1

In this phase, conceptual ideas based on the prioritized requirements and personas were translated into a first user interface (UI) prototype of the gait analysis dashboard. To guide this process, the research team developed a design checklist for clinical dashboards, derived from a narrative literature review (PubMed/Google Scholar). Using this checklist, two usability professionals created a medium-fidelity UI prototype in Balsamiq (https://balsamiq.com/), defining the layout, content structure, and interaction elements of the dashboard. The design integrated the identified gait parameters and aimed to balance clarity, clinical relevance, and aesthetic simplicity. Color was used sparingly and only when conveying meaning, while detailed visual styling was intentionally omitted. To populate the prototype, the team used clinically plausible, non-identifiable example values defined with clinical experts rather than real patient data. The prototype formed the basis for formative feedback and iterative refinement.

In parallel, technical feasibility and integration requirement were explored through on-site meetings with medical professionals and consultations with the Data Integration Center of the University Hospital Dresden. Existing data sources, storage locations, export procedures, and information flows of the GAITRite and Mobility Lab systems were analyzed to identify requirements for future dashboard integration. Both systems currently operate independently and generate separate gait and balance reports that are manually combined for clinical review and research purposes. The feasibility assessment focused on potential strategies for automated data aggregation, longitudinal synchronization, secure transfer, and dashboard visualization. Preliminary architectural considerations included centralized storage of exported gait parameters in a structured database environment compatible with dashboard frameworks such as Apache Superset, automated monitoring of newly generated assessment files, and integration with existing clinical systems such as ORBIS. Future implementation will be designed to support established healthcare interoperability standards, such as HL7 FHIR, to facilitate standardized data exchange with existing clinical information systems and interoperable integration of multidimensional gait assessment data. These analyses confirmed the conceptual feasibility of the proposed dashboard design while also identifying data harmonization and interoperability as important challenges for future implementation.

#### 2.3.3 Clinician evaluation of prototype V1

Following the HCD framework, feedback on the dashboard prototype was collected during a workshop with medical professionals (*N* = 4). After a guided walkthrough of the Balsamiq prototype, participants reviewed the layout, navigation, and interaction flow using a structured set of guiding questions across four dimensions: (a) logic, (b) functionality, (c) information, and (d) terminology. They documented observations, identified improvement areas, and jointly prioritized issues as high, medium, or low importance. All feedback was documented directly in the prototype to ensure traceability.

To complement these qualitative insights, participants completed a short anonymous paper-based survey, rating the overall dashboard concept on a German 6-point school grading scale (1 = very good, 6 = insufficient) and providing brief written explanations. The prioritized feedback from this evaluation informed the next design iteration.

#### 2.3.4 Development of prototype V2

Based on the findings from the preceding evaluation, the dashboard prototype was revised by two usability professionals to further optimize the design and better align it with user needs. As in the initial design phase, Prototype V2 was created using Balsamiq, ensuring consistency in layout development and facilitating iterative refinement. The updated prototype served as the basis for subsequent validation. The design modifications implemented between Prototype V1 and Prototype V2 are summarized in Supplementary Material S2 to document the iterative refinement process and the rationale for individual design decisions.

#### 2.3.5 Patient evaluation of prototype V2

A final patient evaluation of the validated dashboard prototype (Version 2) was conducted at the MS Center with 24 participants. Participants were adults with a confirmed diagnosis of multiple sclerosis who were recruited during routine outpatient visits and were able to provide informed consent and complete a questionnaire-based evaluation. To ensure a consistent and comprehensible presentation, a narrated video walkthrough explaining the dashboard's structure, functions, and interpretation aids was created. Because the prototype was not yet technically implemented, the narrated walkthrough was intended to evaluate the conceptual information architecture, interpretability, and perceived usability of the dashboard rather than hands-on interaction performance or task completion behavior, and therefore did not constitute a formal usability test of interactive system use.

During routine clinic visits, patients viewed the video in a quiet room under supervision and then completed an anonymous paper-based questionnaire assessing comprehension, satisfaction, and preferences regarding the dashboard concept and information presentation. The questionnaire included two sections:

- Part 1: satisfaction with the new visualization (detectability, readability, clarity, interpretability, compactness, consistency, color design, aesthetics, terminology, overall satisfaction).
- Part 2: comparison with the previous display (perceived usability, intuitiveness, support for understanding results and health status, motivational impact, overall preference).

Open-ended questions captured qualitative feedback. All data were collected anonymously. Quantitative data were analyzed descriptively, and qualitative responses were summarized thematically. The questionnaire is provided as Supplementary Material S3.

## 3 Results

The development process yielded several key outcomes that reflect the collaborative and iterative nature of the HCD framework.

### 3.1 Results of the context analysis and requirements specification

#### 3.1.1 Workflow analysis

**Figure 2:**
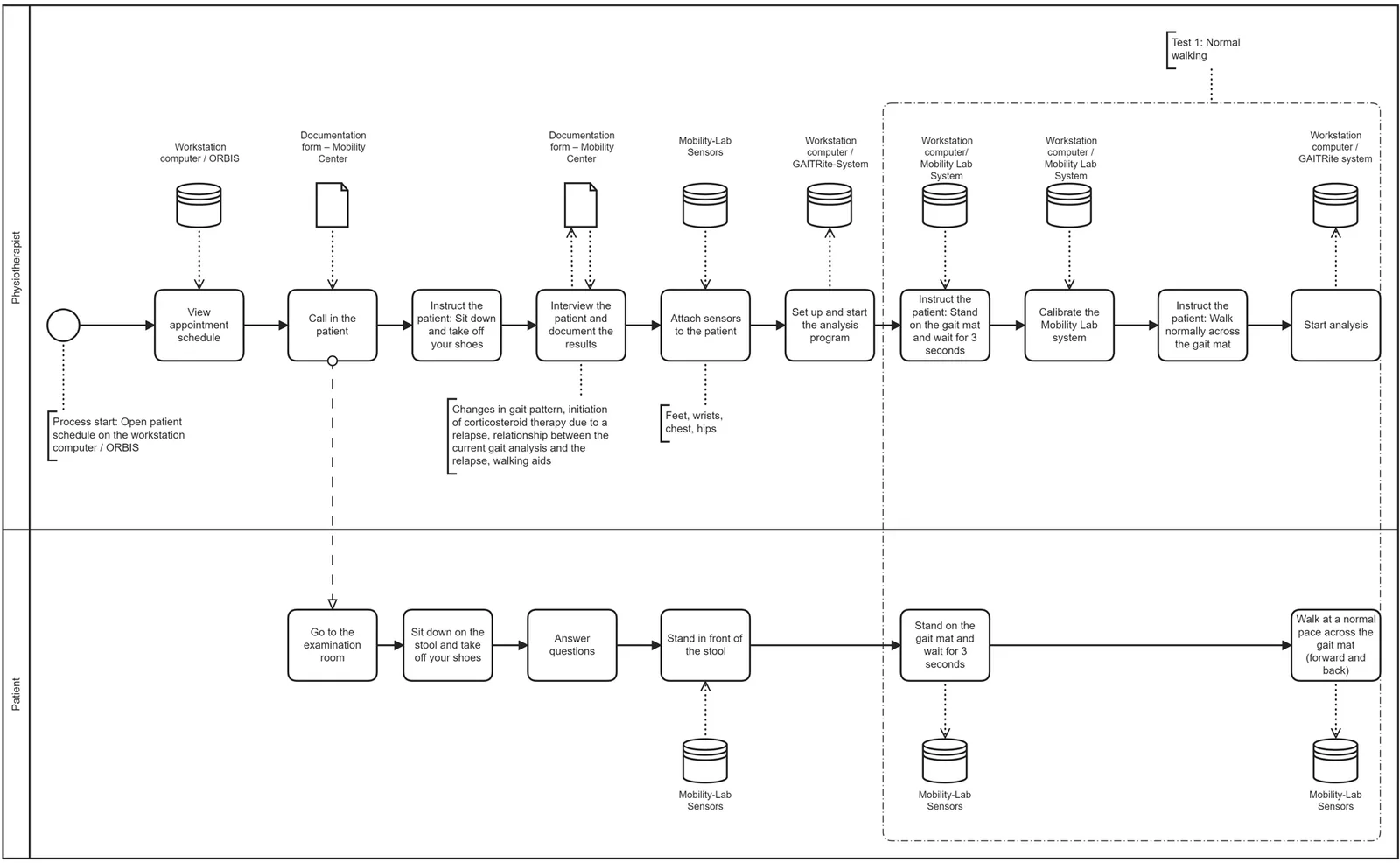
Excerpt from the BPMN model depicting the workflows for gait analysis at the MS center Dresden.

The workflow analysis, modeled as BPMN diagrams (excerpt in Figure 2), captured the complete sequence of clinical and documentation steps performed by physiotherapists during gait assessments in the MS Center. Each gait test is supported by the digital systems Mobility Lab (APDM Wearable Technologies Inc., Portland, USA, OPAL V2R) and GAITRite (CIR­ Systems, Inc., NJ, USA), which automatically generate PDF reports later linked to the electronic health record (EHR) ORBIS. The planned dashboard was conceived as a conceptual integration layer for these existing systems and reports, without modifying data acquisition, assessment, or documentation procedures.

The process begins with patient admission and pre-assessment preparation in ORBIS, followed by a short interview and sensor attachment. Subsequently, patients complete a standardized series of walking and balance tests according to the Dresden Protocol for Multidimensional Walking Assessment (DMWA) (11) and tablet-based self-report questionnaires (MSWS-12, EMIQ). Raters review the generated reports, compare results with previous assessments in ORBIS, provide brief feedback, and complete documentation for medical review. The analysis highlighted the need for a gait analysis dashboard that integrates automatically after completion of all sub-assessments, enabling medical professionals and patients to review a consolidated overview of results. It should also support retrospective review when abnormal findings are identified.

#### 3.1.2 Personas

**Figure 3:**
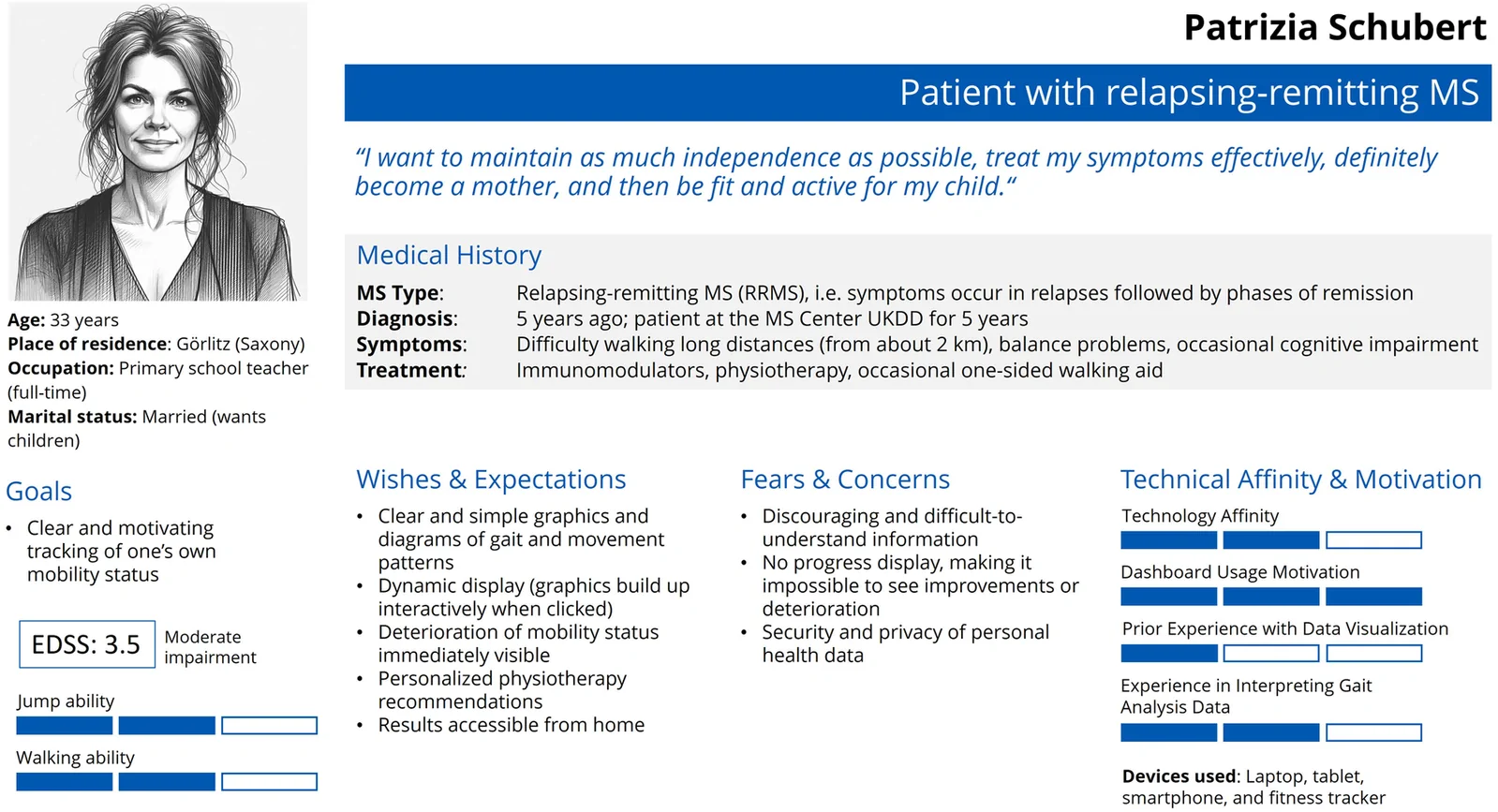
Persona (fictional composite) of a moderately impaired patient with multiple sclerosis. (AI-generated image; for illustrative purposes only).

Four personas were created to represent key stakeholder perspectives for the gait analysis dashboard. The mobility-unit rater persona centers on rapid interpretation, concise summaries, and integration into clinical routines, while the three patient personas reflect different MS stages and associated differences in mobility limitations and digital engagement. Across patient profiles, priorities included understandable terminology, intuitive visual cues, and meaningful longitudinal feedback. Figure 3 shows the moderately impaired patient persona; details on all personas are available in Supplementary Material S4.

#### 3.1.3 Wants-and-needs analysis

Medical professionals (*N* = 4) highlighted the need for access to previous assessments to enable temporal comparisons and track disease progression. The dashboard should provide a comprehensive overview of gait parameters from motion sensors and instrumented walkway, presented both qualitatively (e.g., color-coded thresholds) and quantitatively (e.g., reference ranges). Grouping results into functional domains such as balance, coordination, and asymmetry was considered essential for quick interpretation and clinical decision-making. Configurable reference values should allow adaptation to patient characteristics such as disease stage, age, or sex. Visual alerts were recommended to highlight deviations from reference ranges, and the dashboard should support individualized feedback or recommendations to guide exercise planning. An intuitive interface with consistent color coding, schematic visualizations, and simple navigation was considered crucial for routine clinical use. The system should also be technically flexible and scalable, allowing integration of new parameters, persistent data storage, immediate result availability, and export options for patient summaries.

Overall, the findings underline that the dashboard should combine clinical precision, longitudinal insight, and user-centered design to meet stakeholder expectations.

#### 3.1.4 Patient survey

Thirty patients participated in the survey (57% women, 33% men, 10% not specified). Most were aged 36–55 years, with moderate computer literacy (60%) and generally positive self-rated health (80% ‘good' or 'satisfactory'). The average time since MS diagnosis was 11.8 years (SD = 7.2), and most participants had completed three to ten gait assessments.

Patients were generally satisfied with the current digital presentation of gait analysis results but identified clarity and interpretability as the main areas for improvement. They requested simpler, German-language terminology, fewer technical expressions, clear definitions of abbreviations, and consistent use of measurement units within a consolidated display. High value was also attributed to comparisons with previous visits and reference groups, such as healthy controls, age-matched peers, or patients with similar impairments.

Although most participants did not favor a personalized display during consultations, many expressed interest in accessing their results after the visit, preferably as printable PDFs or via email. They also requested that gait results be accompanied by brief contextual explanations and specific therapeutic recommendations.

#### 3.1.5 Requirements list

A total of 22 requirements were identified (12 priority 1, 3 priority 2, 7 priority 3). The high-priority requirements covered core functionalities and usability aspects essential for clinical use and patient understanding. Central was an intuitive, clearly structured, and patient-oriented presentation of results that allows both medical professionals and patients to interpret gait parameters at a glance. The dashboard should display relevant parameters, integrate current and previous measurements in one view, and support longitudinal interpretation of treatment progress.

Key requirements addressed clarity and comprehensibility, including plain German language, consistent units, and avoidance of technical jargon. Visual cues and concise graphics were required to highlight clinically relevant deviations and organize parameters into functional domains. Further requirements included scalability, visualization of threshold values, automated feedback with simple recommendations, and efficient digital communication of results. A detailed list is provided in Supplementary Material S5.

### 3.2 Results of design and evaluation

#### 3.2.1 Parameter categorization and initial design preferences

During the workshop, medical professionals collaboratively defined and prioritized the core data categories and parameters for visualization, focusing on information essential for clinical interpretation, longitudinal follow-up, and patient communication.

**Patient-related data.** Although patient's personal data were not the primary focus of this workshop, several variables were considered indispensable for contextualizing gait results. These included the Expanded Disability Status Scale (EDSS) score, use of assistive devices (e.g., cane/rollator), and general descriptors such as patient ID, age, sex, and MS subtype. These data were seen as critical for interpreting performance outcomes in relation to disease stage and individual mobility aids.

**Gait analysis parameters.** The gait data were systematically grouped according to test type and functional gait domains. For the five gait assessments - normal walking, dual-task walking, fast walking, backward walking, and the 2-minute walk test (2MWT) - parameters were assigned to four overarching domains [according to (14, 30)]:

- Pace (e.g., walking speed, step/stride length, dual-task cost)
- Rhythm (e.g., cadence, step/stride time, single/double support)
- Variability (e.g., step-length and step/stride-time variability)
- Asymmetry (e.g., left–right differences in step/stride parameters).

Balance parameters from Romberg tests (eyes open/closed) were summarized under a fifth domain, Balance (e.g., sway area, anterior–posterior/lateral sway).

This multidimensional organization of gait parameters was informed by established gait frameworks and factor-analytic approaches reported in the literature. Prior factor-analytic studies have identified gait domains such as pace, rhythm, variability, asymmetry, postural control/base of support, and stability, and MS-specific work has further supported comparable domain structures in pwMS (14, 31–35).

**Initial design preferences.** Medical professionals agreed that parameters should remain test-specific rather than averaged across tests. Pace and Balance were rated as most clinically informative, followed by Rhythm, Variability, and Asymmetry. They proposed a hierarchical layout with a traffic-light domain summary highlighting critical deviations and a drill-down to domain- and parameter-level details with color-coded reference ranges; numerical values should be available on demand. The dashboard should support both within-patient longitudinal comparisons across repeated annual assessments and comparisons to normative/reference cohorts to facilitate interpretation and patient communication. For balance, medical professionals recommended a visualization similar to current reports, with optional tabular details if space is limited.

#### 3.2.2 Results of prototype V1 development and evaluation by medical professionals

Prototype V1 of the gait analysis dashboard was produced as an intermediate outcome of the first iteration and evaluated formatively by medical professionals (*N* = 4) in a workshop-based walkthrough. Overall, the design was rated positively, with all participants assigning a grade of ‘2 – good' on the German six-point scale. Medical Professionals emphasized the clear information structure, logical overview-detail alignment, and the integration of longitudinal and comparative reference data as major strengths.

Suggested refinements mainly concerned visual interpretability and layout optimization, including:

- clearer visual differentiation between left and right limb parameters,
- improved organization of the balance view,
- clearer delineation of the display range, showing only the upper and lower boundaries rather than the full normative range
- larger and more prominent main page headers, as well as additional section headings with detailed views, to improve readability and orientation,
- a less dominant visual style for peer-comparison symbols (e.g., greyscale instead of color), and
- displaying selected key values from each domain on the overview page instead of aggregated domain scores.

Despite an initially dense layout, the interface was perceived as intuitive after brief familiarization. This feedback informed the redesign leading to Prototype V2. A detailed overview of the clinician feedback and the resulting design modifications is provided in Supplementary Material S2.

#### 3.2.3 Results of prototype V2 development and patient evaluation

The final prototype comprised a hierarchically organized layout combining an overview page, test-specific detail views (including a dedicated balance section), and an aggregated recommendations section.

##### 3.2.3.1 Final dashboard prototype

**Figure 4:**
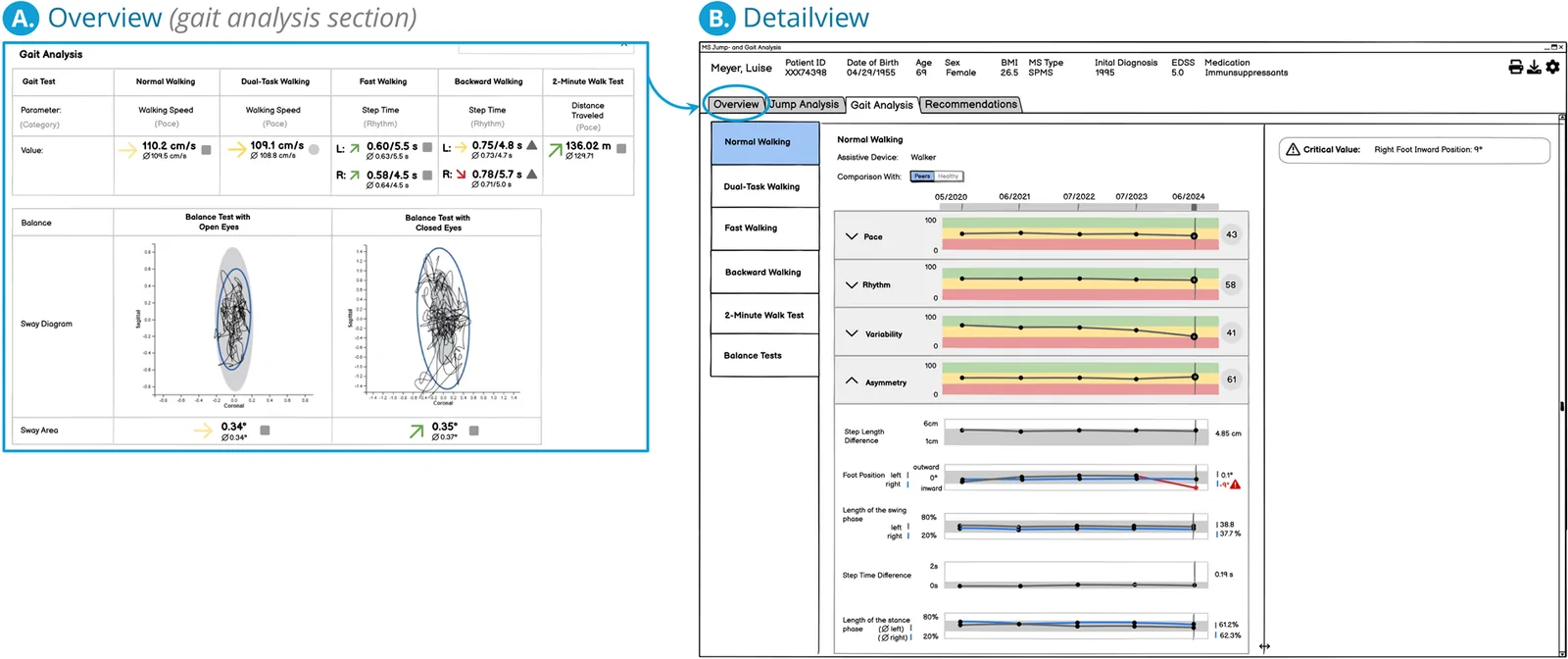
Overview **(A)** and detailed **(B)** page of the revised gait analysis dashboard prototype. (English translation of interface elements).

**Overview page.** The top-level view provided a compact summary of all gait and balance assessments, including normal walking, dual-task, fast, backward, and two-minute walking tests. For each test, the most relevant performance indicator (e.g., gait speed) was displayed alongside the patient's historical mean. An arrow indicated how the current value compared to previous tests - showing whether performance was better, similar, or worse. A geometric shape provided an at-a-glance indication of how the patient's performance compared to peers. Balance results were presented as sway diagrams, with the gray area indicating the reference range of healthy controls and the blue circle marking the patient's current sway area. Critical deviations were highlighted in red and accompanied by short, actionable recommendations. Patient identifiers (ID, age, sex, MS subtype, and EDSS score) were displayed in a fixed header area for contextual reference [Figure 4. Overview (A)].

**Detailed test view.** Each gait test could be expanded to display domain-specific trajectories for Pace, Rhythm, Variability, Asymmetry, and Balance across multiple visits - typically covering five to six annual assessments. Interactive time-series graphs presented the longitudinal progression of each parameter, while a horizontal time slider enabled precise selection of individual measurement points for accurate comparison between visits. The right-hand panel dynamically updated to show only the parameters and recommendations relevant to the currently selected domain, ensuring a focused view of key information. Parameters for the left and right limbs were displayed separately and clearly labeled to support the identification of potential asymmetries. Values exceeding predefined thresholds were highlighted in red and accompanied by warning symbols, facilitating the quick recognition of clinically relevant deviations. The toggle switch “Peers | Healthy (“Gesunde”)” allowed users to change the comparison group, with the colored areas in the charts updating accordingly to reflect the respective reference ranges [Figure 4. Detailview (B)].

**Balance section.** In addition to the gait assessment views, the dashboard provided a dedicated visualization for balance assessments, capturing results from the Romberg tests under eyes-open and eyes-closed conditions. Each plot displayed the patient's sway pattern over time, overlaid with reference ranges for peers and healthy controls. Critical deviations (e.g., excessive lateral sway) were highlighted, and zoom functionality supported detailed inspection.

**Summary and recommendations.** A dedicated recommendations section aggregated all current critical parameters and provided corresponding improvement suggestions (e.g., ‘train balance’, ‘increase left-leg stability'). Historical assessments could be retrieved via a date selector, supporting review of longitudinal changes.

A comprehensive description of all dashboard components, navigation pathways, and user interactions is available in Supplementary Material S6.

##### 3.2.3.2 Patient evaluation results

Participant characteristics are summarized in Table 1 (*N* = 24). Participants covered a broad age range and heterogeneous educational and clinical backgrounds.

**Table 1:** Demographic and clinical characteristics of patients included in the dashboard evaluation (prototype V2, *N* = 24). Values are reported as n (%) unless otherwise stated. Percentages may not sum to 100 due to rounding.

Category	n	%
Sex		
Male	7	29.17
Female	17	70.83
Age (years)		
26–35	6	25.00
36–45	7	29.17
46–55	4	16.67
56–65	4	16.67
66–75	2	8.33
76–85	1	4.17
Highest completed education		
No vocational or academic qualification completed	1	4.17
Completed vocational training	9	37.50
Master craftsman/technician qualification or degree from a vocational or professional academy	5	20.83
Academic degree	7	29.17
Other educational qualification	2	8.33
Computer skills/knowledge		
Low (many systems difficult to use)	2	8.33
Moderate (able to use most systems well)	13	54.17
High (very experienced/technically proficient)	9	37.50
Self-rated health status		
Very good	5	20.83
Good	6	25.00
Satisfactory	10	41.67
Poor	3	12.50
Number of previous gait analysis appointments		
1–2	2	8.33
3–5	14	58.33
6–10	8	33.33
Disease duration (years)	Mean (SD)	
Time since MS diagnosis	10.6 (8.4)	—
Functional status (self-reported)		
0-Normal function	9	37.50
1-Mild disability	5	20.83
2-Moderate disability	5	20.83
3-Gait impairment	3	12.50
4-Occasional use of a walking stick	1	4.17
5-Constant use of a walking stick	1	4.17

**Perception of the dashboard's information presentation.** Across all evaluated dimensions - detectability, legibility, freedom from distraction, distinguishability, interpretability, compactness, consistency, color design, aesthetics, and terminology - the dashboard was rated positively. Using a three-point scale (1 = ‘does not apply at all,’ 2 = ‘partly applies,’ 3 = ‘fully applies'), median ratings across all items were consistently high (median = 3), with interquartile ranges (IQRs) predominantly between 0 and 1, indicating low variability and strong agreement and overall satisfaction with the dashboard concept. Qualitative feedback focused on minor refinements to enhance clarity and visual comfort. It was suggested that gait speed be displayed in kilometers per hour instead of centimeters per second to improve interpretability. Patients also suggested that section headings such as Normal Walking should appear in a larger and more prominent format to facilitate orientation within the dashboard. The color-coded comparison symbols (green/yellow/red) were described as useful but could be made visually more distinctive. The overall color scheme was recommended to be softened by reducing the large white background in favor of light grey or blue tones to improve visual comfort during result discussions. It was further emphasized that key information should be highlighted more clearly, for example by using yellow accents or stronger contrast for critical values. The peer comparison indicator was perceived as inconspicuous due to its small grey font, and a clearer presentation was recommended. Additional comments included the suggestion to integrate short explanatory tooltips - for instance, a ‘?’ symbol next to the Peers label to clarify the reference group - and to provide an optional simplified view displaying fewer parameters to support patient comprehension.

**Figure 5:**
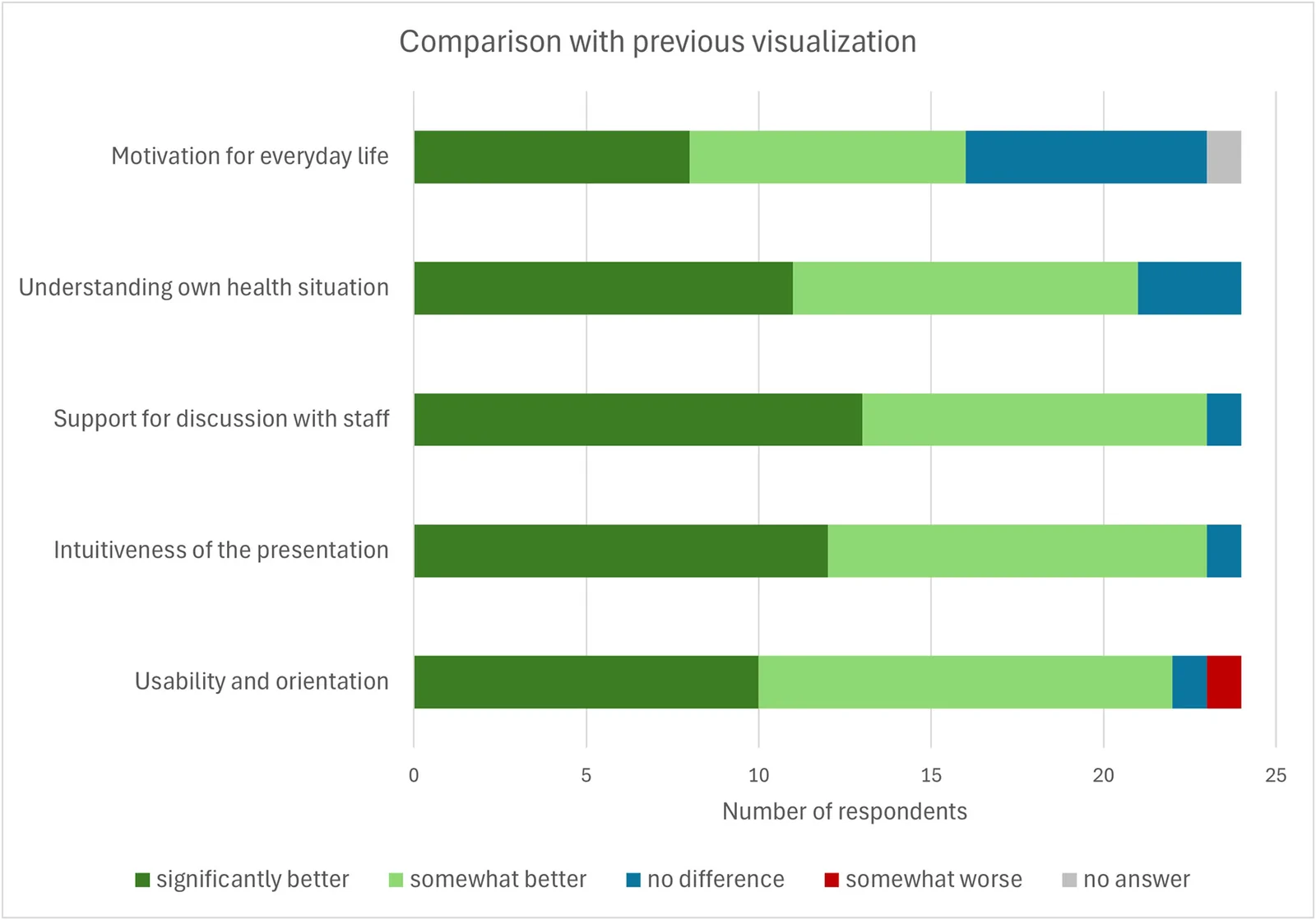
Comparative user ratings of the new vs. previous visualization across five evaluation dimensions.

**Comparison with the Previous Visualization.** Compared with the previous gait analysis result display, patients rated the new dashboard more favorably across all assessed dimensions, including perceived usability and orientation, intuitiveness, support for consultations, understanding of health status, and motivation for action. Results are summarized in Figure 5. When asked for an overall preference, 21 participants (87.5%) favored the new dashboard, two (8.3%) preferred the old version, and one (4.2%) reported no clear preference.

Taken together, these findings demonstrate that the revised visualization concept substantially enhanced perceived usability, interpretability, and patient engagement in the interpretation of gait analysis results, highlighting its potential for integration into routine MS care.

## 4 Discussion

### 4.1 Principal findings and contribution

This study set out to develop a patient-centered gait analysis dashboard for pwMS based on routinely collected data from multidimensional walking assessment using the DMWA protocol, and to iteratively validate its design with intended end users. The findings indicate that, within the scope of a formative mock-up–based concept evaluation, a human-centered, workflow-anchored visualization concept can substantially improve how pwMS perceive and interpret complex gait data. Following refinement based on professional feedback, prototype V2 was consistently perceived by patients as easier to understand and follow, more intuitive, better suited for clinical conversations, and more motivating for daily-life action than the previously used visualization. The strong overall preference for V2 suggests that careful alignment of gait domains, terminology, and visual hierarchy with user needs meaningfully enhances interpretability and perceived usefulness of gait outcomes in MS care. From a human-system interaction perspective, the positive ratings regarding intuitiveness, orientation, and overall satisfaction indicate that the dashboard was well received by patients. Although this formative evaluation did not explicitly assess technology acceptance using validated instruments, these findings are conceptually consistent with established acceptance frameworks such as the Technology Acceptance Model (TAM) (36) and the Unified Theory of Acceptance and Use of Technology (UTAUT) (37), which identify perceptions of usefulness and ease of use as important factors influencing the adoption of digital systems. However, because these constructs were not directly measured, the present study does not permit conclusions regarding technology acceptance as defined by these theoretical frameworks. At the same time, technology acceptance is influenced by additional factors beyond usability alone, including facilitating conditions, integration into clinical routines, visibility and interpretability of system outputs, and individual motivational aspects, which should be further considered in future implementation and evaluation studies.

An additional finding of this study concerns the iterative nature of human-centered design. Several design modifications that successfully addressed clinicians' feedback revealed further opportunities for refinement when subsequently evaluated by patients. For example, reducing the visual prominence of the peer-comparison symbols addressed clinicians' concerns that these elements were overly dominant, whereas some patients subsequently perceived them as too inconspicuous. Rather than representing contradictory stakeholder requirements, these findings illustrate how successive evaluations with different stakeholder groups provide complementary perspectives that support the progressive refinement of the dashboard design.

### 4.2 Interpretation in light of related work

Existing research on digital gait assessment and visualization in MS provides important building blocks, but often addresses only parts of a fully human-centered design trajectory:

Giunti et al. (25) demonstrate that successful mHealth solutions for physical activity in MS must be rooted in MS-specific symptoms, everyday barriers, and motivational drivers. Through mixed methods with pwMS and healthcare professionals, they derive four MS personas and identify desired features as well as adoption facilitators and obstacles. However, their contribution remains largely at the level of needs elicitation and persona development, without advancing into iterative interface design, prototyping, or usability validation with pwMS. Anwary et al. (38) pursue a more technology-driven approach by developing an IMU-based wearable gait analysis system and proposing multiple complementary visualizations (e.g., real-time bilateral dials, variability views, asymmetry comparisons, boxplot summaries, and aggregated gait-shape representations) to detect deviations and asymmetries. Yet, because systematic evaluation with patients or clinicians is not reported, it remains unclear how understandable, meaningful, or actionable these representations are for clinical communication or for pwMS. A similar expert orientation characterizes KAVAGait (39), which integrates automated data processing with clinicians' domain expertise and a knowledge store to aid storage, inspection, and comparison of complex gait datasets. The system is validated via expert reviews, a clinician user study, and a case study, indicating efficiency gains for professional interpretation, but its visual encodings and interactions are designed for expert decision support rather than for explaining results to patients, and no evaluation with pwMS is provided. Conceptually, Schniepp et al. (30) offer a strong clinical basis by reducing numerous spatiotemporal gait parameters into five independent domains (Pace, Rhythm, Variability, Asymmetry, Balance) using Principal Component Analysis (PCA) and normalizing domain scores against age- and sex-matched healthy controls. This enables clinician-facing diagnostic profiling and comparison to normative references, but the framework is not tailored to patient understanding. Finally, Voigt et al. (40) illustrate both the promise and a recurring limitation of participatory digital MS care: after surveys and co-design workshops with pwMS and professionals, they implement an MS patient portal, but formal usability and acceptability validation is explicitly deferred to future work. This highlights a broader HCD risk in digital health - early implementation can lock in designs before patient-facing communication has been thoroughly tested and refined.

Taken together, prior work shows rapid progress in needs elicitation, sensing technologies, and clinician-oriented analytics, but limited evidence on how to translate multidimensional gait outcomes into patient-understandable and actionable feedback through iterative end-user validation. Addressing this gap is the central contribution of the present study.

### 4.3 Strengths and limitations

A key strength of this study is the systematic application of a HCD process aligned with ISO 9241-210 across multiple iterative stages, rather than limiting user involvement to a single late usability test. ISO 9241-210 recommends cycles that begin with understanding users and context, translate insights into requirements, and repeatedly evaluate and refine designs to improve effectiveness, efficiency, and satisfaction. In digital health, early and iterative HCD is considered crucial for reducing innovation risk and avoiding premature design lock-in (41). Neurology-focused digital health research further emphasizes the importance of patient and public involvement, while pointing to persistent gaps in consistent guidance on implementation and reporting (42). Against this background, our work combined contextual analyses, structured requirements derivation with both stakeholder groups, iterative mock-up prototyping, formative refinement with clinicians, and a mock-up evaluation with pwMS. Such staged, mock-up–driven pathways are particularly valuable in mHealth, as they tend to increase usability and alignment with user needs-key prerequisites for later adoption (43). A further strength lies in the deliberate integration of complementary perspectives: medical professionals contributed clinical interpretability and workflow constraints, while pwMS brought everyday priorities and communication needs. Multi-stakeholder participation is widely recommended in digital health to improve clinical workability, patient acceptability, trust, and uptake (44). The iteration from prototype V1 to V2 was transparently grounded in prioritized expert feedback, providing a traceable rationale and reflecting core HCD principles (22) as well as established design-study guidance that highlights the value of documented expert-driven iteration for strengthening credibility (45). The subsequent patient evaluation further demonstrated that design modifications successfully addressing one stakeholder group's feedback may reveal additional opportunities for refinement when assessed by another user group. This iterative refinement process, documented in Supplementary Material S2, illustrates how complementary perspectives from clinicians and patients contributed to progressively optimizing the dashboard design in accordance with human-centered design principles. Finally, although the evaluation was based on unprogrammed mock-ups, the study offers conceptual evidence that domain-based, simplified gait feedback can be made patient-understandable. Low-fidelity testing is explicitly encouraged to refine information architecture prior to implementation (22) and is regarded as a cost-effective safeguard in digital health (46).

At the same time, limitations constrain generalizability. The evaluation relied on a synthetic scenario with artificial gait data rather than real patient cases. While this ensured standardization and privacy, it may have reduced realism, emotional engagement, and perceived relevance, and user judgments might differ with personal longitudinal results. Such ecological-validity concerns are well documented when study materials diverge from real use contexts (47), and synthetic data can entail fidelity and representativeness trade-offs that affect interpretation (48). Sample sizes were appropriate for exploratory HCD, but remain tied to local context and recruited profiles; the patient study (*N* = 24) supports usability insights but limits subgroup analyses. Reviews of digital-health usability research emphasize that such small samples are typical early on, yet broader validation is needed for generalizable claims (49). Moreover, pwMS evaluated the mock-ups via guided walkthroughs instead of hands-on interaction, which is suitable for early concept checks but may underestimate navigation errors, cognitive load, and learning effects that surface in real use (50). In addition, the narrated video format may have introduced presentation bias by guiding attention and interpretation in a more structured manner than would occur during independent real-world use. In particular, the comparison between the revised dashboard and the previously used clinical result reports may have been influenced by differences in presentation format. While the revised dashboard was introduced through a structured narrated walkthrough explaining visualization elements and interpretation aids, the previous visualization was evaluated primarily based on patients' prior experiences and recollections from routine care. Consequently, part of the observed preference for the revised dashboard may reflect the explanatory presentation context in addition to differences in the visualization concept itself. Consequently, the study cannot provide conclusions about actual interaction performance, navigation efficiency, task completion behavior, or error-related usability aspects that require direct hands-on system use. Furthermore, the study also did not explicitly assess how cognitive impairment, mental/psychological symptoms, or visual dysfunction may influence interaction with or interpretation of the dashboard. Although efforts were made to support comprehensibility through simplified terminology, structured information presentation, and reduced visual complexity, the specific impact of these MS-related impairments on dashboard usability was not systematically evaluated and should be addressed in future studies. At the same time, the patient sample included individuals with heterogeneous clinical and educational backgrounds, and overall ratings of clarity, interpretability, and usability remained consistently positive, which may indicate that the dashboard concept was understandable across a broad range of patient profiles. Because the artifacts were not implemented, we cannot assess technical feasibility, data integration, system performance, or routine workflow acceptance; mock-ups primarily support early concept validation rather than deployment-level testing (22). Finally, requirements and design decisions were derived in a specific clinical environment and DMWA routine, so transfer to other settings may require adaptation to different protocols, reference values, or communication practices, in line with the context-of-use emphasis of HCD frameworks. Although the selected domain structure was informed by established multidimensional gait frameworks from the literature, the final parameter prioritization and visualization logic were additionally refined in collaboration with a relatively small group of local clinical experts. Broader multi-center clinical validation will therefore be important to evaluate transferability and consensus regarding parameter weighting, threshold interpretation, and communication strategies across neurological care settings. An additional limitation relates to the mid-fidelity nature of the prototypes. Visual design elements were intentionally simplified, with color applied primarily when it conveyed semantic meaning, while other elements were kept largely in neutral tones. Although this approach aligns with best practices for early-stage prototyping and supports focus on information structure and interpretability rather than visual polish, it may have constrained the assessment of aesthetic quality, emotional response, and visual engagement, which are known to influence overall user experience (51). However, given that both patients and clinicians still rated the dashboard's clarity and visual appeal positively, this suggests that the core design concepts were sufficiently robust and that the reduced visual fidelity did not substantially impair perceived usability or acceptance at this stage.

### 4.4 Clinical meaning and generalizability

The clear preference for the revised dashboard suggests that pwMS can meaningfully engage with multidimensional gait results when these are aggregated into clinically relevant domains, presented in a coherent overview-to-detail structure, and explained using accessible terminology and recommendations. This is clinically important because gait and mobility outcomes are central for monitoring disease progression, rehabilitation needs, and treatment response in MS and other neurological disorders, yet quantitative gait metrics are often difficult to translate into implications that patients can understand (52–54). Transparent domain-level trends and deviations may therefore strengthen clinical communication: clinicians can more clearly explain why changes matter, and patients may better relate objective findings to lived experience (e.g., fatigue-related slowing, balance instability, or asymmetrical stepping). This aligns with broader work on shared decision-making in MS, which emphasizes the importance of understandable information, patient involvement, and preference-sensitive clinical communication (55, 56).

Beyond in-clinic communication, such visualization concepts may also support self-management if patient access is enabled in future implementations. While the present mock-ups were explicitly designed for use during structured clinical encounters, related work has explored how pwMS and other patient groups engage with mobility data derived from wearable sensors in everyday life. Using a Delphi-type, patient-led co-creation approach, Lumsdon et al. (57) showed that patients are generally able to interpret real-world mobility visualizations, while also highlighting the importance of contextual information, personalization, and clear justification of why specific outcomes are shown. Similarly, qualitative work on self-tracking and remote monitoring in MS suggests that patients and clinicians require not only numerical data but also contextual explanations that make mobility trends meaningful for daily life and care decisions (58). In contrast, our study focuses on standardized, laboratory-based gait assessments embedded in routine clinical workflows. Accordingly, effects on behavior or self-monitoring outside the clinic cannot yet be inferred. Still, providing understandable, domain-based summaries after the visit (e.g., as printouts or via a mobile application) could plausibly encourage reflection on mobility changes and engagement with rehabilitation or physical activity, which should be examined in implemented, longitudinal evaluations that bridge clinical and real-world contexts.

In terms of generalizability, the domain structure is physiologically not MS-specific. Established gait-domain frameworks describe dimensions such as pace, rhythm, variability, asymmetry, and postural control/base of support across older adults and neurological populations (31–33). MS-specific studies have further supported comparable multidimensional structures and their clinical relevance in pwMS (14, 34). Therefore, the visualization logic is likely transferable to other cohorts with gait and balance impairments, including Parkinson's disease, ataxias, stroke, or older adults with balance disorders, provided that disease-specific parameter weighting, thresholds, normative reference sets, and recommendation content are adapted (52, 54). Transfer to other centers will also depend on local gait protocols and data pipelines: because our concept builds on routine DMWA outputs, sites using different tasks, sensors, or reference values would need a brief context analysis to map their parameters onto the domains and align the views with local workflows. Thus, the study offers a generalizable design and communication framework rather than a one-size-fits-all solution, making multi-site adaptation and evaluation a key next step.

### 4.5 Implications and future work

Building on these findings, the next step is to translate the dashboard into a fully implemented system and integrate it into the existing gait analysis infrastructure. Only with real data pipelines, clinical interfaces, and stable rendering in place can feasibility be judged under routine conditions, including workflow fit, reliability of automated scoring, and maintenance demands. This includes the implementation of automated data synchronization pipelines, interoperability mechanisms between heterogeneous gait assessment systems, and integration into existing clinical information infrastructures using established interoperability standards such as HL7 FHIR. Once implemented, evaluation should shift from video walkthroughs to hands-on use in real consultations. Observing pwMS and clinicians interacting with the dashboard *in situ* will allow a more realistic assessment of navigation, cognitive load, time requirements, and, crucially, its communicative value for shared understanding and clinical decisions. After technical feasibility and in-clinic usability are established, longitudinal studies are needed to test whether repeated domain-based feedback influences longer-term outcomes such as patients' understanding of mobility changes, confidence in discussing gait issues, adherence to rehabilitation, or motivation for physical activity - effects that cannot be inferred from the present mock-up-only, clinic-bound evaluation.

### 4.6 Conclusion

In conclusion, this study shows that a workflow-anchored human-centered design pathway can yield a patient-facing gait dashboard that pwMS experience as understandable and clinically meaningful despite the complexity of multidimensional walking assessment results. Beyond the interface itself, the project provides a transparent, reproducible methodological pathway from context and workflow analysis and requirements elicitation to iterative mock-up refinement and end-user evaluation that may inform the development of patient-oriented visualizations in neurology and digital health. The revised prototype therefore represents a formatively evaluated design concept for subsequent work, either through technical implementation within routine gait-assessment infrastructures or through adaptation of its domain-based communication logic to other conditions and care contexts. Because the current evidence is limited to mock-up usability testing with synthetic data, real-world implementation and longitudinal *in-situ* evaluation remain essential to confirm feasibility, workflow integration, and impact on clinical communication and patient engagement.

## Data Availability

The raw data supporting the conclusions of this article will be made available by the authors, without undue reservation.
