# Supplementary material for: User-centered design and formative evaluation of a gait dashboard for multidimensional walking assessment in multiple sclerosis
Source: Front Digit Health. 2026 Sep 8;8:1865736. doi: 10.3389/fdgth.2026.1865736 (PMC13593768; doi:10.3389/fdgth.2026.1865736)
Supplement: Supplementary file 1 [file Datasheet1.pdf]

Stare-HI Checklist

Checklist based on: Brender, J., Talmon, J., de Keizer, N., Nykänen, P., Rigby, M., & Ammenwerth, E. (2013). STARE-HI–statement on reporting of evaluation studies in health informatics. *Applied clinical informatics*, *4*(03), 331-358.

STARE-HI Item		Item comprised in manuscript?
Title		Yes
Abstract		Yes
Keywords		Yes
Introduction	Scientific background	Yes
	Rationale for study	Yes
	Objectives of the study	Yes
Study context	Organisational setting	Yes
	System details and systems in use	Yes
Methods	Study design	Yes
	Theoretical background	Yes
	Participants	Yes
	Study Flow	Yes
	Outcome measures or evaluation criteria	Yes
	Methods for data acquisition and measurement	Yes
	Methods for data analysis	Yes
Results	Demographic and other study coverage	Yes
	Study findings and outcome data	Yes
	Unexpected observations	Not applicable
Disussions	Answer to study questions	Yes
	Strenghs and weaknesses of the study	Yes
	Results in relation to other studies	Yes
	Meaning and generalisability of the study	Yes
	Unanswered and new questions	Yes
Conclusion		Yes
Authors´contribution		Yes
Competing interests		Yes
Acknowledgement		Yes
References		Yes
Appendices		Yes
