# Supplementary material for: User-centered design and formative evaluation of a gait dashboard for multidimensional walking assessment in multiple sclerosis
Source: Front Digit Health. 2026 Sep 8;8:1865736. doi: 10.3389/fdgth.2026.1865736 (PMC13593768; doi:10.3389/fdgth.2026.1865736)
Supplement: Supplementary file 2 [file Datasheet2.pdf]

Supplement S2. Iterative Design Refinement from Prototype V1 to Prototype V2

Key findings and suggestions from the evaluation by medical professionals (Prototype V1) and resulting design changes (Prototype V2)

<table>
<tr><th>Suggestions / Key findings (Prototype V1)</th><th>Change made (Prototype V2)</th></tr>
<tr><td colspan="2">Header</td></tr>
<tr><td>The header should be displayed slightly larger.
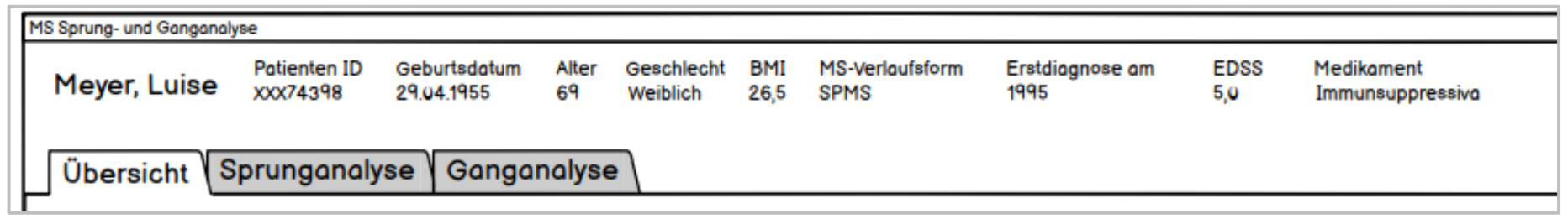
</td>
<td>The header font size was increased to improve visibility.
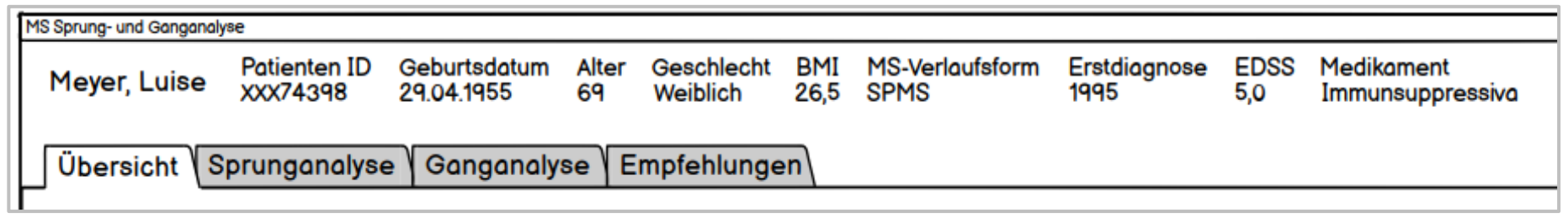
</td></tr>
<tr><td>Remove the word "am" (engl. "on“) from "Erstdiagnose" (engl.: „initial diagnosis“).
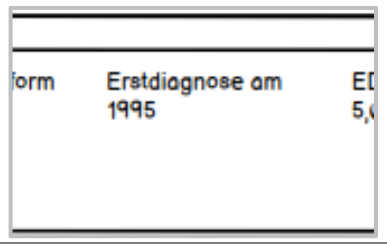
</td>
<td>The word "am" was removed from "Erstdiagnose" ("initial diagnosis").
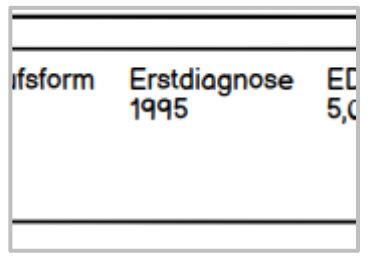
</td></tr>
<tr><td colspan="2">Overview page</td></tr>
<tr><td>Instead of initially presenting the domains (Speed, Rhythm, Variability, and Asymmetry), display only the following key measures: normal walking – velocity, dual task - velocity, 25-Foot Walk - time (s), 2-Minute Walk - distance (m), and 3-m backward walking - time (s). (The collapsible domain structure can be retained on the subsequent pages.)
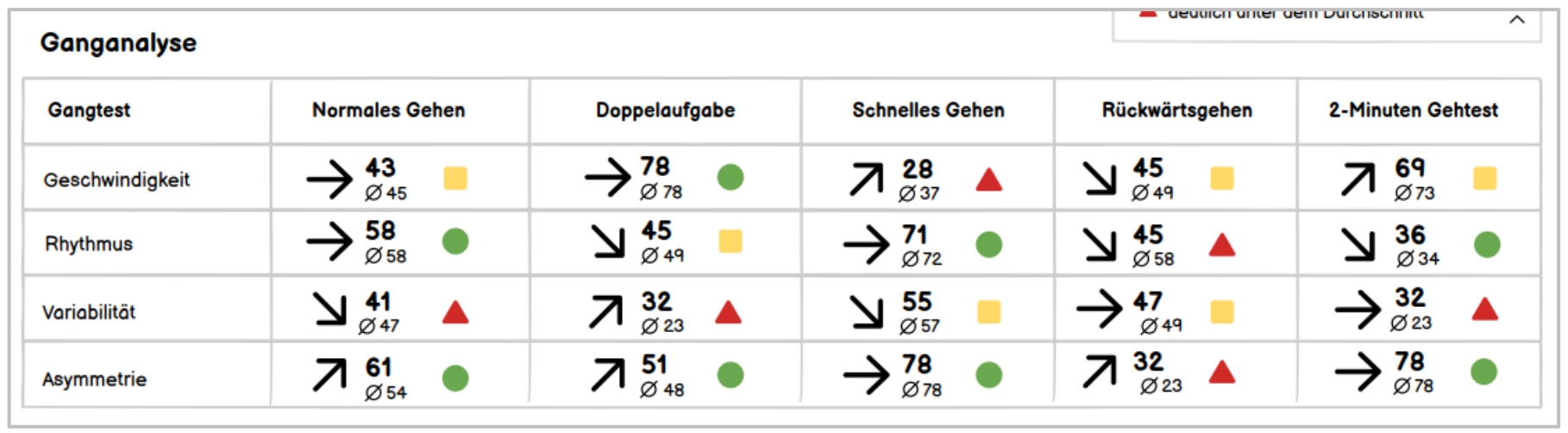
</td>
<td>Instead of displaying overall scores, changes, and comparisons across domains, only the key measures for the respective domain are now displayed.
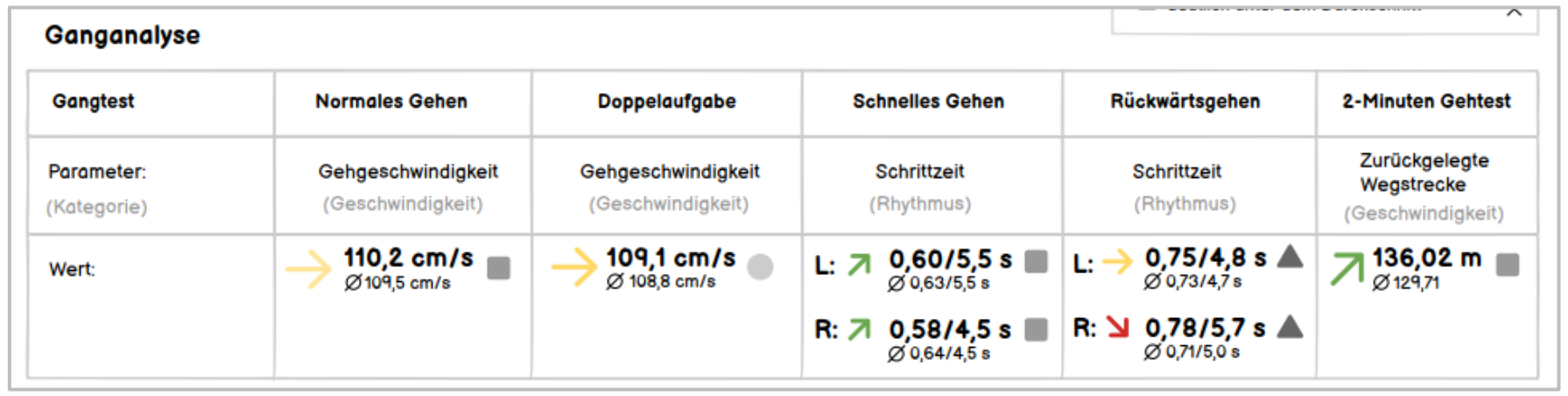
</td></tr>
<tr><td>Highlight the arrows (change compared with the previous visit) using colors, while making the symbols (peer comparison) less visually prominent.
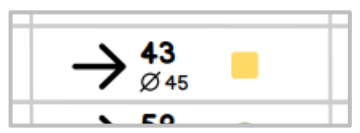
</td>
<td>The arrows (change compared with the previous visit) were highlighted using green, yellow, and red, while the symbols (peer comparison) were displayed in dark, medium, and light gray.
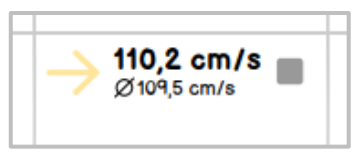
</td></tr>
</table>

<table>
<tr><th>Suggestions / Key findings (Prototype V1)</th><th>Change made (Prototype V2)</th></tr>
<tr><td>
Add the range of motion value and include the corresponding indicators used for the other parameters (arrow = change, symbol = peer comparison).

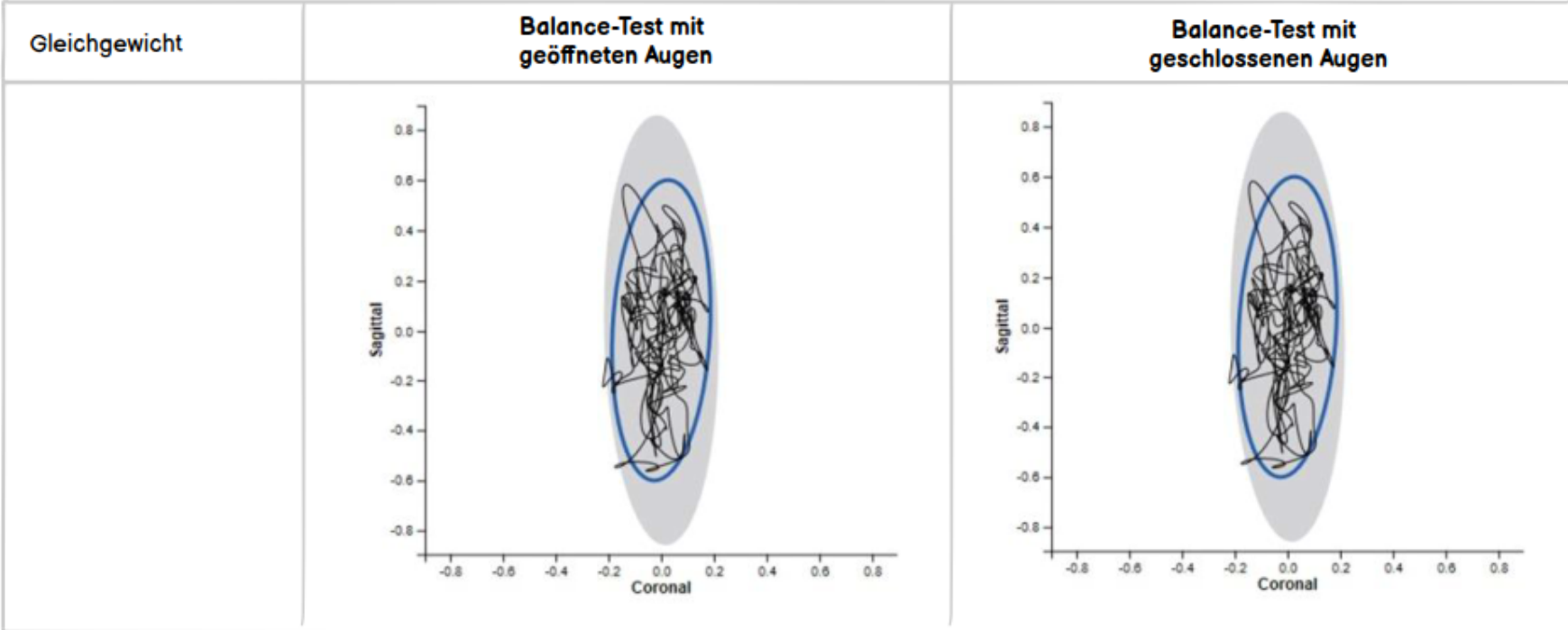

</td><td>
The range of motion value was added, including the change indicator (arrow) and peer comparison symbol

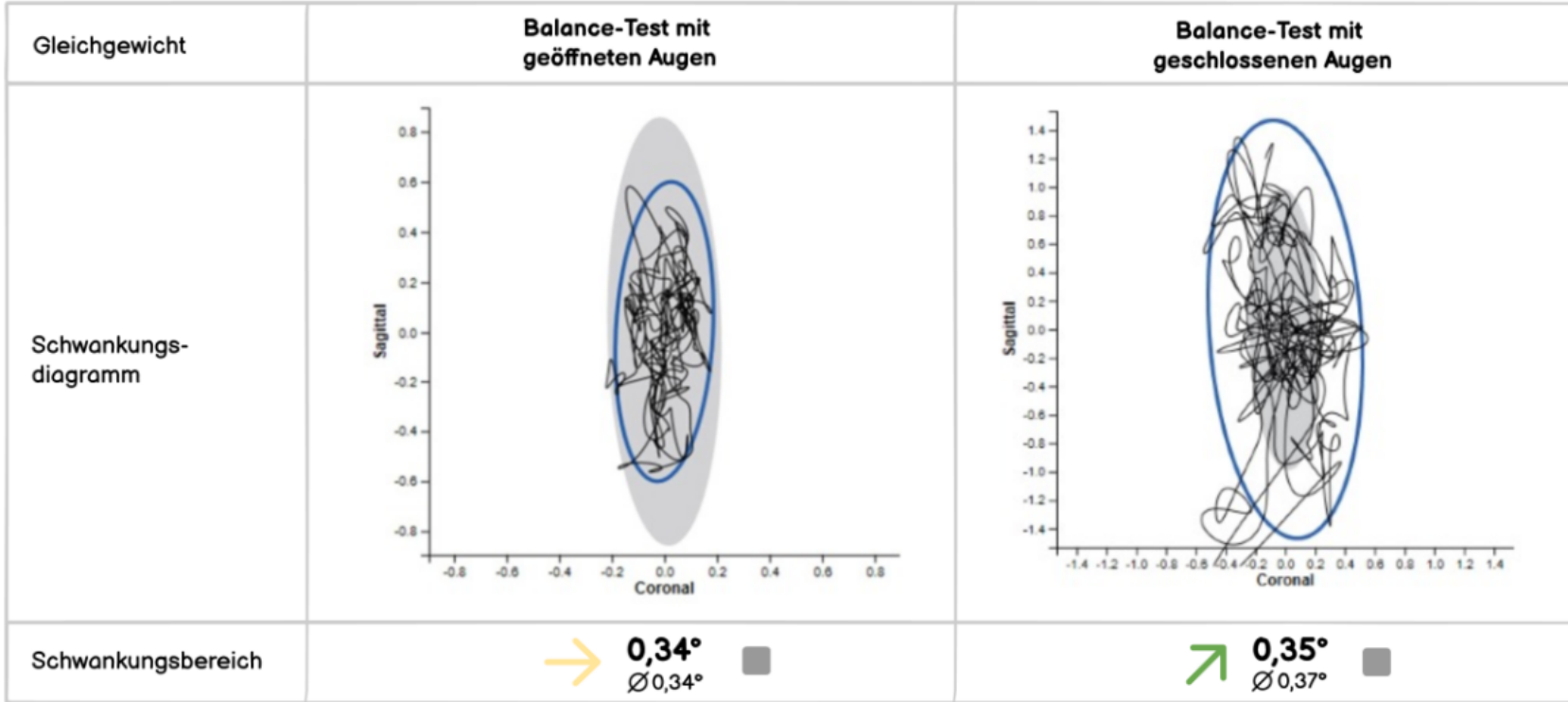

</td></tr>
<tr><td>
Include the corresponding test for each critical value in the "Critical Values" section.

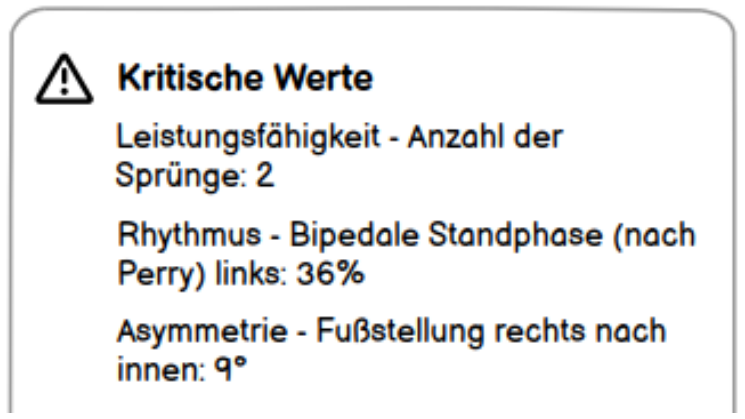

.
</td><td>
The corresponding test was added for each critical value (in this case, "Normal Walking" - highlighted with a green circle in the figure).

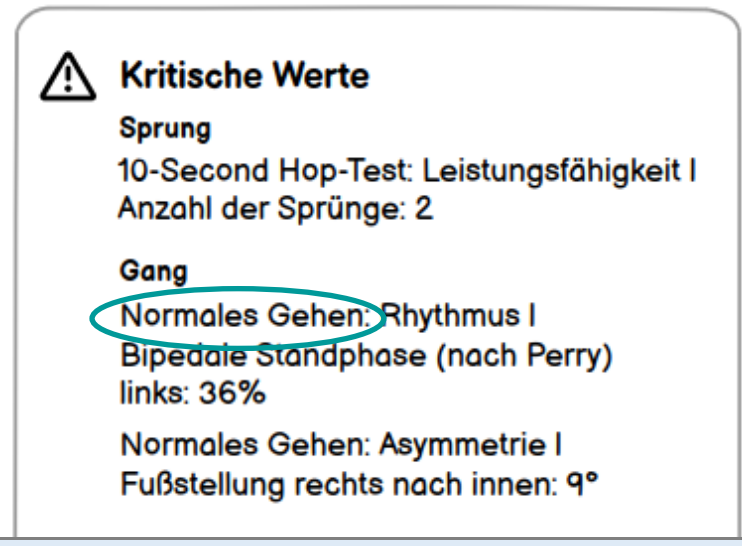

</td></tr>
<tr><td>Detailed test view</td><td></td></tr>
<tr><td>
Add a section heading above the gait parameters in the detailed view for all tests.

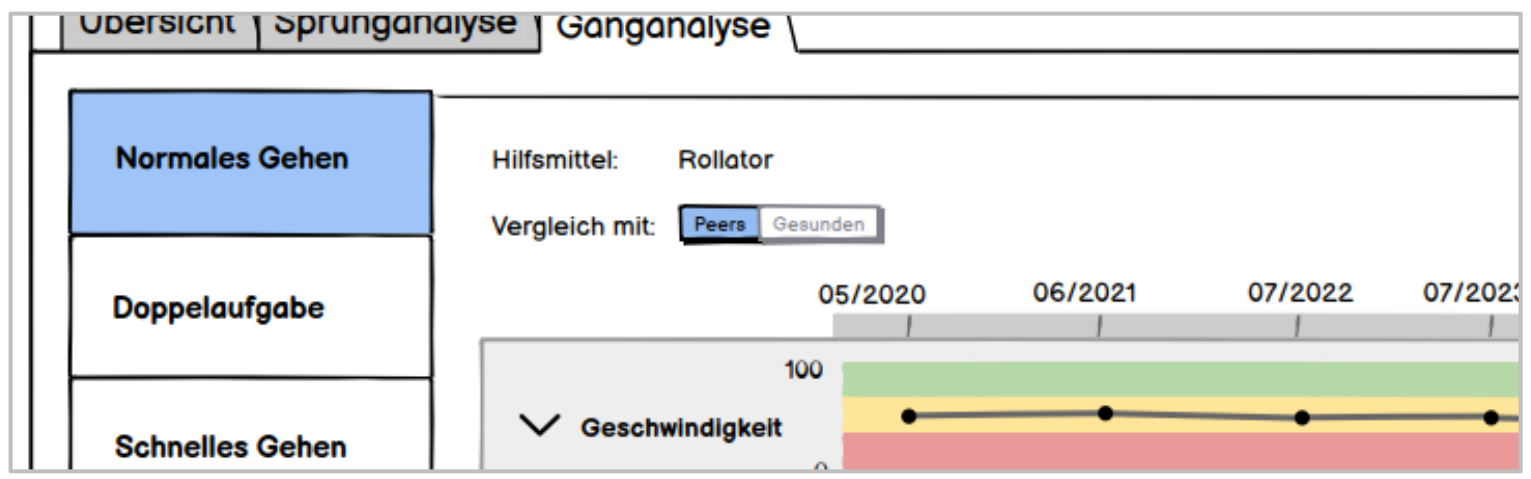

</td><td>
The heading was added to the detailed view for all tests (highlighted with a green circle in the figure).

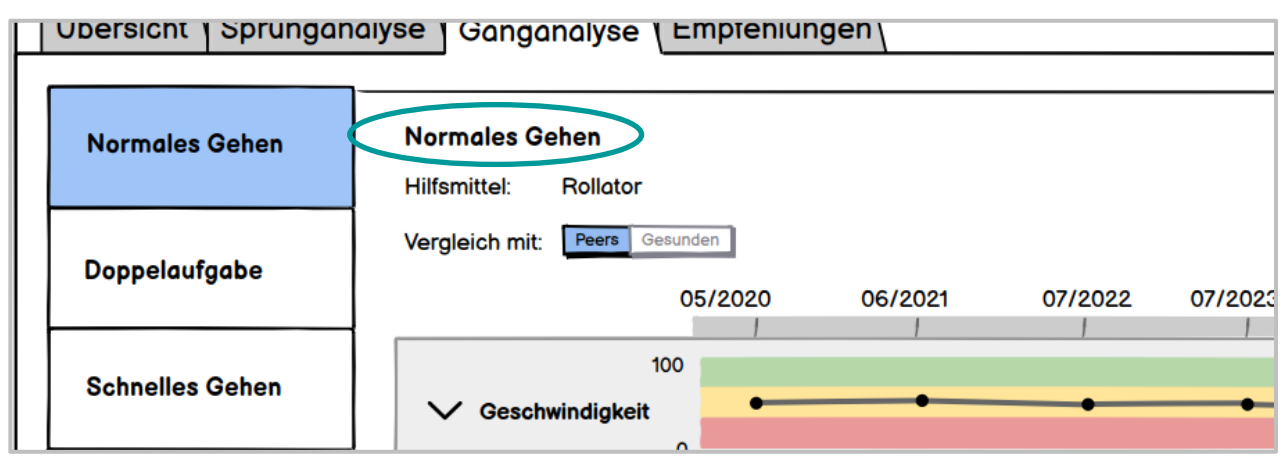

</td></tr>
</table>

<table>
<tr><th>Suggestions / Key findings (Prototype V1)</th><th>Change made (Prototype V2)</th></tr>
<tr><td>Reference range limits do not need to be displayed numerically; however, the display range limits should be indicated.
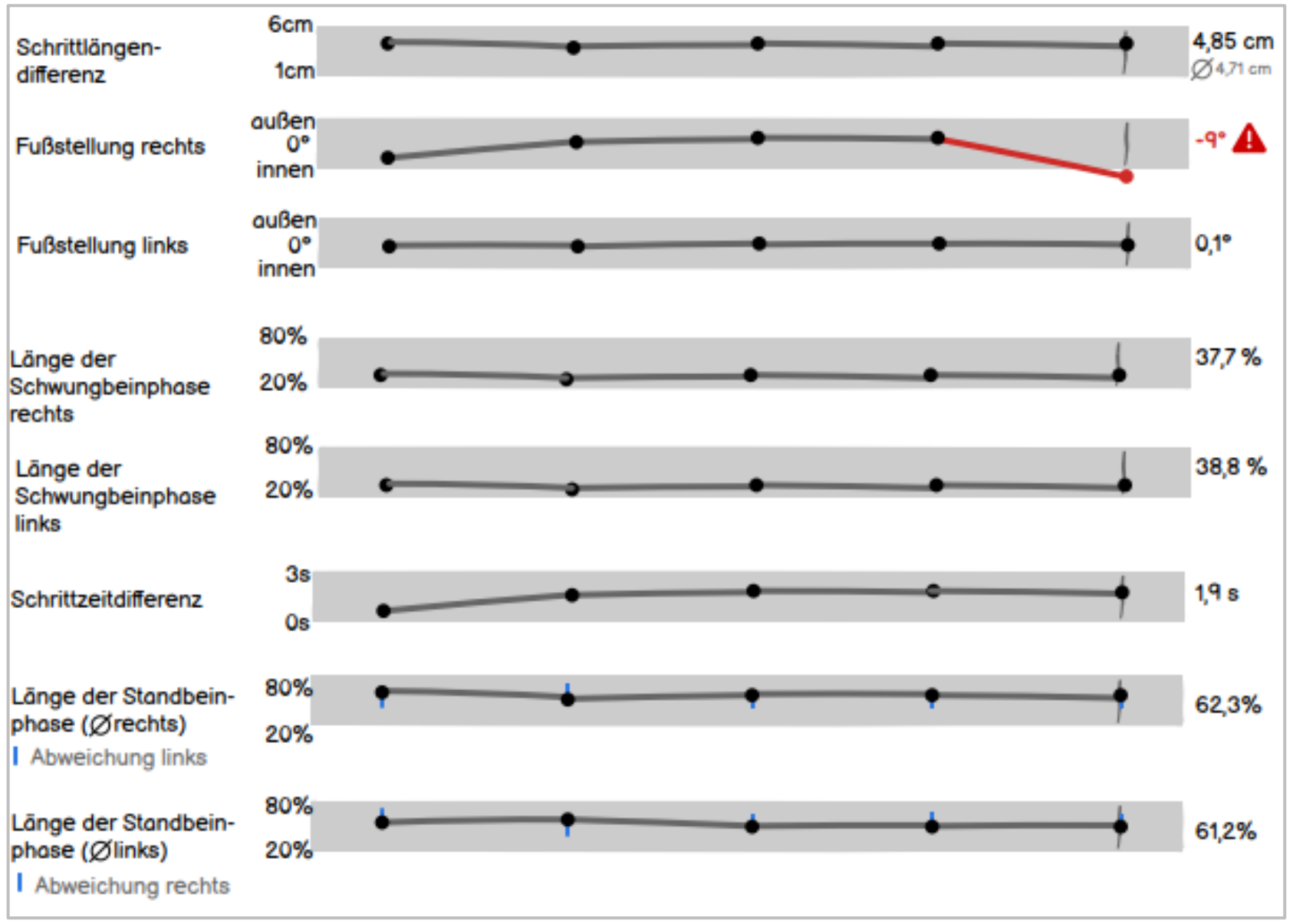
</td><td>Boundary lines for the upper and lower limits of the display range were added. The gray reference range is displayed within the display range. (Indicated by a green circle in the figure.)
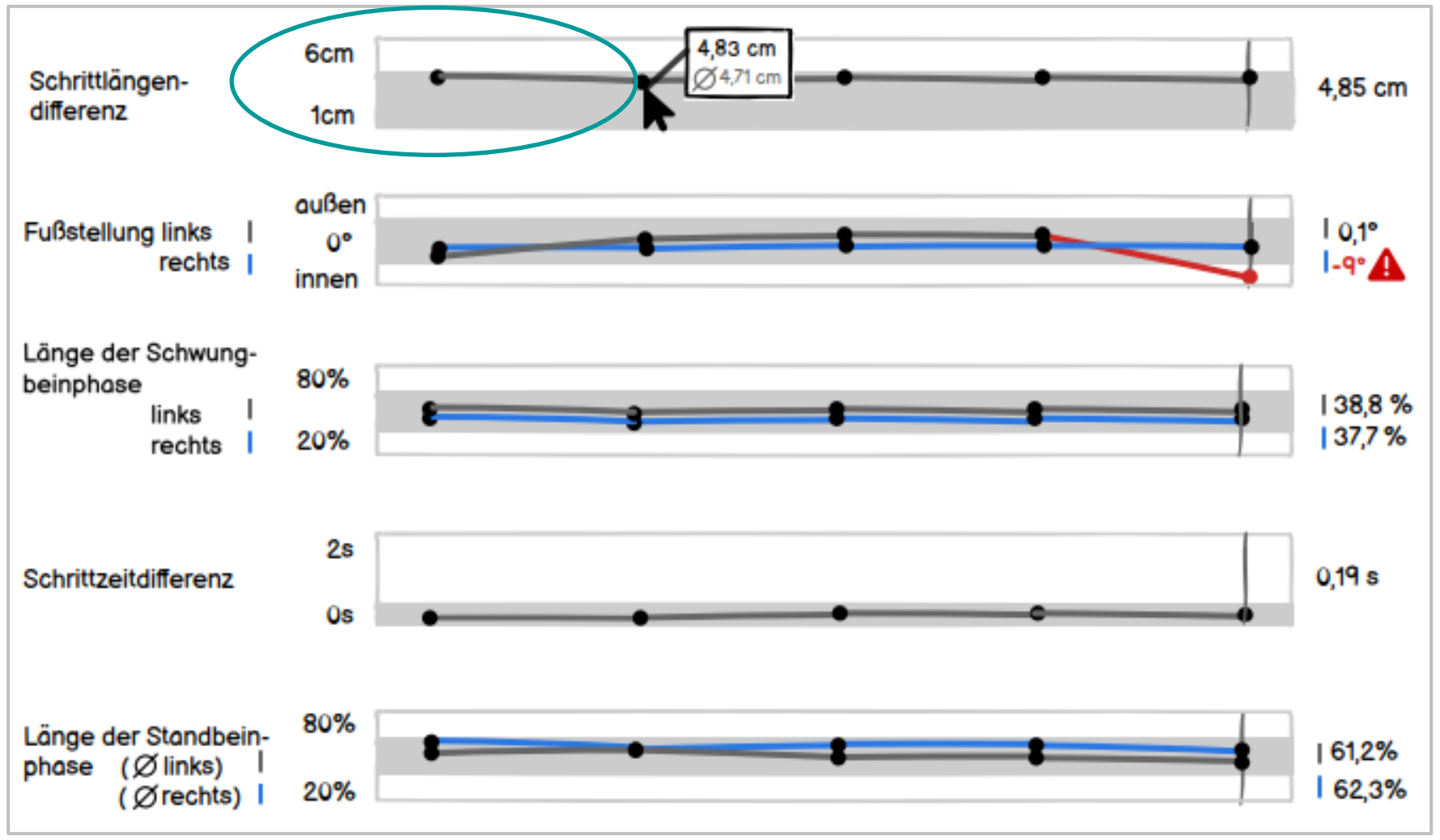
</td></tr>
<tr><td>The average value does not need to be displayed directly; displaying it in the mouseover is sufficient.
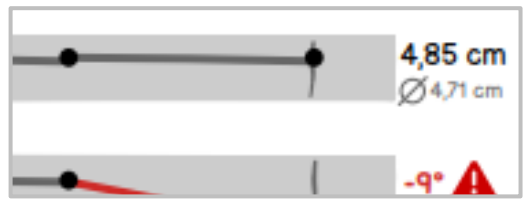
</td><td>The average value is now displayed only in the mouseover and is no longer shown directly on the right side of the trend display.
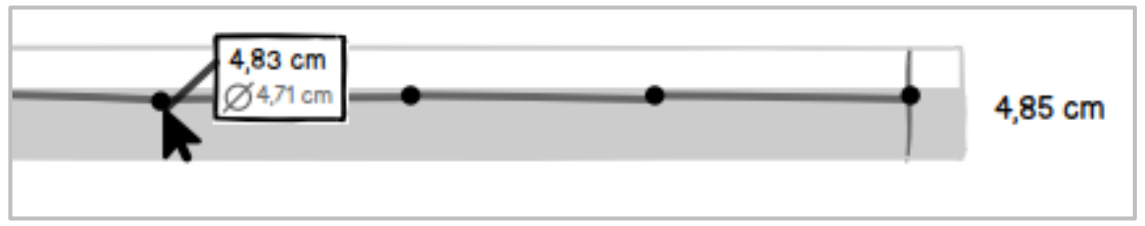
</td></tr>
<tr><td>Since the left and right sides, including the respective deviations between sides, are displayed in two separate charts, the page becomes unnecessarily long vertically. Values collected for both sides can be combined into a single chart.
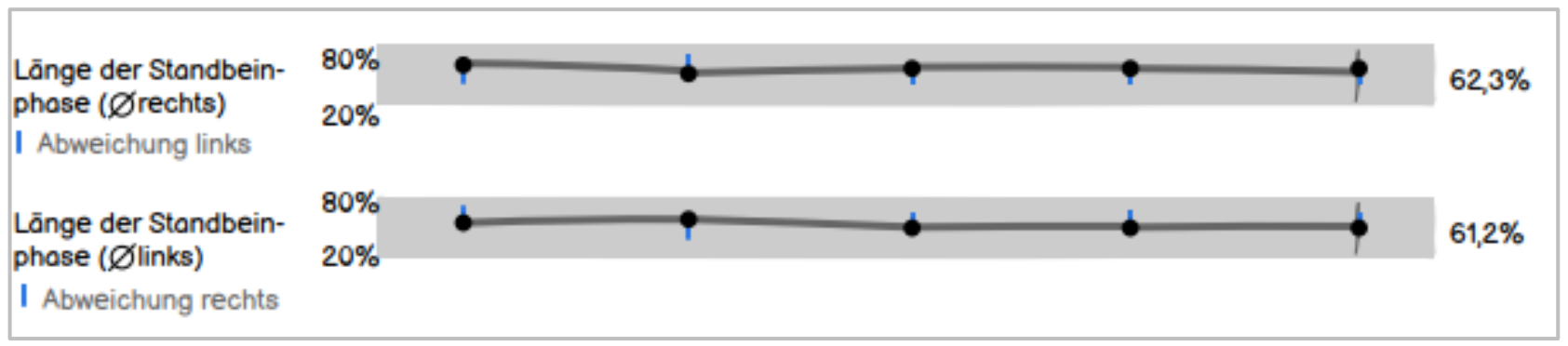
</td><td>Values collected for both sides are now displayed in a single chart (left side in gray and right side in blue).
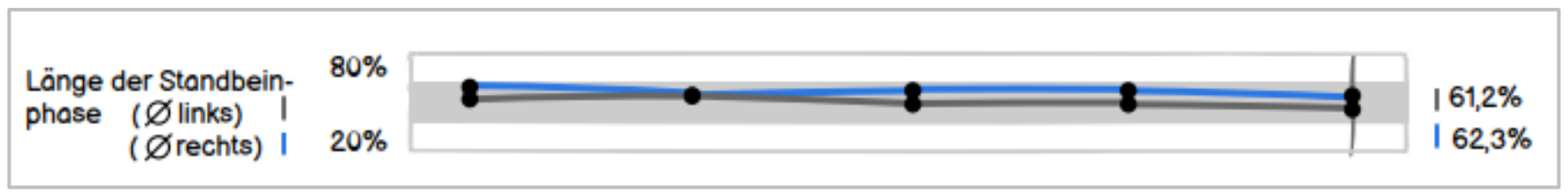
</td></tr>
</table>

**Suggestions / Key findings** (Prototype V1)	**Change made** (Prototype V2)
Therapeutic recommendations should not be displayed on every individual view.<br>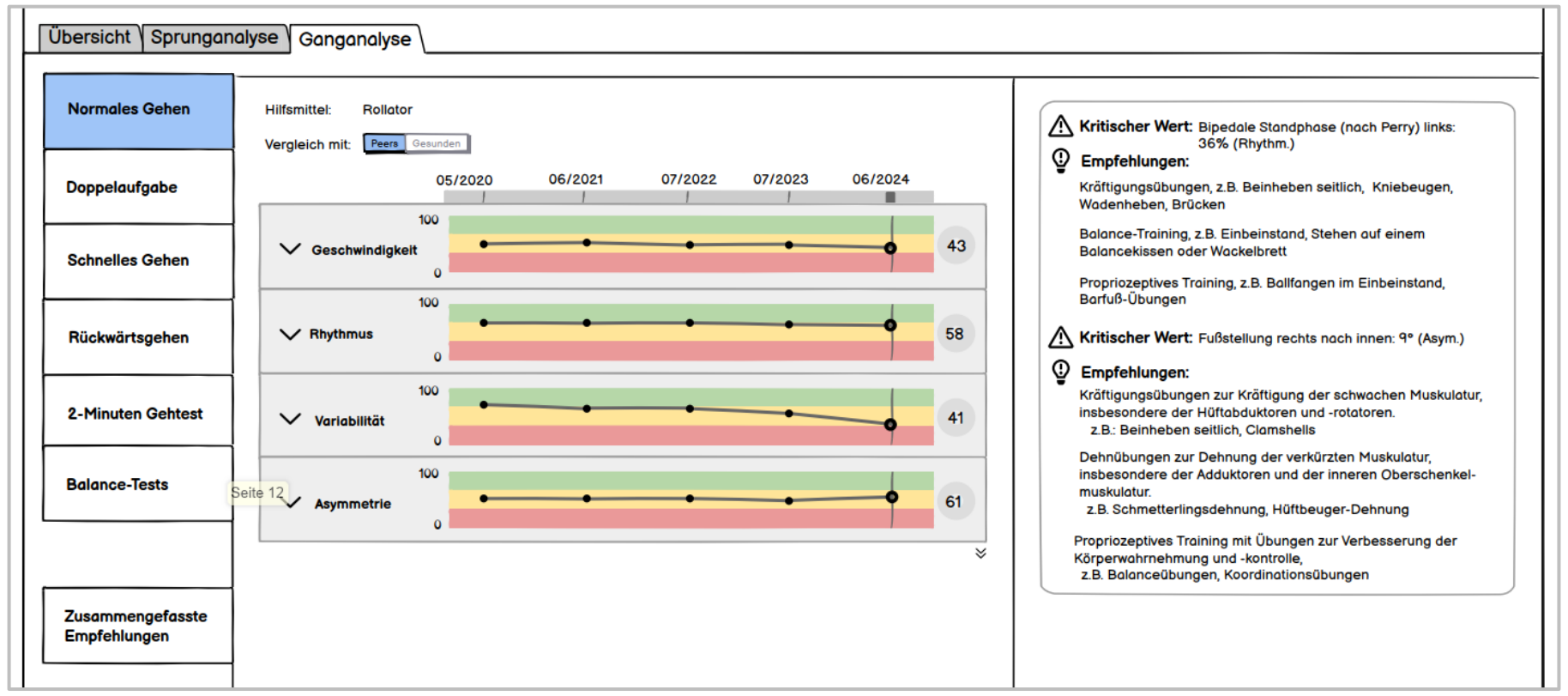	Therapeutic recommendations were removed from the individual test-specific views.<br>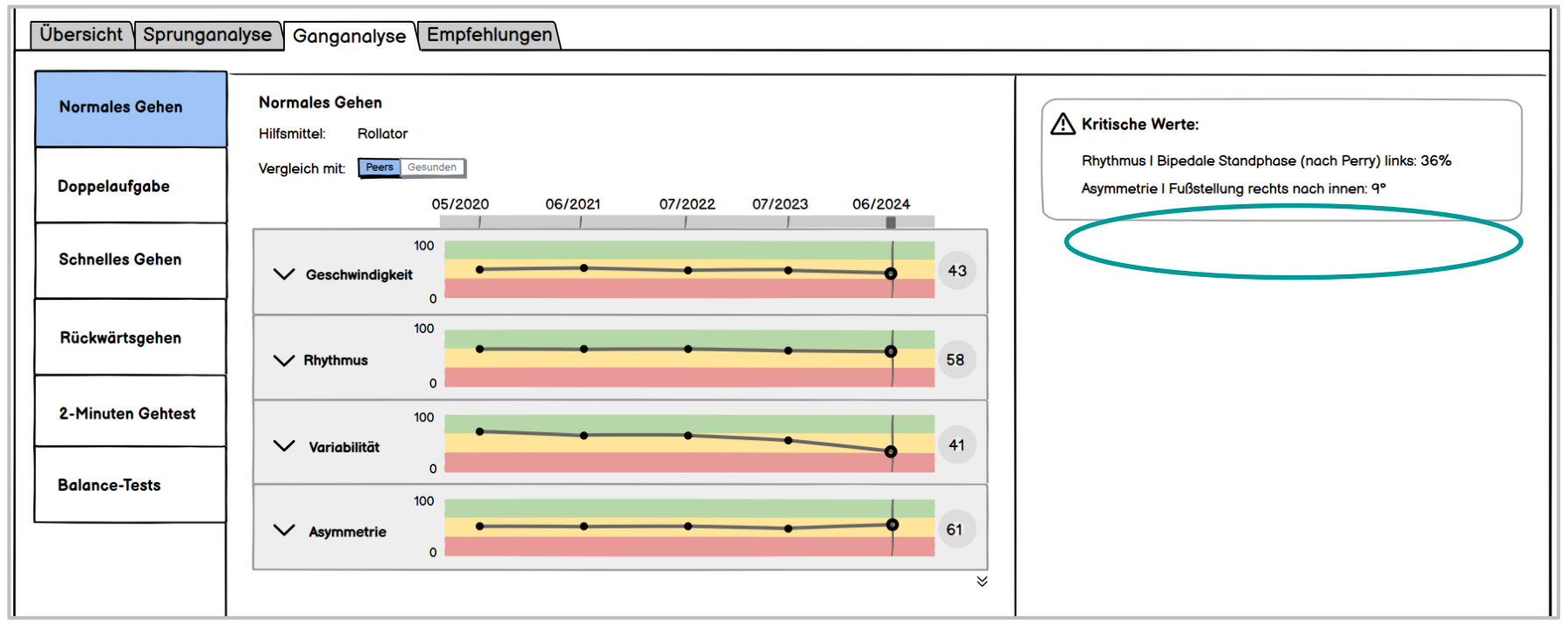
It is not necessary to divide recommendations by individual tests, as this would likely result in redundant information. Instead, recommendations should be grouped by higher-level categories (e.g., gait, balance).<br>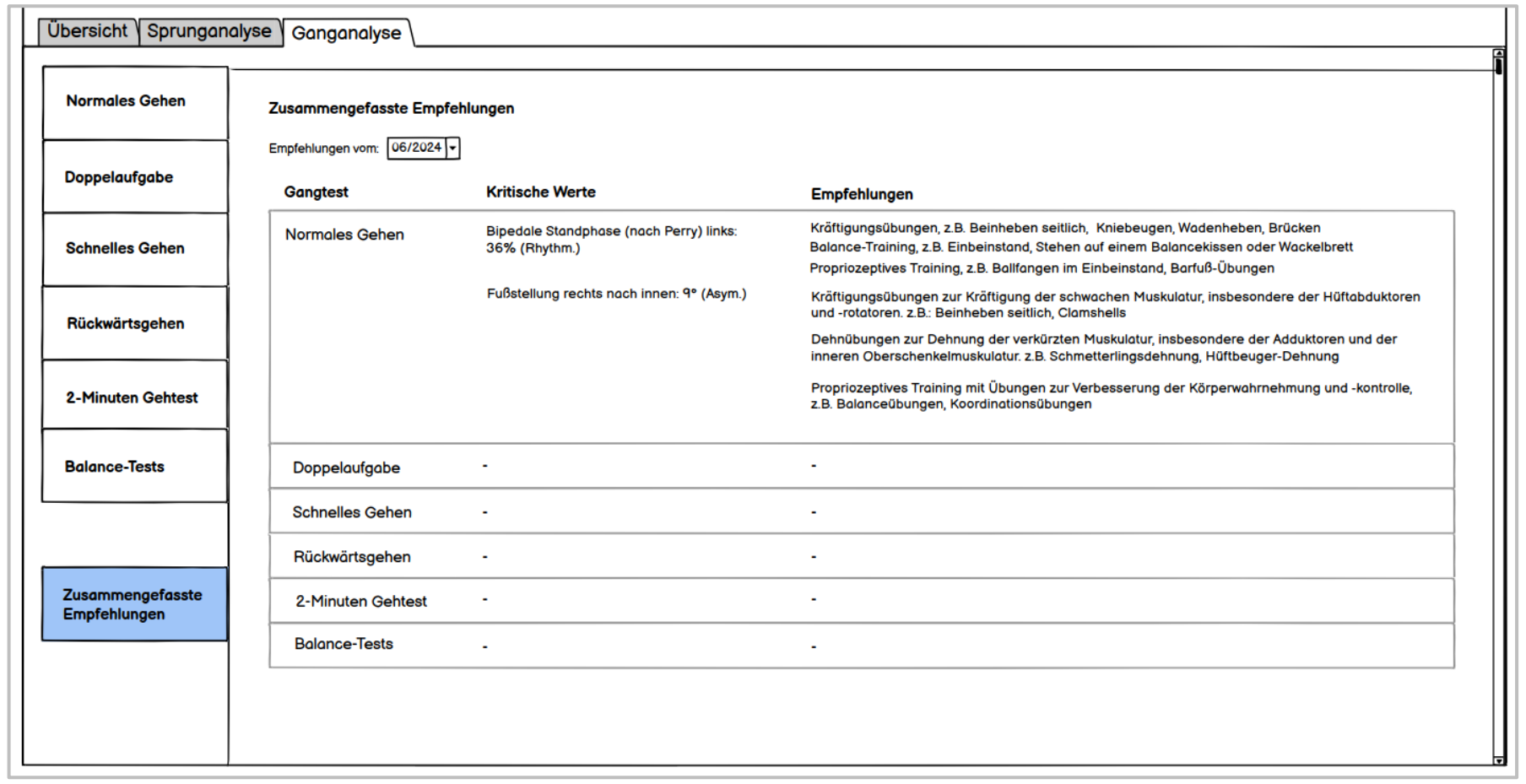	Recommendations were moved to a new tab in the top navigation and are now displayed according to higher-level categories.<br>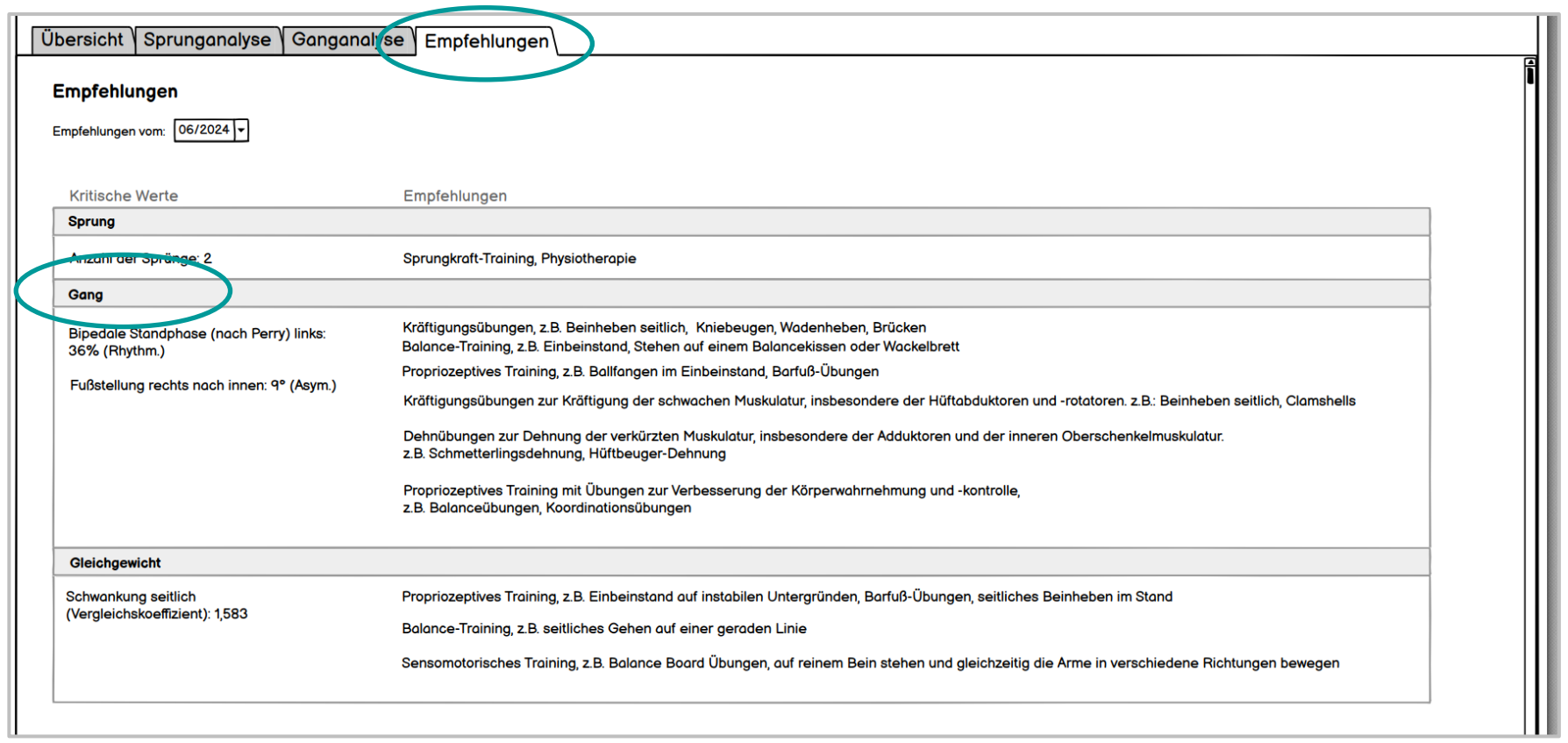
Parameters should additionally be visualized using pictograms, particularly to improve comprehensibility for patients (lower priority).	As this suggestion was considered a lower priority, the implementation was deferred to a later high-fidelity version. Since the development of suitable pictograms requires careful consideration, it was considered beyond the scope of the current prototype iteration.
Add a gauge visualization to the scores displayed at the end of the charts.	This suggestion was deferred, as the design team assessed that this type of visualization would not provide a significant additional information benefit.

<table>
<tr><th>Suggestions / Key findings (Prototype V1)</th><th>Change made (Prototype V2)</th></tr>
<tr><td colspan="2">Balance section</td></tr>
<tr><td>The charts should be displayed side by side.

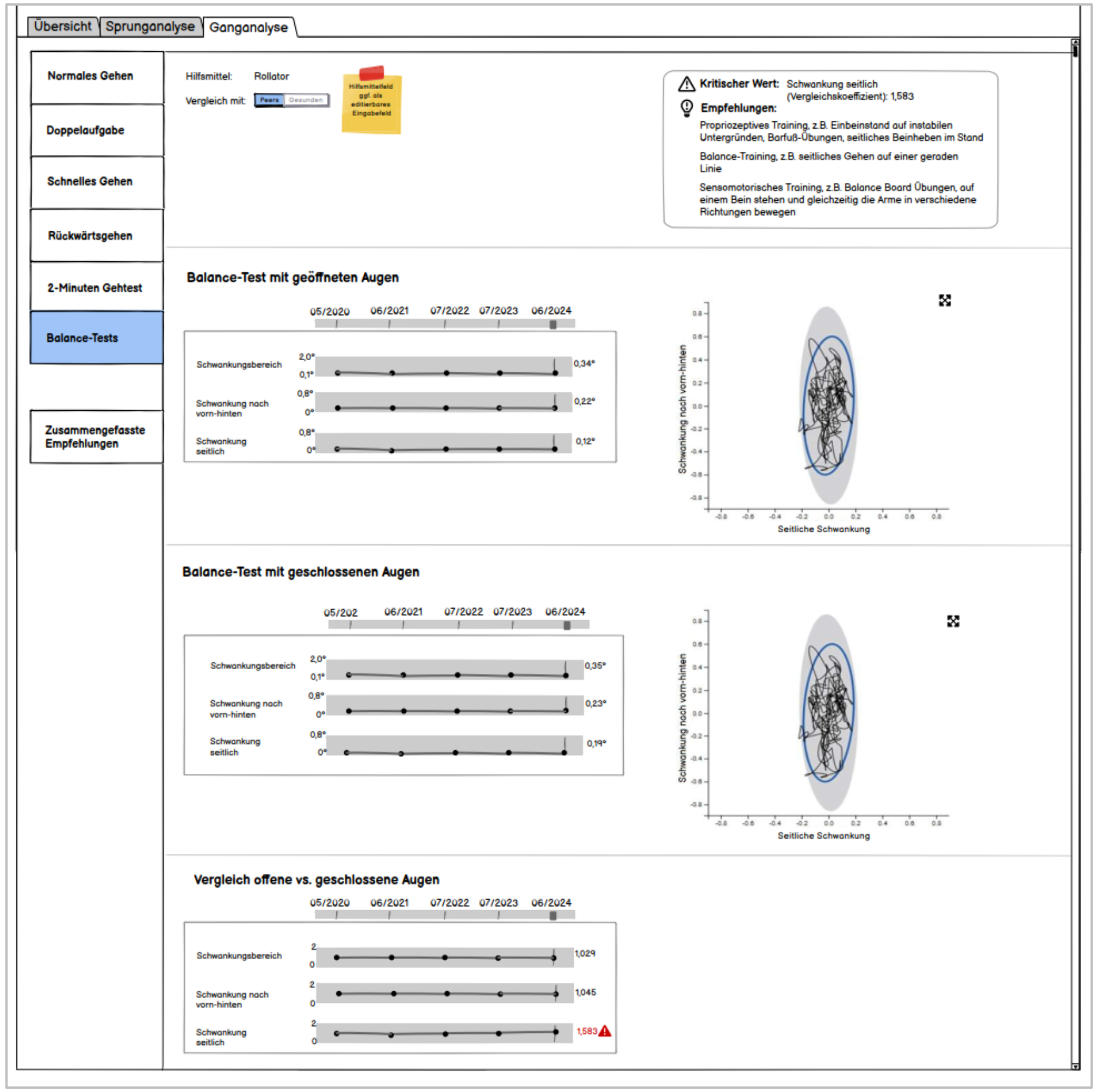
</td>
<td>The charts were arranged side by side instead of being displayed vertically.

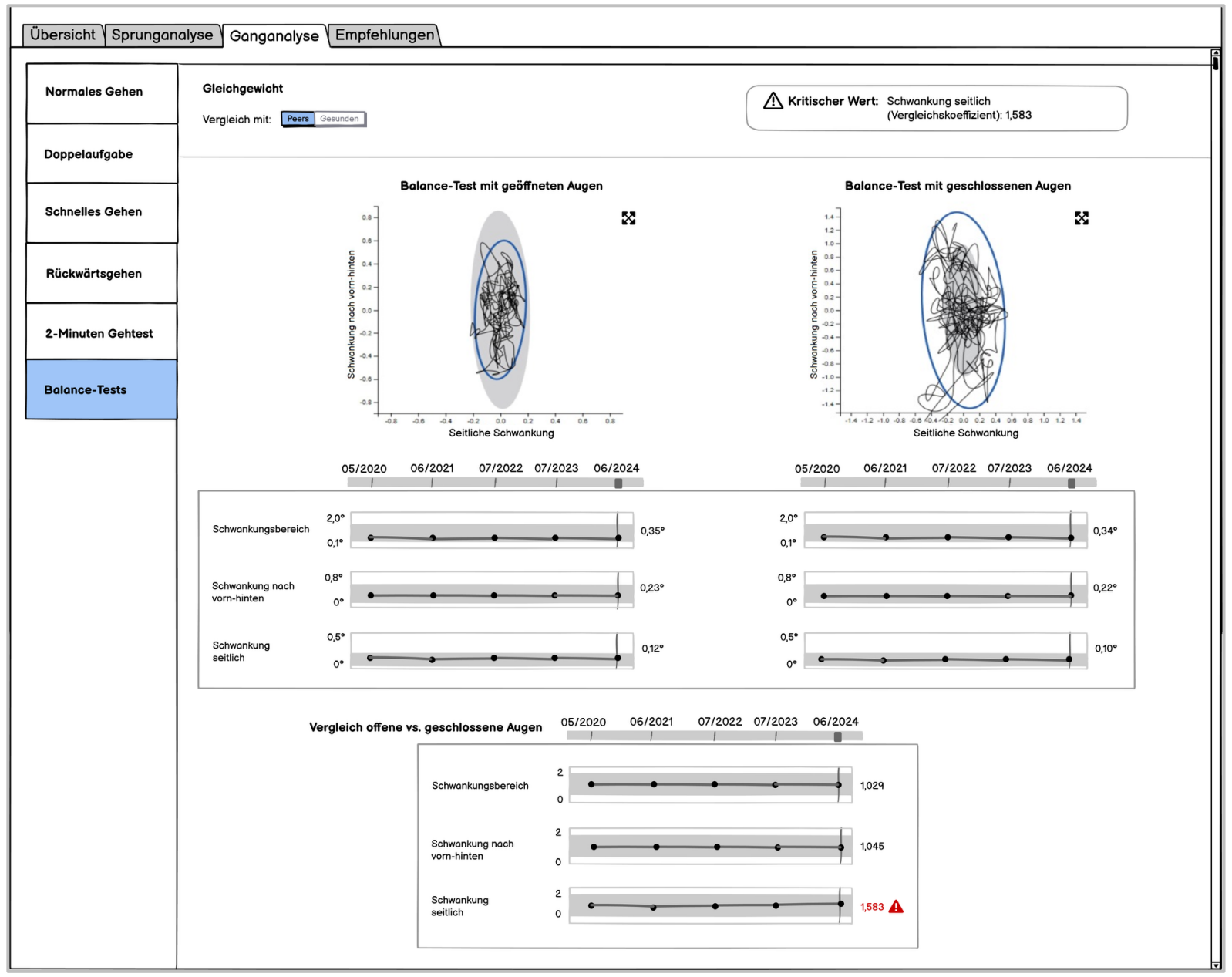
</td></tr>
</table>

**Suggestions / Key findings** (Prototype V1)	**Change made** (Prototype V2)
Balance tests are performed while standing, without the use of assistive devices. 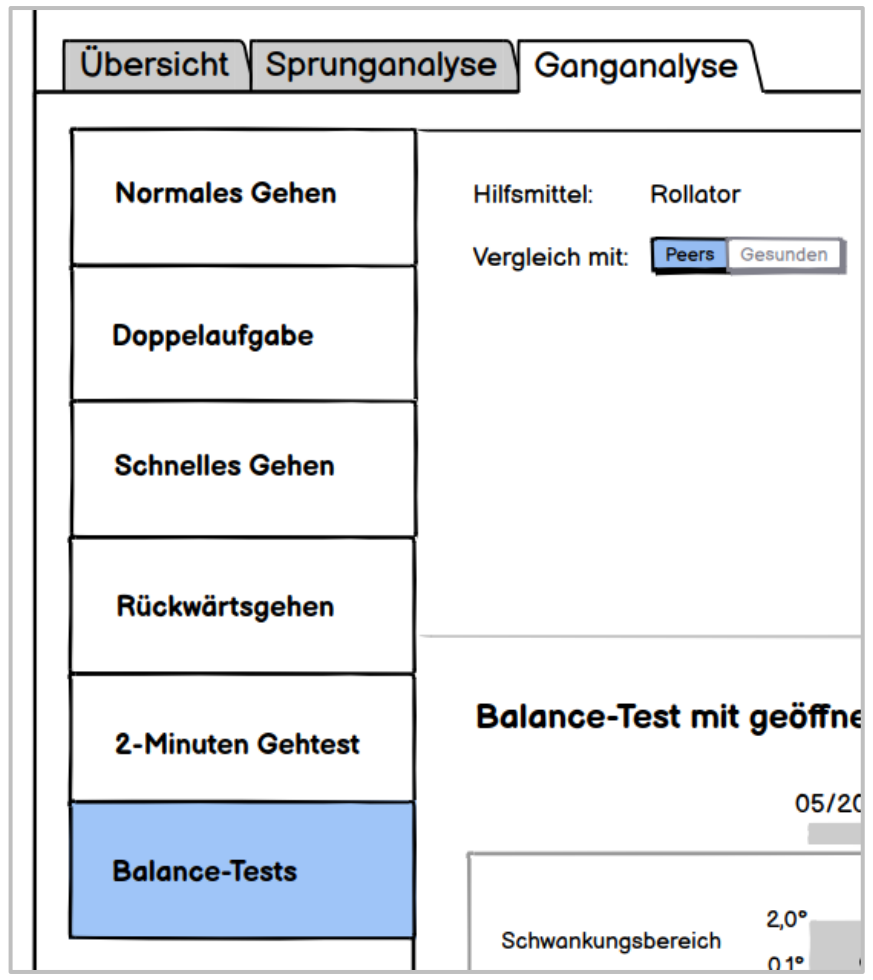	The "Assistive Devices" information was removed from the balance test views. 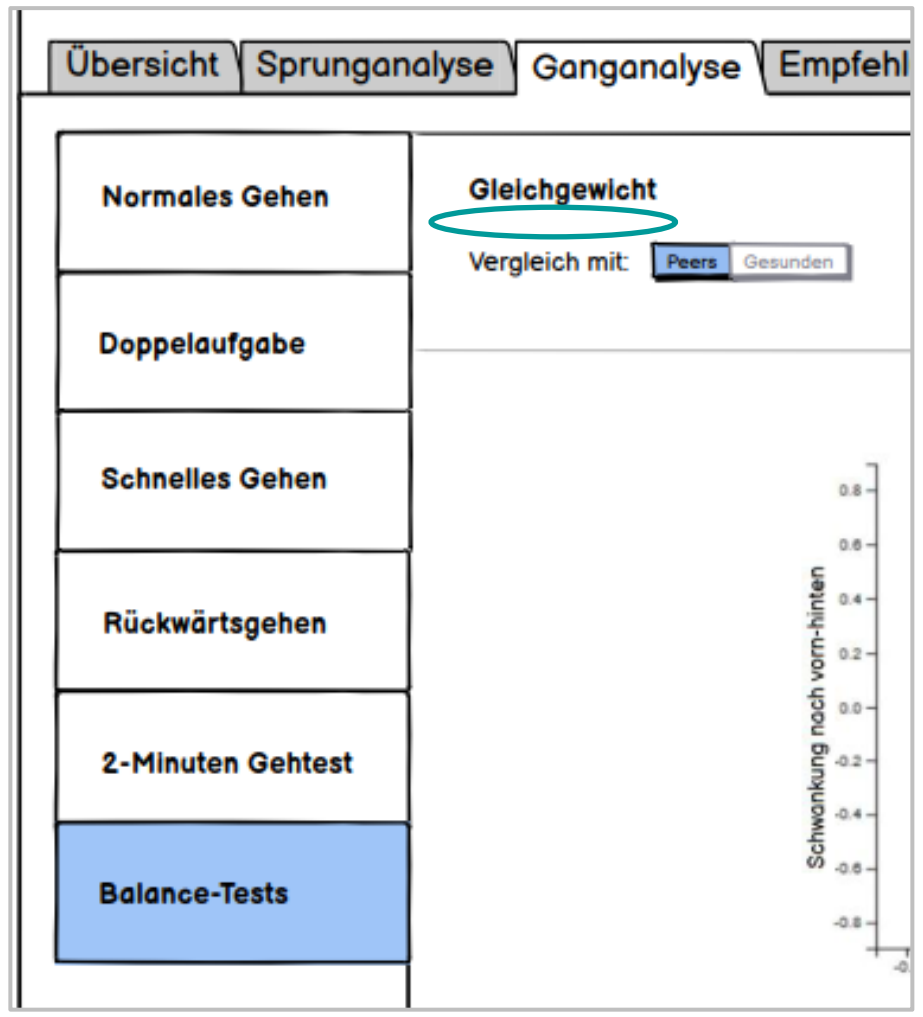
