# Supplementary material for: User-centered design and formative evaluation of a gait dashboard for multidimensional walking assessment in multiple sclerosis
Source: Front Digit Health. 2026 Sep 8;8:1865736. doi: 10.3389/fdgth.2026.1865736 (PMC13593768; doi:10.3389/fdgth.2026.1865736)
Supplement: Supplementary file 3 [file Datasheet3.pdf]

Questionnaire ID:

Patient Questionnaire

Evaluation of newly developed screen visualizations to support the discussion of gait analysis results

Dear Patient,

As part of your appointment with us, you will undergo several examinations, including a gait analysis. At the end of this test, we will discuss your results with you and show you the corresponding visualizations on a computer screen.

For these result visualizations, we have developed new concepts designed to make the discussion even clearer and more helpful.
With this survey, we would like to find out how well you understand the new visualizations and whether they provide added value for you.

We would greatly appreciate it if you could answer the following questions.
Your feedback will help us further improve the presentation of results.

Estimated duration: approx. 20 minutes
Your responses are anonymous and will, of course, be treated confidentially.

If you have any questions about the survey, please feel free to contact our study team at the Mobility Center at any time.

Thank you very much for your support!

Please place the completed questionnaire in the box next to the gait analysis laboratory **(Room 3.034). Thank you!**

Topic: Gait Analysis

Questions about the Presentation of Gait Analysis Results

Please answer these questions only if a gait analysis has already been carried out with you (during this or a previous appointment).

Question 1. The results of the gait analysis are shown to you by our study staff on a computer screen. The current display looks as follows: (see attached views 1 and 2).

To make this display easier to understand, we have developed a concept for a new presentation. Please watch the approximately 5-minute video on the PC: (see Video – Gait Analysis).

Question 1.1: How satisfied are you with the concept (see video) of the new presentation of the results on the computer screen?

	Not at all true	Partly true	Completely true	Don't know
**Detectability**<br>The displayed information is easy for me to recognize. It is presented in a way that I can easily perceive it.	☐	☐	☐	☐
**Legibility**<br>The information is displayed in a sufficiently large font size. I can clearly distinguish the text from the background (contrast between text and background).	☐	☐	☐	☐
**Freedom from distraction**<br>The displayed information is, in my opinion, free from distraction. I can perceive all required information without being disturbed by additional elements.	☐	☐	☐	☐
**Distinguishability**<br>I can easily distinguish the displayed information from other elements or groups of elements.	☐	☐	☐	☐
**Clear interpretability / Understandability**<br>The displayed information is clearly interpretable and easy to understand for me, even without additional explanations from the medical staff.	☐	☐	☐	☐
**Compactness / Amount of information**<br>The information display is compact. In my opinion, only the information relevant for discussing results is shown.	☐	☐	☐	☐

Topic: Gait Analysis

	Not at all true	Partly true	Completely true	Don’t know
**Consistency**<br>The displayed information is consistent: information elements with similar intent are designed uniformly, while elements with different intents are clearly distinguished in style and form.	☐	☐	☐	☐
**Color design**<br>I am satisfied with the color representation of the values.	☐	☐	☐	☐
**Aesthetics**<br>I find the information display visually appealing.	☐	☐	☐	☐
**Terminology**<br>The labels of the values are easy for me to understand.	☐	☐	☐	☐
**Overall satisfaction**<br>Overall, I am satisfied with the display of the results on the computer screen as currently shown for the joint discussion of my gait analysis evaluation.	☐	☐	☐	☐

Question 1.2: Is there anything **you miss in the new presentation (video) or that you would improve**? Your comments:

Topic: Gait Analysis

Question 2: Please evaluate the new presentation (video) compared with the previous display (attached view) on the computer screen.

Question 2.1 Usability & Orientation

How easy is it for you to find your way around the new presentation compared to the old one?

- ☐ Much easier than before
- ☐ Somewhat easier than before
- ☐ No difference to the old presentation
- ☐ Somewhat more difficult than before
- ☐ Much more difficult than before

Question 2.2 Intuitiveness

How intuitive does the new presentation appear to you compared to the old one?

- ☐ Much more intuitive – I understand it immediately
- ☐ Somewhat more intuitive – understandable with a bit of thought
- ☐ No difference from the old presentation
- ☐ Somewhat less clear – I need explanations
- ☐ Much less clear – I do not understand it without explanations

Question 2.3 Support during consultations

Would the new presentation help you better understand the results during the discussion with the study staff?

- ☐ Yes, much better
- ☐ Yes, somewhat better
- ☐ No, no difference
- ☐ No, somewhat worse
- ☐ No, much worse

Would the new presentation help you better understand your own health situation?

- ☐ Yes, much better
- ☐ Yes, somewhat better
- ☐ No, no difference
- ☐ No, somewhat worse
- ☐ No, much worse

Would the new presentation help you better derive motivation and actions (e.g., regular exercises) for your daily life?

- ☐ Yes, much better
- ☐ Yes, somewhat better
- ☐ No, no difference
- ☐ No, somewhat worse
- ☐ No, much worse

Question 2.4 Preference

Welche Darstellung bevorzugen Sie insgesamt?

- ☐ The new presentation (video)
- ☐ The old presentation (attached view)
- ☐ No clear preference

Personal Information

Questions About You

Answering these questions is voluntary. However, your information will help us better interpret your previous responses in the questionnaire

Question 1: Your gender?

- ☐ Male
- ☐ Female

Question 2: Your age?

- ☐ Younger than 18 years
- ☐ 18–25 years
- ☐ 26–35 years
- ☐ 36–45 years
- ☐ 46–55 years
- ☐ 56–65 years
- ☐ 66–75 years
- ☐ 76–85 years
- ☐ Older than 85 years

Question 3: What is your highest level of completed education?

- ☐ No vocational or academic training started
- ☐ Currently in vocational or academic training
- ☐ No vocational or academic qualification completed
- ☐ Completed vocational training
- ☐ Master craftsman / technician qualification or degree from a vocational or professional academy
- ☐ Academic degree
- ☐ Other educational qualification

Question 4: In which year were you diagnosed with multiple sclerosis (MS)?

Year of initial MS diagnosis: ________________ (please specify the year)

Question 5: How many gait analysis appointments have you already completed?

- ☐ None
- ☐ 1–2
- ☐ 3–5
- ☐ 6–10
- ☐ More than 10

Personal Information

Question 6: How would you rate your current state of health?

- ☐ Very good
- ☐ Good
- ☐ Satisfactory
- ☐ Poor
- ☐ Very poor

Question 7: How would you rate your computer skills/knowledge?

- ☐ Low – I find many systems difficult to use.
- ☐ Moderate – I manage well with most systems.
- ☐ High – I am very experienced and technically proficient.

Question 8: How would you assess your current stage of illness?

Please read the statements below and choose the one that best describes your situation. The following focuses primarily on how well you are able to walk. You may not find a description that fits your condition exactly. However, please check the one category that best represents your situation.

- ☐ **0 – Normal**
  It may happen that, due to my MS, I have mild symptoms, mainly sensory (such as altered sensations), but they do not limit my activities. When I have a relapse, my condition returns to normal after the relapse has passed.

- ☐ **1 – Mild disability**
  I have some noticeable symptoms due to my MS, but they are minor and have little impact on my lifestyle.

- ☐ **2 – Moderate disability**
  My ability to walk is not impaired. However, I have significant problems due to my MS that limit me in performing my daily activities in other ways.

- ☐ **3 – Walking impairment**
  MS affects my activities, particularly walking. I can work a full day, but sports or physically demanding activities are more difficult than before. Normally, I do not need a walking stick or other aid, but I may need some assistance during a relapse.

- ☐ **4 – Occasional use of a walking aid**
  I use a walking stick, crutch, or other form of support (e.g., holding onto a wall or another person’s arm) regularly or occasionally, especially when walking outdoors. I believe I can walk 10 meters in 20 seconds without a walking stick or crutch. However, I always need some assistance (walking stick or crutch) when I want to walk longer distances.

- ☐ **5 – Constant use of a walking aid**
  To walk 10 meters, I need a walking stick, crutch, or someone to hold on to.
  I can move around the house or in other buildings by holding onto furniture or supporting myself against walls. When I want to cover longer distances, I may occasionally use a wheelchair.

- ☐ **6 – Bilateral support**
  To be able to walk 10 meters, I must use two crutches or a walker. For longer distances, I may need to use a wheelchair.

Personal Information

☐ **7 – Wheelchair**
My main means of mobility is a wheelchair. I may be able to stand and/or take one or two steps, but I cannot walk 10 meters even with crutches or a walker.

☐ **8 – Bedridden**
I am unable to sit in a wheelchair for more than one hour.

A questionnaire cannot always address every aspect. Here is space for your individual comments. Would you like to draw our attention to anything else?

Thank you very much for your participation! - We would like to sincerely thank you for your valuable time and for supporting our work.

Please place the completed questionnaire in the box next to the gait analysis laboratory (Room 3.034).

Gait parameters of the walking tests

Gait analysis – View 1

Functional Ambulation Profile: **90**

Bilateral Parameters	*Left*	*Right*
Step Time (sec)	,60/5,5	,58/4,5
Cycle Time (sec)	1,18/2,6	1,17/4,4
Step Length (cm)	67,08/4,5	62,23/4,4
Stride Length (cm)	129,31/3,2	129,85/3,7
H-H Base Support (cm)	18,80	18,77
Single Support (%GC)	37,5	38,9
Double Support (%GC)	24,2	24,5
Swing (%GC)	38,8	37,7
Stance (%GC)	61,2	62,3
Step/Extremity Ratio	,64	,59
Toe In / Out (deg)	5	4

Parameters	
Distance (cm)	1360,2
Ambulation Time (sec)	12,34
Velocity (cm/sec)	110,2
Mean Normalized Velocity	1,05
Number of Steps	21
Cadence (Steps/Min)	102,1
Step Time Differential (sec)	,02
Step Length Differential (cm)	4,85
Cycle Time Differential (sec)	,00

FAP Deduction From	100
Step Functions Left	-2
Step Functions Right	-4
Diff Step Extr. Ratio	-1
HH Dyn. Base of Supp	-3
Ambulatory Aids	
Assistive Devices	
FAP Score	90

Balance parameters

Gait analysis – View 2

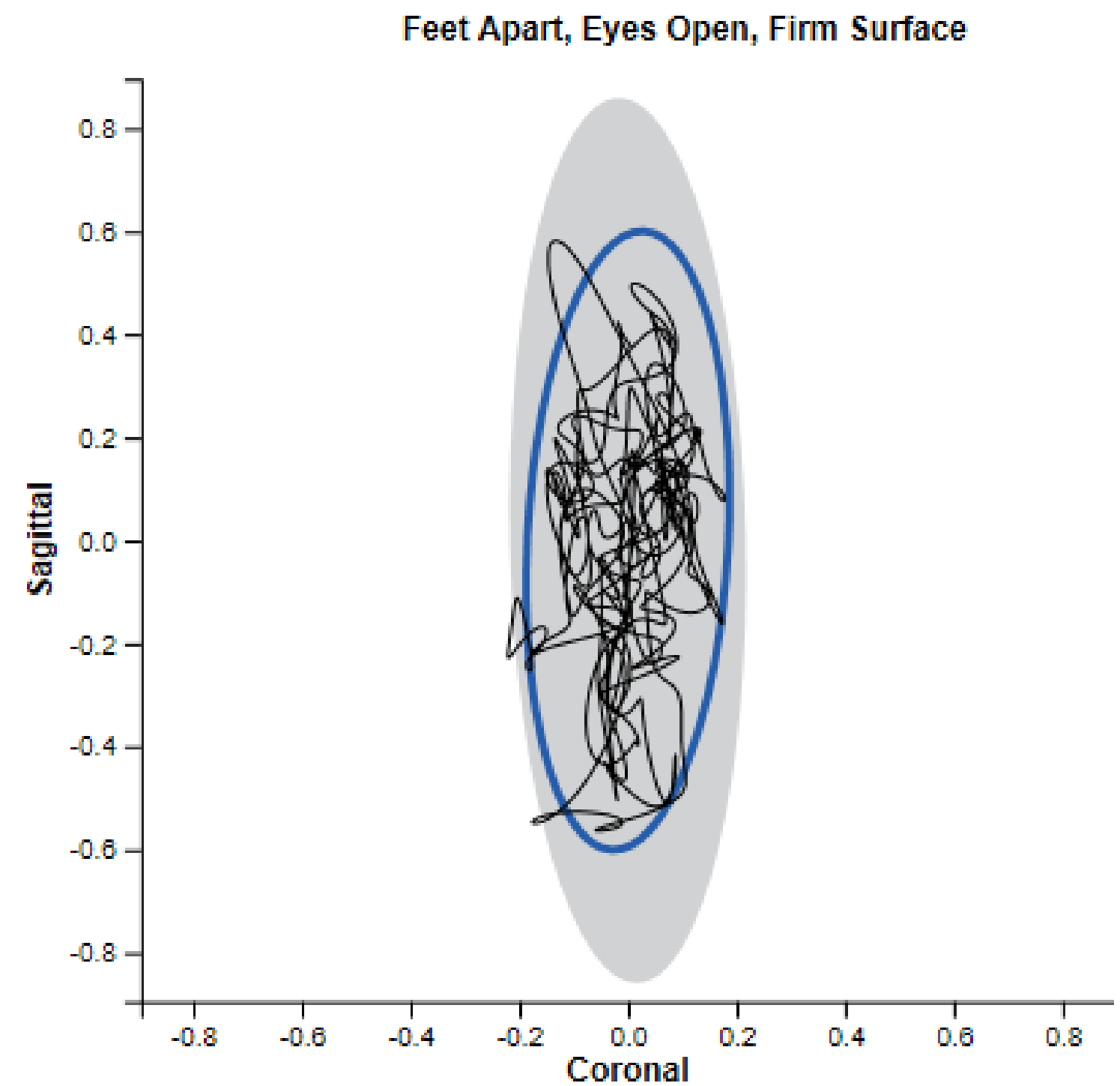

Overview Metric Values

Postural Sway - Angles

UNILATERAL	NORMATIVE	2024-05-14 15:36	CHANGE FROM 2022-10-04 16:22
Duration(s)	--	30.0	↓ 0.0%
Sway Area(°²)	0.162 — 1.40	0.350	↑ 124.1%
RMS Sway(°)	0.180 — 0.655	0.246	↑ 40.7%
RMS Sway (Coronal)(°)	0.0501 — 0.177	0.0806	↑ 62.8%
RMS Sway (Sagittal)(°)	0.165 — 0.615	0.233	↑ 38.6%
