# Supplementary material for: User-centered design and formative evaluation of a gait dashboard for multidimensional walking assessment in multiple sclerosis
Source: Front Digit Health. 2026 Sep 8;8:1865736. doi: 10.3389/fdgth.2026.1865736 (PMC13593768; doi:10.3389/fdgth.2026.1865736)
Supplement: Supplementary file 4 [file Datasheet4.pdf]

Tanja Marek

Rater at the Mobility Center

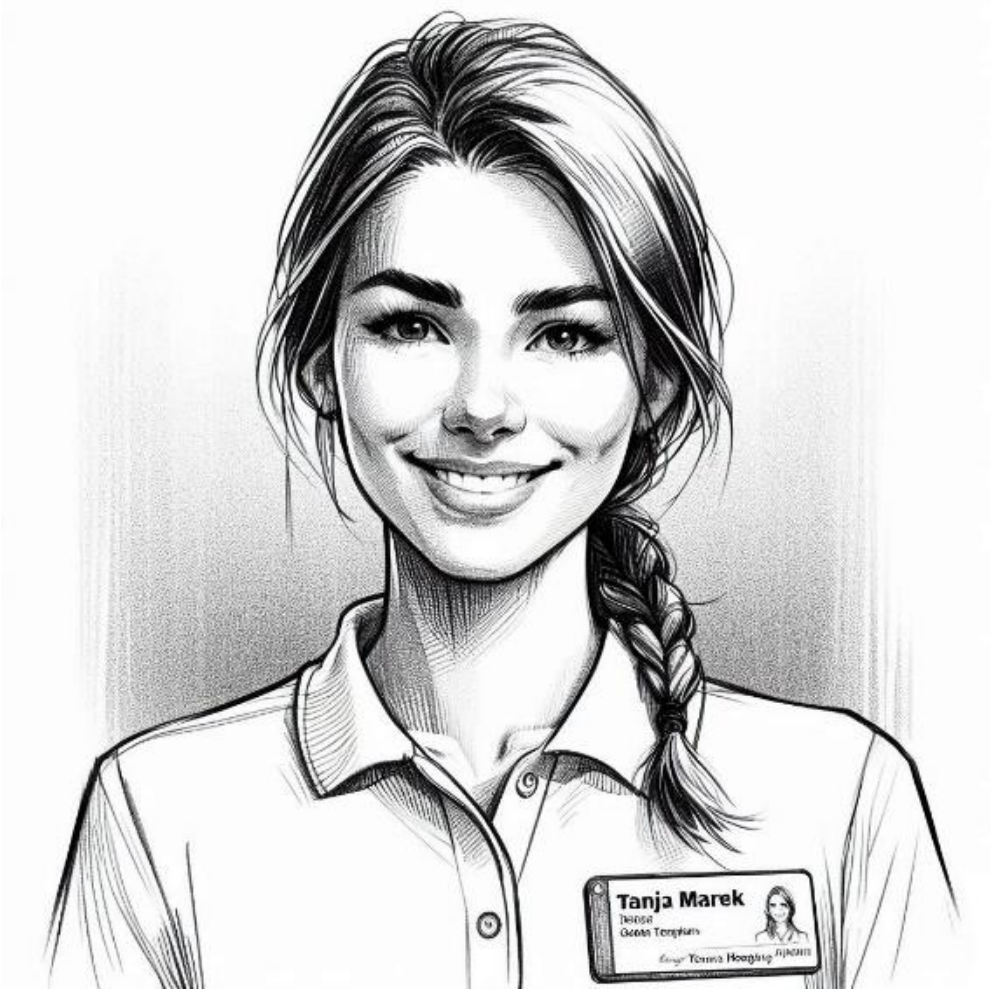

“Patients come first. Therefore, I want to provide them with a comprehensive overview of their current mobility status and motivate them to stay active.“

Age: 24 years
Position: Study assistant; qualified physiotherapist; plans to start a part-time degree program
Professional experience: 5 years
Institution: Multiple Sclerosis Center at a university hospital
Workload: Part-time (30 hours per week)

Goals

- Better overall overview of patient mobility
- Improved communication with patients

Area of Responsibility & Relevant Tasks

- Conduct tests (EDSS, jump and gait tests)
- Interpret test results
- Discuss results with the patient
- Document relevant abnormalities

Wishes & Expectations

- Relevant information at a glance
- Quick interpretation of data
- Track the patient’s progress over recent years
- Compare the patient with healthy individuals and other patients (by age, gender, and EDSS category)
- Provide the patient with relevant information to take away

Fears & Concerns

- Too much unnecessary information
- Poorly designed user interface
- Difficulty communicating the displayed information due to lack of patient-friendly presentation
- Unnecessarily complicated data export for the patient
- Insufficient integration into the workflow

Technical Affinity & Motivation

Technical Affinity

Dashboard Usage Motivation

Prior Experience with Data Visualization

Experience in Interpreting Gait Analysis Data

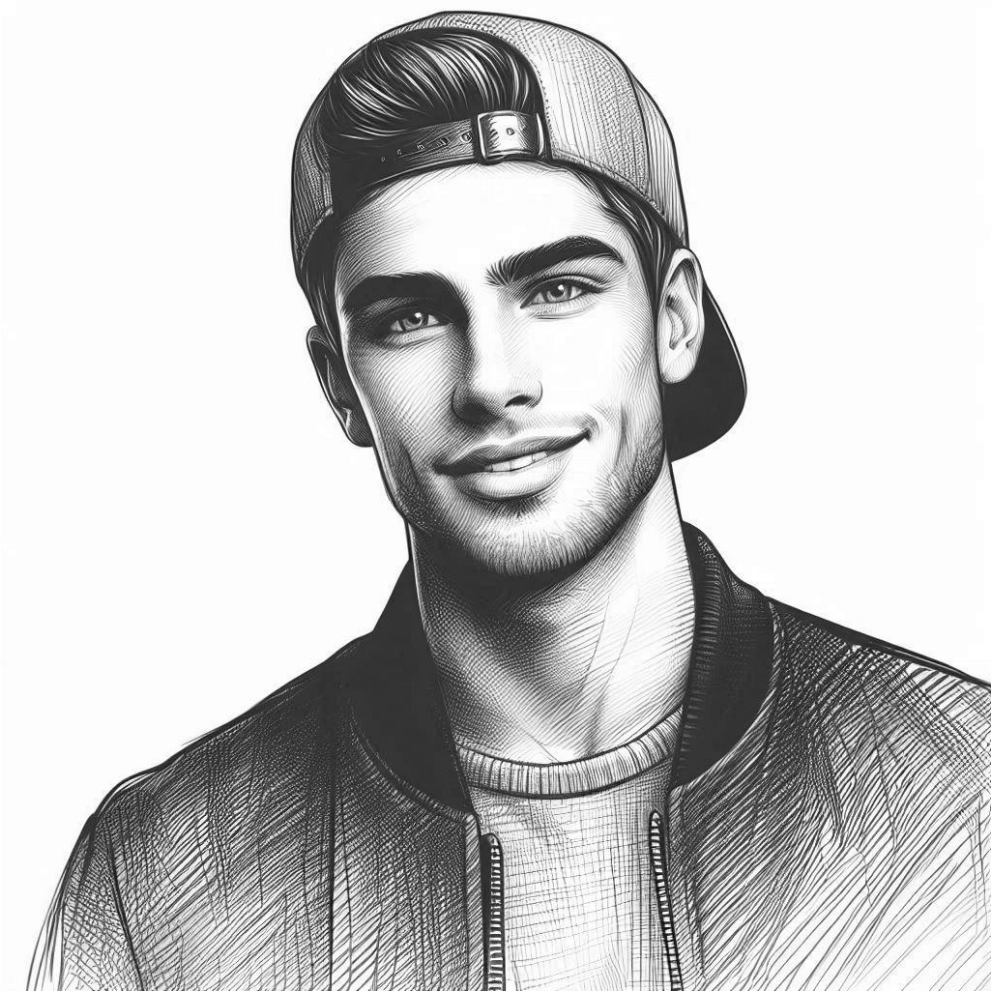

Tony Meinhardt

Patient with early-stage MS

"I want to stay as active and independent as possible. To do so, I want to use all available technologies and stay physically active in order to manage my condition in the best possible way."

Age: 21 years
Place of residence: Berlin
Occupation: Student, 4th semester in Business Administration
Marital status: Single

Goals

- Precise and detailed tracking of one's own mobility status

EDSS: 2.0 — Slight impairment

Jump ability

Walking ability

Medical History

MS Type: Relapsing-remitting MS (RRMS), i.e. symptoms occur in relapses followed by phases of remission
Diagnosis: 2 months ago; first-time patient at the MS Center UKDD
Symptoms: Fatigue, visual and sensory disturbances
Treatment: *Immunoprophylactic therapy*; immunomodulators

Wishes & Expectations

- Easily comprehensible information
- Accurate tracking of trends and initial prognoses
- Personalized physiotherapy recommendations
- Comparison with other patients to increase motivation
- Information preferably accessible via smartphone and smartwatch
- Direct forwarding of information to co-treating medical staff

Fears & Concerns

- Insufficient information to provide meaningful insights
- No option to view progress over time

Technical Affinity & Motivation

Technology Affinity

Dashboard Usage Motivation

Prior Experience with Data Visualization

Experience in Interpreting Gait Analysis Data

Devices used: Laptop, tablet, smartphone, and smartwatch

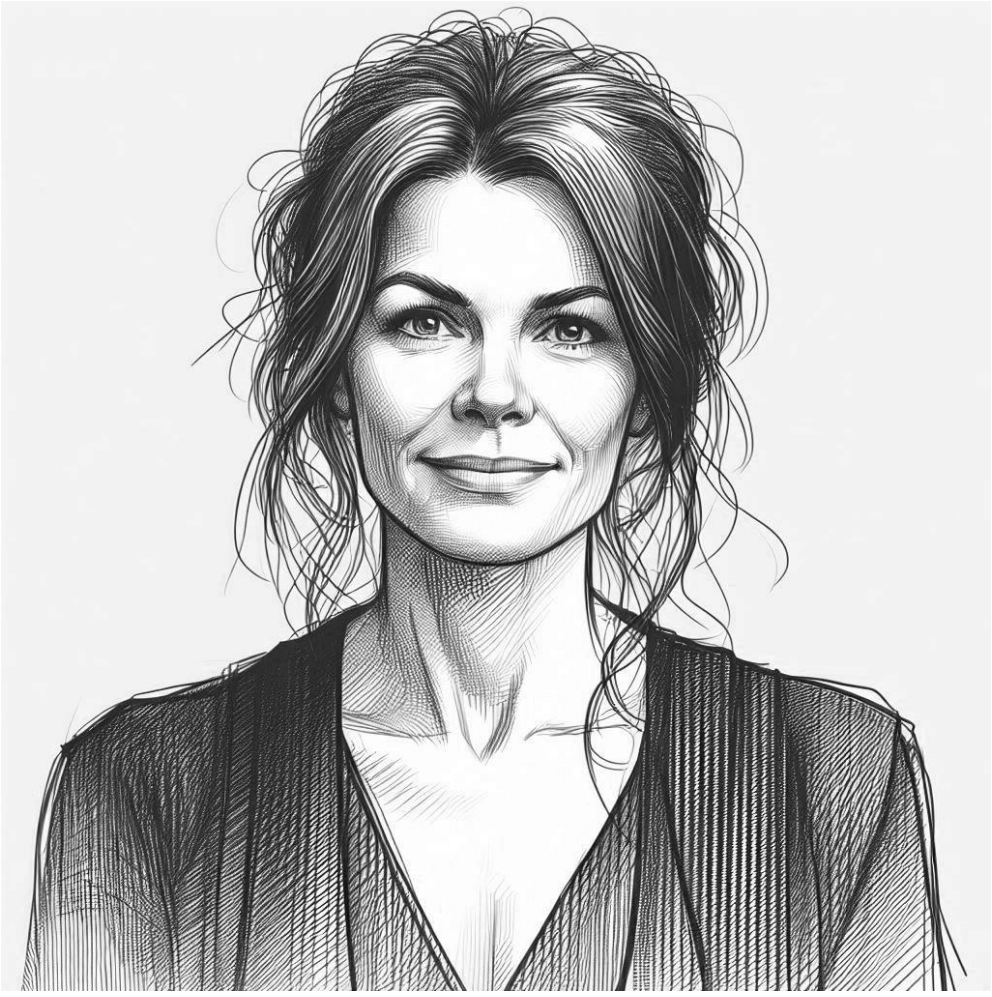

Age: 33 years
Place of residence: Görlitz (Saxony)
Occupation: Primary school teacher (full-time)
Marital status: Married (wants children)

Patrizia Schubert

Patient with relapsing-progressive MS

"I want to maintain as much independence as possible, treat my symptoms effectively, definitely become a mother, and then be fit and active for my child."

Medical History

**MS Type**:	Relapsing-remitting MS (RRMS), i.e. symptoms occur in relapses followed by phases of remission
**Diagnosis**:	5 years ago; patient at the MS Center UKDD for 5 years
**Symptoms**:	Difficulty walking long distances (from about 2 km), balance problems, occasional cognitive impairment
**Treatment**:	Immunomodulators, physiotherapy, occasional one-sided walking aid

Goals

- Clear and motivating tracking of one's own mobility status

EDSS: 3.5 — Moderate impairment

Jump ability

Walking ability

Wishes & Expectations

- Clear and simple graphics and diagrams of gait and movement patterns
- Dynamic display (graphics build up interactively when clicked)
- Deterioration of mobility status immediately visible
- Personalized physiotherapy recommendations
- Results accessible from home

Fears & Concerns

- Discouraging and difficult-to-understand information
- No progress display, making it impossible to see improvements or deterioration
- Security and privacy of personal health data

Technical Affinity & Motivation

Technology Affinity

Dashboard Usage Motivation

Prior Experience with Data Visualization

Experience in Interpreting Gait Analysis Data

Devices used: Laptop, tablet, smartphone, and fitness tracker

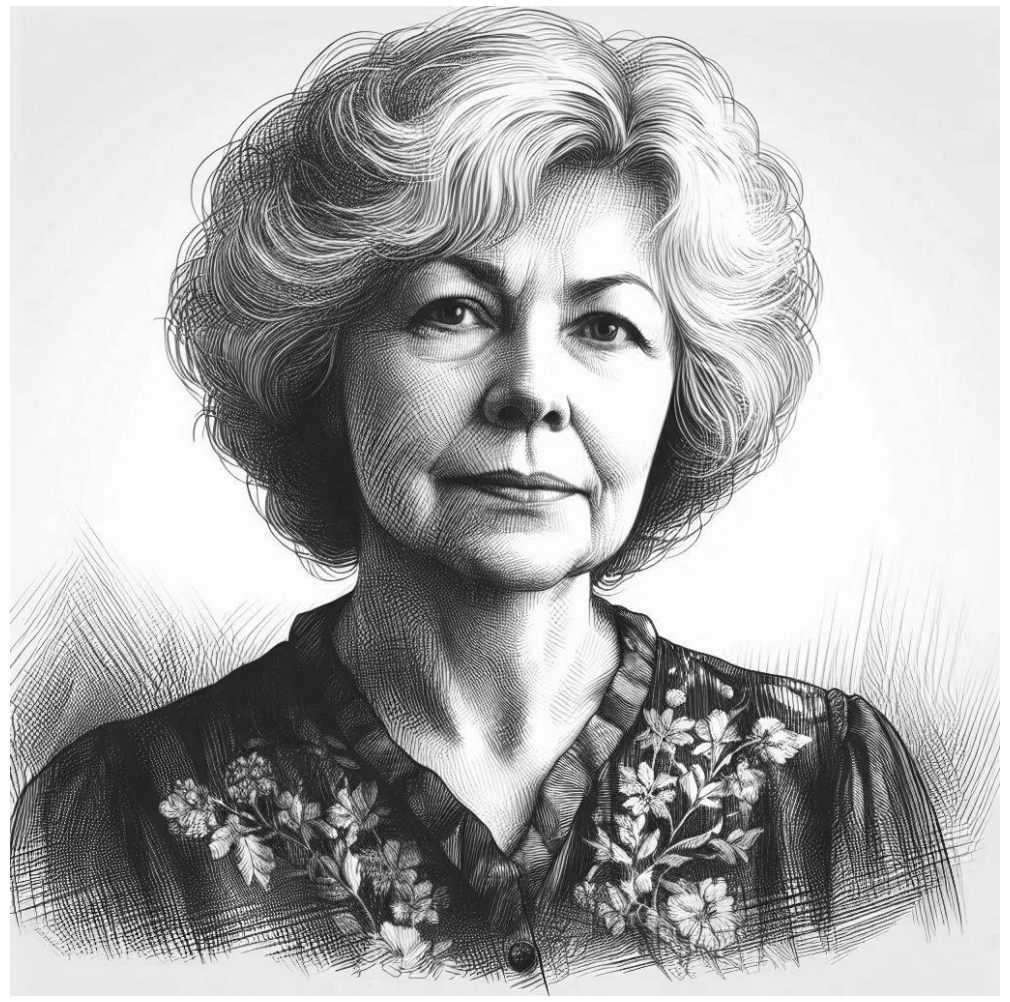

Brigitte Meyer

Patient with continuously progressive MS

“I want to continue taking part in family activities and maintain a close relationship with my children and grandchildren.”

Age: 58 years
Place of residence: Dresden (Saxony)
Occupation: Accountant, early retirement
Marital status: Widowed, two adult children

Goals

- Simple and easy-to-understand summary of one’s own mobility status

EDSS: 6.0 — Severe impairment

Jump ability

Walking ability

Medical History

MS Type: Secondary progressive multiple sclerosis (SPMS), which developed after about 12 years from an initially relapsing-remitting MS
Diagnosis: 15 years ago; patient at the MS Center UKDD for 8 years
Symptoms: Spasticity, fatigue, impaired bladder function, cognitive disturbances, pain, depression
Treatment: *Disease-modifying immunotherapy;* immunosuppressants, antidepressants, analgesics, physiotherapy, use of assistive devices (walker, bath aid)

Wishes & Expectations

- Simple, intuitive, and easy-to-understand presentation of information
- As concise a result evaluation as possible
- Personalized physiotherapy recommendations
- Option to share the information with co-treating medical professionals

Fears & Concerns

- The abundance of information is overwhelming and makes it difficult to filter out the relevant details
- Discouraging information
- Reluctance or hesitation to use modern technology
- Security and privacy of personal health data

Technical Affinity & Motivation

Technology Affinity

Dashboard Usage Motivation

Prior Experience with Data Visualization

Experience in Interpreting Gait Analysis Data

Devices used: Laptop, tablet, smartphone
