# Supplementary material for: User-centered design and formative evaluation of a gait dashboard for multidimensional walking assessment in multiple sclerosis
Source: Front Digit Health. 2026 Sep 8;8:1865736. doi: 10.3389/fdgth.2026.1865736 (PMC13593768; doi:10.3389/fdgth.2026.1865736)
Supplement: Supplementary file 5 [file Datasheet5.pdf]

List of User Requirements

#	User Requirement	Explanation	Priority
1	**Overview of total treatment progress:** “The user should be able to view and compare the values/data/results from a patient’s appointments at a glance.”	Comparing retrospective measurement values with the current state allows the analysis of parameters collected over time for the same patient. The patient receives an evaluation of current results in comparison to earlier measurements, starting from the initial visit. Patients also wish to have all results visible in one window so that individual files do not need to be opened separately.	1
2	**Intuitiveness of evaluations:** “The user should be presented with evaluations that are intuitively understandable.”	The displayed information must be designed so that patients can understand it with minimal explanation. Key findings should be visible at a glance. Terminology should be adapted to patient language, including: (1) German translations or fully German labels; (2) simple expressions with few technical terms; (3) explanations of values using everyday words (e.g., step time); and (4) written-out abbreviations (e.g., RMS, GC). Units should be shown on all axes and tables to enhance comprehension. The presentation should be so clear that patients, doctors, and medical staff without a motion science background can easily understand it.	1
3	**Aesthetic user interface:** “The dashboard interface should be visually appealing from the user’s perspective.”	The dashboard should be clearly structured and visually appealing, with a thoughtful color scheme, well-organized layout, and minimalist presentation of results. Colors and design clarity should help both patients and physicians easily understand and use the displayed information.	1
4	**Evaluation of the current state:** “The user should be able to view all relevant gait parameters on the dashboard.”	Parameters recorded during the test day (e.g., via gait mat, or motion sensors) should be displayed. This allows a compact summary of the patient’s current condition based on key measurement variables.	1
5	**Interpretation of values (qualitative):** “The user should be able to qualitatively interpret a patient’s values/data/results.”	Both current (actual) and longitudinal values should be presented graphically (e.g., in diagrams or traffic light visualizations). Parameters should be illustrated clearly, allowing users to see when significant thresholds are exceeded.	1
6	**Display of different domains:** “The results of a patient’s gait analysis should be displayed across different domains.”	The analysis results should be shown not only as individual parameters but also within defined domains. Deficits are represented in each domain. For gait analysis, potential domains include “Balance,” “Coordination,” and “Asymmetry.”	1

#	User Requirement	Explanation	Prio-rity
7	**Expandability / scalability of the dashboard:** “The user should be able to adjust the dashboard according to the test procedures (number/type).”	The dashboard should be extendable to integrate new tests or assessments. When a new test is added, its values and results should also appear in the dashboard. The system should be flexible enough to add or remove tests and parameters as needed.	1
8	**Intuitive usability:** “The user should be able to navigate and interact with the dashboard effortlessly.”	The dashboard should be barrier-free, allowing users to easily see where they can adjust or view data. An intuitive interface ensures that all functions are simple, clear, and accessible, supporting inclusive use.	1
9	**Comparison of different conditions:** “The user should be able to compare a patient’s results with those of a healthy cohort (of the same age group) and with patients who have similar limitations.”	The user should be able to compare patient results with those of healthy individuals and patients with similar impairments. The dashboard should provide functions to highlight such differences or deviations clearly. Patients also expressed interest in comparing changes and abnormalities relative to both healthy and similar groups.	1
10	**Recognition of threshold values:** “The user should be able to quickly recognize a patient’s threshold values.”	The user should easily recognize a patient’s threshold values, especially when specific cut-off values are exceeded. The dashboard should display clear markers, notifications, or warnings to highlight critical results.	1
11	**Display of specific action recommendations:** “The user should be shown specific action recommendations related to the gait analysis results.”	The dashboard should display specific recommendations related to the patient’s results. These instructions should align directly with identified deficits to support targeted interventions. Patients also requested actionable advice (e.g., physiotherapy suggestions).	1
12	**Result evaluation for the physician:** “The user should be able to communicate the results of a patient’s gait analysis to the physician.”	The user should be able to communicate a patient’s gait analysis results to the physician, either digitally or through a printed report. This feature should replace the current manual paper-based solution and enable efficient and clearly structured result transmission.	1
13	**Interpretation of values (quantitative):** “The user should be able to quantitatively interpret a patient’s values/data/results.”	The dashboard should display reference or normal ranges differentiated by age and/or sex. These reference values allow the patient’s current state to be compared and categorized.	2

#	User Requirement	Explanation	Prio-rity
14	**Sharing results with the patient:** "The user should be able to share the results of the gait analysis with the patient (in some form)."	After evaluation, the patient should receive a summary of results, either as a printout, handout, or electronic document (e.g., PDF). The patient report should focus on key findings, not the full dashboard content. Note: patients are divided on whether they want direct access; those who do prefer email or PDF delivery.	2
15	**Adaptability of reference values:** "The user should be able to adjust the reference values of each test."	Reference values help contextualize measurements but should be customizable and expandable (e.g., for research). These values may vary depending on disease stage, sex, or other factors. Ideally, the system should automatically select the most appropriate reference values for each comparison.	2
16	**Availability of values:** "The user should be able to view gait analysis results quickly and promptly."	The dashboard should have short loading times so that results appear immediately after testing.	3
17	**Integrated data storage:** "The user should be able to automatically and directly access a patient's values/parameters/results through the dashboard (without manual merging)."	The dashboard should be accessible at any time and include all relevant data from every measurement session. New measurements should be added, not overwrite previous data, ensuring a complete record.	3
18	**Display of PROMs:** "The user should be able to view a patient's PROMs results on the dashboard."	The user should be able to view the patient's Patient-Reported Outcome Measures (PROMs) directly in the dashboard.	3
19	**Customization of evaluations:** "The user should be able to generate value/parameter/result displays tailored to each patient."	The user should be able to customize values, parameters, and results for each patient's specific needs. Note: patients generally prefer that results not be personalized during explanations.	3
20	**Responsive dashboard design:** "The user should be able to use the dashboard easily regardless of screen size and/or device."	The dashboard should automatically adapt to different screen sizes and devices such as smartphones or tablets, ensuring consistent usability.	3
21	**Retrievability of previous dashboard views:** "The user should be able to access all dashboard views from previous appointments."	The user should be able to retrieve all previous dashboard views of a patient at any time.	3
22	**Accessibility from ORBIS:** "The user should be able to access the dashboard directly from the clinical workstation system (ORBIS)."	The user should be able to open the dashboard directly from the ORBIS clinical system.	3
