# Supplementary material for: User-centered design and formative evaluation of a gait dashboard for multidimensional walking assessment in multiple sclerosis
Source: Front Digit Health. 2026 Sep 8;8:1865736. doi: 10.3389/fdgth.2026.1865736 (PMC13593768; doi:10.3389/fdgth.2026.1865736)
Supplement: Supplementary file 6 [file Datasheet6.pdf]

Supplement Prototype V2

Note:

German labels are shown in **gray square brackets** to facilitate comparison with the German screenshots

→ e.g. *English:* “Overview”; *German:* [Übersicht]

MS Sprung- und Ganganalyse

Meyer, Luise | Patienten ID XXX74398 | Geburtsdatum 29.04.1955 | Alter 69 | Geschlecht Weiblich | BMI 26,5 | MS-Verlaufsform SPMS | Erstdiagnose 1995 | EDSS 5,0 | Medikament Immunsuppressiva

Übersicht | Sprunganalyse | Ganganalyse | Empfehlungen

22.06.2024

a

Sprunganalyse

Sprungtest	10-Second Hop-Test	Countermovement Jump	Single Leg Countermovement Jump
Leistungsfähigkeit	↘ 45 Ø 49 ■	→ 78 Ø 78 ●	↗ 32 Ø 23 ▲
Bewegungsenergie	↘ 50 Ø 55 ■	↘ 80 Ø 92 ●	→ 50 Ø 50 ■
Zeitparameter	↘ 45 Ø 58 ■	↗ 62 Ø 58 ■	→ 42 Ø 42 ■

Legende

Veränderung im Vergleich zu den letzten Visiten

↘ 45 Aktueller Wert
Ø 48 Durchschnittswert der letzten Visiten

↗ verbessert ↘ verschlechtert → gleichbleibend

Vergleich mit Peers

● über dem oder im Durchschnitt
■ leicht unter dem Durchschnitt
▲ deutlich unter dem Durchschnitt

b

Ganganalyse

Gangtest	Normales Gehen	Doppelaufgabe	Schnelles Gehen	Rückwärtsgehen	2-Minuten Gehtest
Parameter: (Kategorie)	Gehgeschwindigkeit (Geschwindigkeit)	Gehgeschwindigkeit (Geschwindigkeit)	Schrittzeit (Rhythmus)	Schrittzeit (Rhythmus)	Zurückgelegte Wegstrecke (Geschwindigkeit)
Wert:	→ 110,2 cm/s Ø 109,5 cm/s ■	→ 109,1 cm/s Ø 108,8 cm/s ●	L: ↗ 0,60/5,5 s Ø 0,63/5,5 s ■ R: ↗ 0,58/4,5 s Ø 0,64/4,5 s ■	L: → 0,75/4,8 s Ø 0,73/4,7 s ▲ R: ↘ 0,78/5,7 s Ø 0,71/5,0 s ▲	↗ 136,02 m Ø 129,71 ■

Gleichgewicht	Balance-Test mit geöffneten Augen	Balance-Test mit geschlossenen Augen
Schwankungs-diagramm	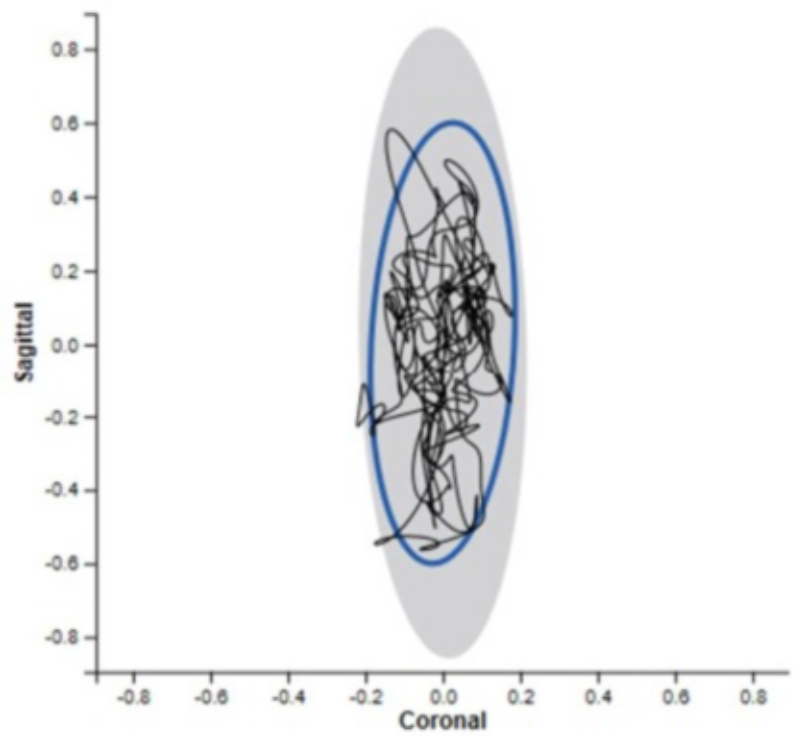	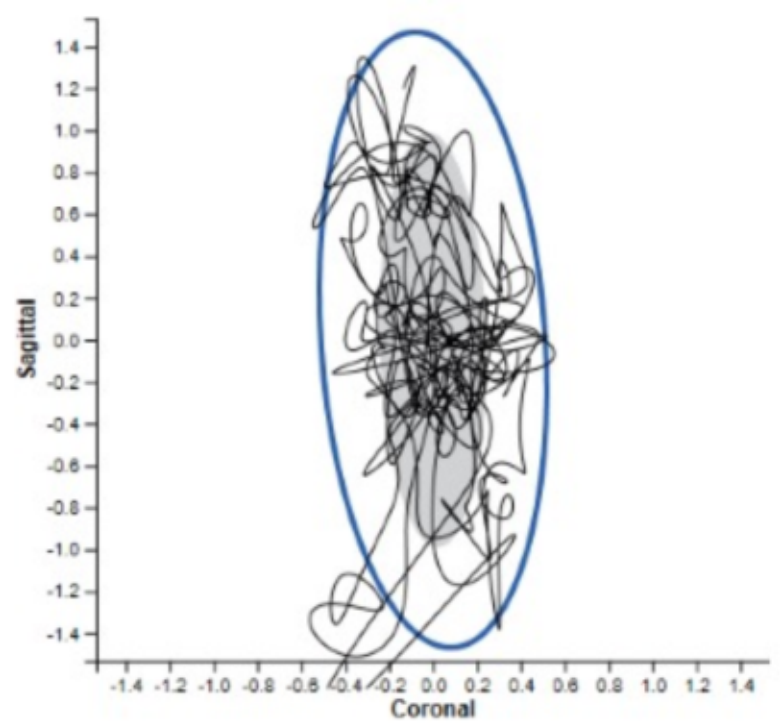
Schwankungsbereich	→ 0,34° Ø 0,34° ■	↗ 0,35° Ø 0,37° ■

Kritische Werte

Sprung
10-Second Hop-Test: Leistungsfähigkeit I Anzahl der Sprünge: 2

Gang
Normales Gehen: Rhythmus I Bipedale Standphase (nach Perry) links: 36%

Normales Gehen: Asymmetrie I Fußstellung rechts nach innen: 9°

Empfehlungen

Sprung
Sprungkraft-Training, Physiotherapie

Gang
Kräftigungsübungen, Balance-Training, Propriozeptives Training

Kräftigungsübungen, Dehnübungen, Propriozeptives Training

a (purple dashed line)

Non-gait parameters

b

Gait analysis parameter

MS Sprung- und Ganganalyse

Meyer, Luise	Patienten ID	Geburtsdatum	Alter	Geschlecht	BMI	MS-Verlaufsform	Erstdiagnose	EDSS	Medikament
	XXX74398	29.04.1955	69	Weiblich	26,5	SPMS	1995	5,0	Immunsuppressiva

Übersicht | Sprunganalyse | Ganganalyse | Empfehlungen

22.06.2024

Sprunganalyse

Sprungtest	10-Second Hop-Test	Countermovement Jump	Single Leg Countermovement Jump
Leistungsfähigkeit	↘ 45 Ø 49 ■	→ 78 Ø 78 ●	↗ 32 Ø 23 ▲
Bewegungsenergie	↘ 50 Ø 55 ■	↘ 80 Ø 92 ●	→ 50 Ø 50 ■
Zeitparameter	↘ 45 Ø 58 ■	↗ 62 Ø 58 ■	→ 42 Ø 42 ■

Legende ⓘ

Veränderung im Vergleich zu den letzten Visiten

↘ 45 Aktueller Wert
Ø 48 Durchschnittswert der letzten Visiten

↗ verbessert ↘ verschlechtert → gleichbleibend

Vergleich mit Peers

- ● über dem oder im Durchschnitt
- ■ leicht unter dem Durchschnitt
- ▲ deutlich unter dem Durchschnitt

Ganganalyse

Gangtest	Normales Gehen	Doppelaufgabe	Schnelles Gehen	Rückwärtsgehen	2-Minuten Gehtest
Parameter: (Kategorie)	Gehgeschwindigkeit (Geschwindigkeit)	Gehgeschwindigkeit (Geschwindigkeit)	Schrittzeit (Rhythmus)	Schrittzeit (Rhythmus)	Zurückgelegte Wegstrecke (Geschwindigkeit)
Wert:	→ 110,2 cm/s Ø 109,5 cm/s ■	→ 109,1 cm/s Ø 108,8 cm/s ●	L: ↗ 0,60/5,5 s Ø 0,63/5,5 s ■<br>R: ↗ 0,58/4,5 s Ø 0,64/4,5 s ■	L: → 0,75/4,8 s Ø 0,73/4,7 s ▲<br>R: ↘ 0,78/5,7 s Ø 0,71/5,0 s ▲	↗ 136,02 m Ø 129,71 ■

Gleichgewicht	Balance-Test mit geöffneten Augen	Balance-Test mit geschlossenen Augen
Schwankungs-diagramm	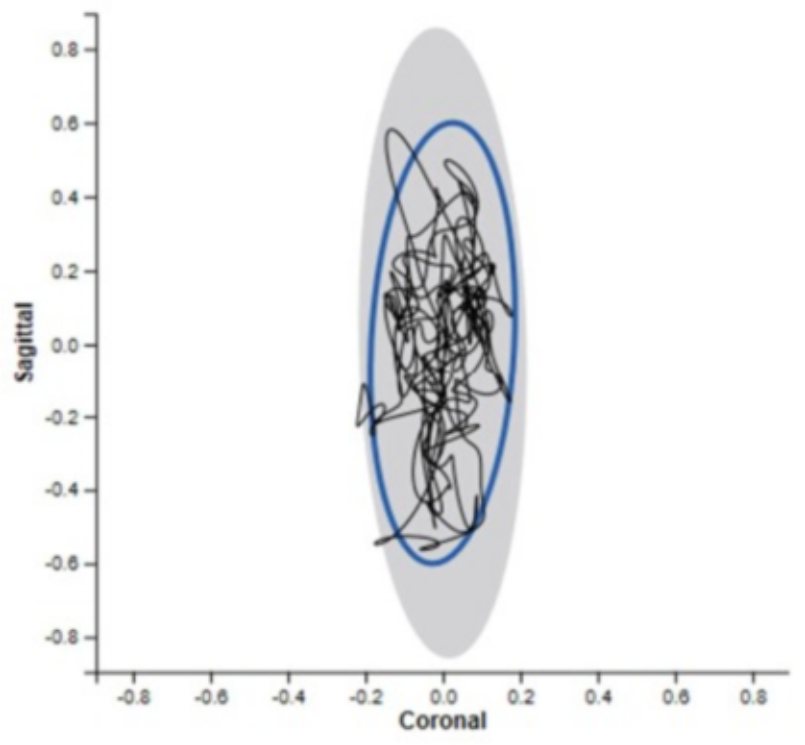	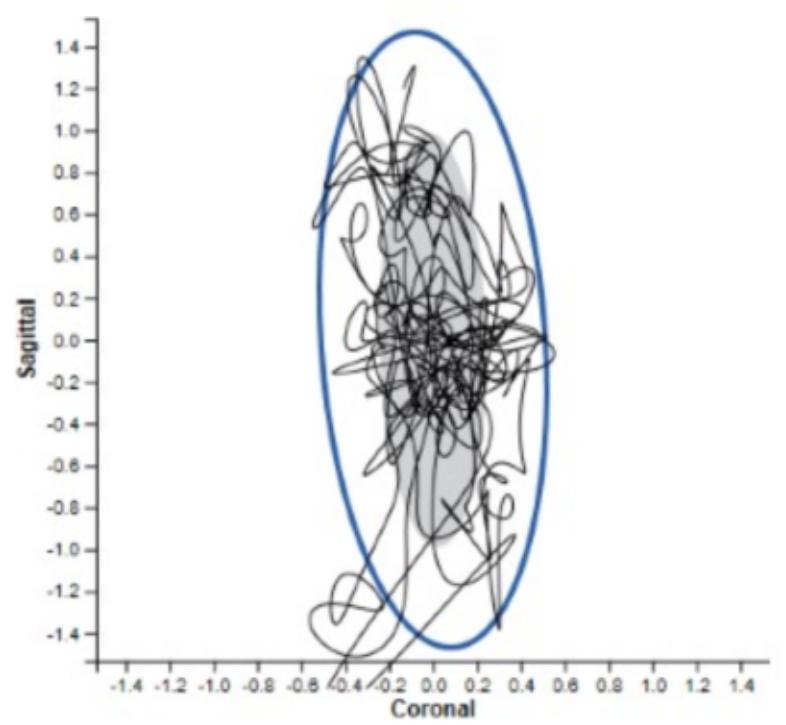
Schwankungsbereich	→ 0,34° Ø 0,34° ■	↗ 0,35° Ø 0,37° ■

Kritische Werte

Sprung

10-Second Hop-Test: Leistungsfähigkeit I
Anzahl der Sprünge: 2

Gang

Normales Gehen: Rhythmus I
Bipedale Standphase (nach Perry)
links: 36%

Normales Gehen: Asymmetrie I
Fußstellung rechts nach innen: 9°

Empfehlungen

Sprung

Sprungkraft-Training,
Physiotherapie

Gang

Kräftigungsübungen, Balance-Training,
Propriozeptives Training

Kräftigungsübungen, Dehnübungen,
Propriozeptives Training

- **Overview page** [Übersicht]: Displays all current results at a glance
- The header shows the name and some important information about the selected patient.

MS Sprung- und Ganganalyse

Meyer, Luise

Patienten ID	Geburtsdatum	Alter	Geschlecht	BMI	MS-Verlaufsform	Erstdiagnose	EDSS	Medikament
XXX74398	29.04.1955	69	Weiblich	26,5	SPMS	1995	5,0	Immunsuppressiva

Übersicht | Sprunganalyse | Ganganalyse | Empfehlungen

22.06.2024

Sprunganalyse

Sprungtest	10-Second Hop-Test	Countermovement Jump	Single Leg Countermovement Jump
Leistungsfähigkeit	↘ 45 Ø 49 ■	→ 78 Ø 78 ●	↗ 32 Ø 23 ▲
Bewegungsenergie	↘ 50 Ø 55 ■	↘ 80 Ø 92 ●	→ 50 Ø 50 ■
Zeitparameter	↘ 45 Ø 58 ■	↗ 62 Ø 58 ■	→ 42 Ø 42 ■

Legende

Veränderung im Vergleich zu den letzten Visiten

45 Aktueller Wert
Ø 48 Durchschnittswert der letzten Visiten

↗ verbessert ↘ verschlechtert → gleichbleibend

Vergleich mit Peers

- ● über dem oder im Durchschnitt
- ■ leicht unter dem Durchschnitt
- ▲ deutlich unter dem Durchschnitt

Ganganalyse

Gangtest	Normales Gehen	Doppelaufgabe	Schnelles Gehen	Rückwärtsgehen	2-Minuten Gehtest
Parameter: (Kategorie)	Gehgeschwindigkeit (Geschwindigkeit)	Gehgeschwindigkeit (Geschwindigkeit)	Schrittzeit (Rhythmus)	Schrittzeit (Rhythmus)	Zurückgelegte Wegstrecke (Geschwindigkeit)
Wert:	→ **110,2 cm/s** Ø 109,5 cm/s ■	→ **109,1 cm/s** Ø 108,8 cm/s ●	L: ↗ **0,60/5,5 s** Ø 0,63/5,5 s ■<br>R: ↗ **0,58/4,5 s** Ø 0,64/4,5 s ■	L: → **0,75/4,8 s** Ø 0,73/4,7 s ▲<br>R: ↘ **0,78/5,7 s** Ø 0,71/5,0 s ▲	↗ **136,02 m** Ø 129,71 ■

Gleichgewicht	Balance-Test mit geöffneten Augen	Balance-Test mit geschlossenen Augen
Schwankungsdiagramm	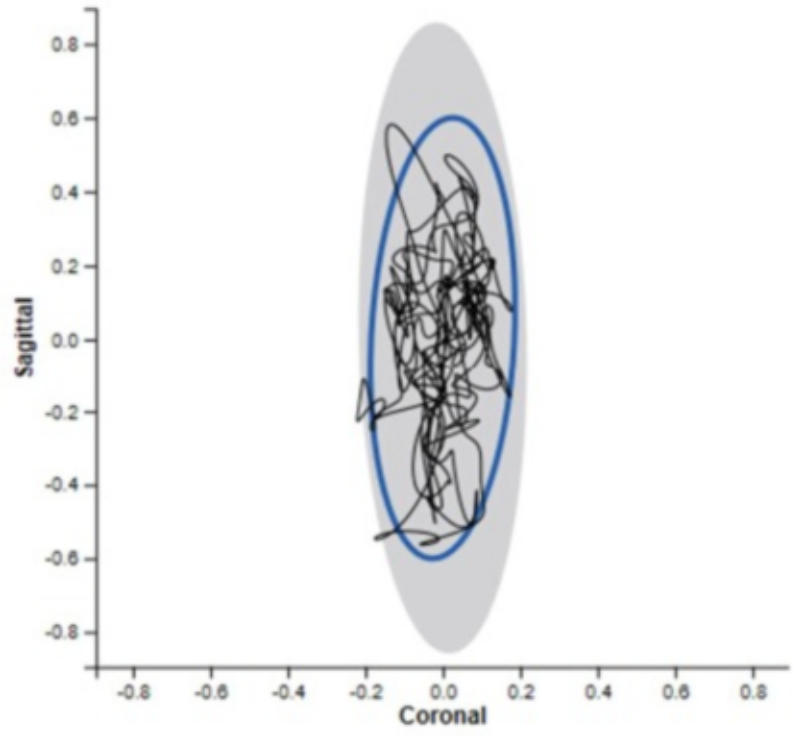	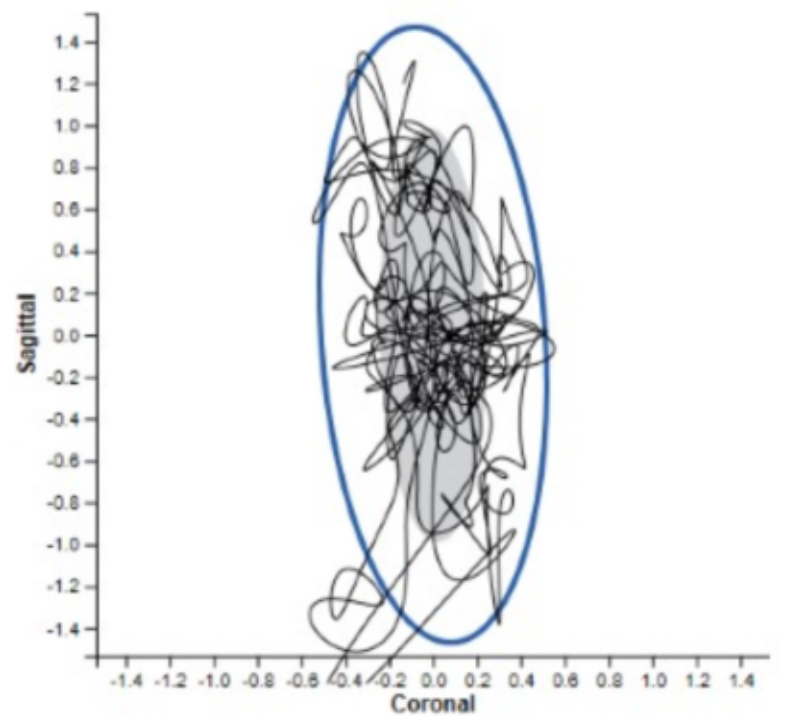
Schwankungsbereich	→ 0,34° Ø 0,34° ■	↗ 0,35° Ø 0,37° ■

Kritische Werte

Sprung
10-Second Hop-Test: Leistungsfähigkeit I Anzahl der Sprünge: 2

Gang
Normales Gehen: Rhythmus I Bipedale Standphase (nach Perry) links: 36%

Normales Gehen: Asymmetrie I Fußstellung rechts nach innen: 9°

Empfehlungen

Sprung
Sprungkraft-Training, Physiotherapie

Gang
Kräftigungsübungen, Balance-Training, Propriozeptives Training

Kräftigungsübungen, Dehnübungen, Propriozeptives Training

- Under the heading **"Gait Analysis"** [Ganganalyse], each gait test displays selected parameters that best reflect the patient's current condition.
- For each gait test, one **current key parameter** is shown in **bold.**
- For normal walking [Normales Gehen] and the dual-task test [Doppelaufgabe], this parameter is walking speed.
- Below, the **average value of all previous visits** Ø is displayed in smaller text.
- An **arrow** indicates how the current value compares to previous tests - showing whether the performance is better ↗, about the same →, or worse ↘.

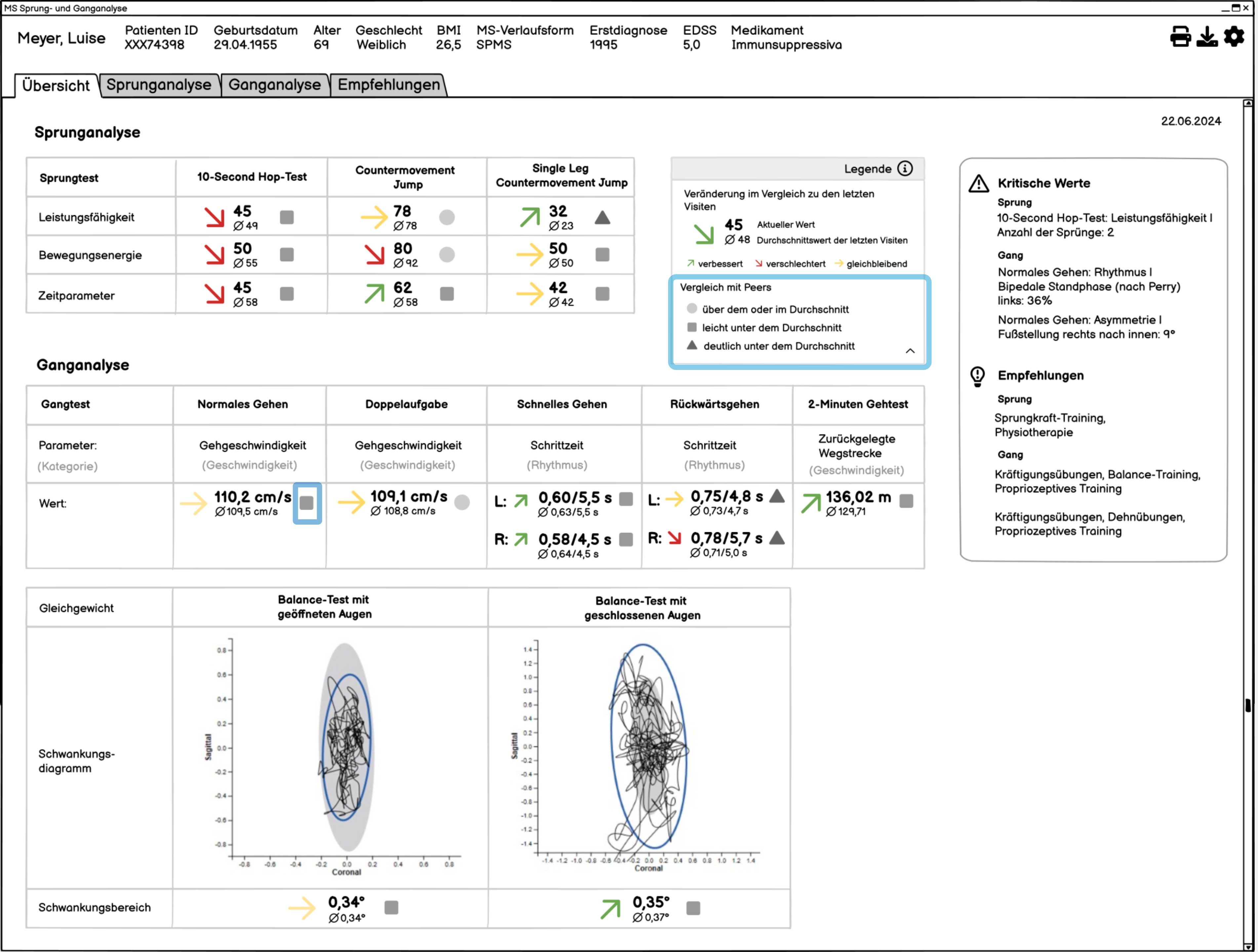

- Next to each value, **a geometric shape** indicates how the patient performs compared to **other patients of similar age and disease severity.**
- These comparison groups are referred to as **"peers"** (as shown in the **legend** [Legende], where all numbers and symbols are explained):

  Legend
  Comparison with peers

  ● above or at average

  ■ slightly below average

  ▲ clearly below average

- In the example shown, the current walking speed during normal walking [Normales Gehen] is slightly below the peer average, while in the dual-task test [Doppelaufgabe], the walking speed is at or above the peer average.

MS Sprung- und Ganganalyse

Meyer, Luise	Patienten ID	Geburtsdatum	Alter	Geschlecht	BMI	MS-Verlaufsform	Erstdiagnose	EDSS	Medikament
	XXX74398	29.04.1955	69	Weiblich	26,5	SPMS	1995	5,0	Immunsuppressiva

Übersicht | Sprunganalyse | Ganganalyse | Empfehlungen

22.06.2024

Sprunganalyse

Sprungtest	10-Second Hop-Test	Countermovement Jump	Single Leg Countermovement Jump
Leistungsfähigkeit	↘ 45 Ø 49 ■	→ 78 Ø 78 ●	↗ 32 Ø 23 ▲
Bewegungsenergie	↘ 50 Ø 55 ■	↘ 80 Ø 92 ●	→ 50 Ø 50 ■
Zeitparameter	↘ 45 Ø 58 ■	↗ 62 Ø 58 ■	→ 42 Ø 42 ■

Legende ⓘ

Veränderung im Vergleich zu den letzten Visiten

↘ 45 Aktueller Wert
Ø 48 Durchschnittswert der letzten Visiten

↗ verbessert ↘ verschlechtert → gleichbleibend

Vergleich mit Peers

● über dem oder im Durchschnitt
■ leicht unter dem Durchschnitt
▲ deutlich unter dem Durchschnitt

⚠ Kritische Werte

Sprung

10-Second Hop-Test: Leistungsfähigkeit I Anzahl der Sprünge: 2

Gang

Normales Gehen: Rhythmus I Bipedale Standphase (nach Perry) links: 36%

Normales Gehen: Asymmetrie I Fußstellung rechts nach innen: 9°

Empfehlungen

Sprung

Sprungkraft-Training, Physiotherapie

Gang

Kräftigungsübungen, Balance-Training, Propriozeptives Training

Kräftigungsübungen, Dehnübungen, Propriozeptives Training

Ganganalyse

Gangtest	Normales Gehen	Doppelaufgabe	Schnelles Gehen	Rückwärtsgehen	2-Minuten Gehtest
Parameter: (Kategorie)	Gehgeschwindigkeit (Geschwindigkeit)	Gehgeschwindigkeit (Geschwindigkeit)	Schrittzeit (Rhythmus)	Schrittzeit (Rhythmus)	Zurückgelegte Wegstrecke (Geschwindigkeit)
Wert:	→ 110,2 cm/s Ø 109,5 cm/s ■	→ 109,1 cm/s Ø 108,8 cm/s ●	L: ↗ 0,60/5,5 s Ø 0,63/5,5 s ■<br>R: ↗ 0,58/4,5 s Ø 0,64/4,5 s ■	L: → 0,75/4,8 s Ø 0,73/4,7 s ▲<br>R: ↘ 0,78/5,7 s Ø 0,71/5,0 s ▲	↗ 136,02 m Ø 129,71 ■

Gleichgewicht	Balance-Test mit geöffneten Augen	Balance-Test mit geschlossenen Augen
Schwankungs-diagramm	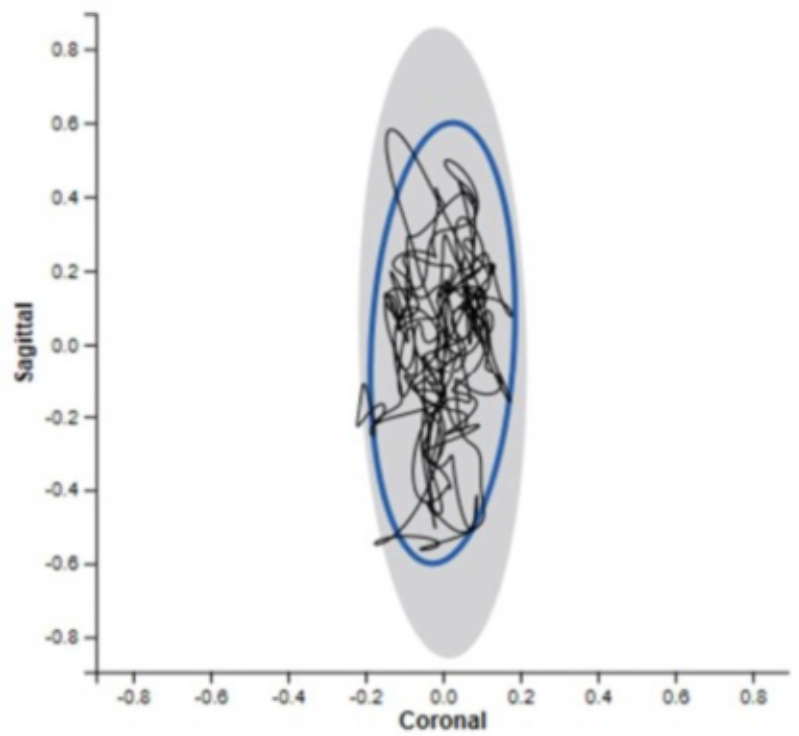	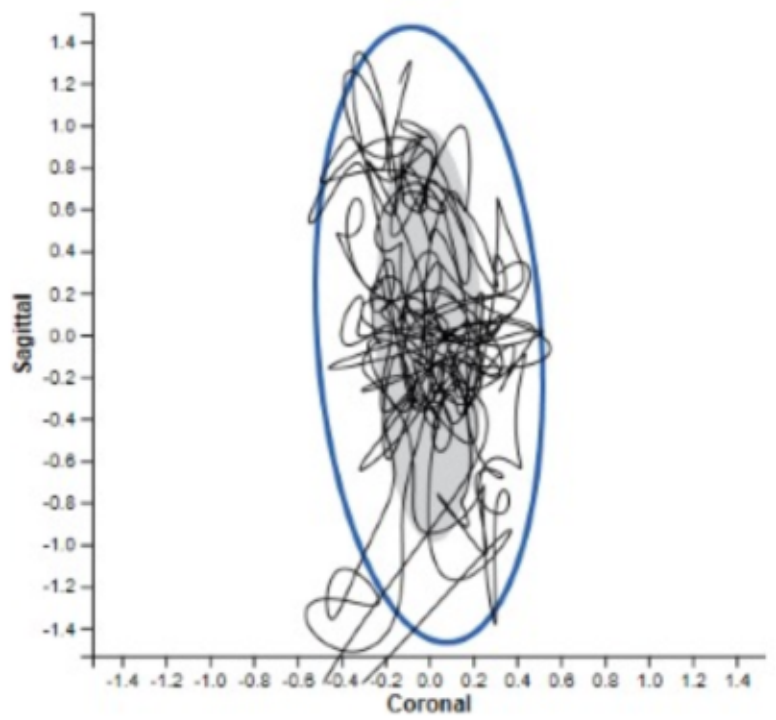
Schwankungsbereich	→ 0,34° Ø 0,34° ■	↗ 0,35° Ø 0,37° ■

- The **balance section** [Gleichgewicht] displays the results of the balance tests performed with **eyes open** [geöffnete Augen] and **eyes closed** [geschlossene Augen].
- For each condition, the current **sway diagram** [Schwankungs-diagramm] is shown.
- The **gray area** represents the **normal sway range** of healthy individuals.
- The **blue circle** indicates the **patient's individual sway range**.
- Below the diagram, **the sway angle (in degrees)** is displayed, along with an individual assessment and a comparison to peers.

MS Sprung- und Ganganalyse

Meyer, Luise	Patienten ID	Geburtsdatum	Alter	Geschlecht	BMI	MS-Verlaufsform	Erstdiagnose	EDSS	Medikament
	XXX74398	29.04.1955	69	Weiblich	26,5	SPMS	1995	5,0	Immunsuppressiva

Übersicht | Sprunganalyse | Ganganalyse | Empfehlungen

22.06.2024

Sprunganalyse

Sprungtest	10-Second Hop-Test	Countermovement Jump	Single Leg Countermovement Jump
Leistungsfähigkeit	↘ 45 Ø 49 ■	→ 78 Ø 78 ●	↗ 32 Ø 23 ▲
Bewegungsenergie	↘ 50 Ø 55 ■	↘ 80 Ø 92 ●	→ 50 Ø 50 ■
Zeitparameter	↘ 45 Ø 58 ■	↗ 62 Ø 58 ■	→ 42 Ø 42 ■

Legende

Veränderung im Vergleich zu den letzten Visiten

↘ 45 Aktueller Wert
Ø 48 Durchschnittswert der letzten Visiten

↗ verbessert ↘ verschlechtert → gleichbleibend

Vergleich mit Peers

● über dem oder im Durchschnitt
■ leicht unter dem Durchschnitt
▲ deutlich unter dem Durchschnitt

Kritische Werte

Sprung

10-Second Hop-Test: Leistungsfähigkeit I Anzahl der Sprünge: 2

Gang

Normales Gehen: Rhythmus I Bipedale Standphase (nach Perry) links: 36%

Normales Gehen: Asymmetrie I Fußstellung rechts nach innen: 9°

Empfehlungen

Sprung

Sprungkraft-Training, Physiotherapie

Gang

Kräftigungsübungen, Balance-Training, Propriozeptives Training

Kräftigungsübungen, Dehnübungen, Propriozeptives Training

Ganganalyse

Gangtest	Normales Gehen	Doppelaufgabe	Schnelles Gehen	Rückwärtsgehen	2-Minuten Gehtest
Parameter: (Kategorie)	Gehgeschwindigkeit (Geschwindigkeit)	Gehgeschwindigkeit (Geschwindigkeit)	Schrittzeit (Rhythmus)	Schrittzeit (Rhythmus)	Zurückgelegte Wegstrecke (Geschwindigkeit)
Wert:	→ 110,2 cm/s Ø 109,5 cm/s ■	→ 109,1 cm/s Ø 108,8 cm/s ●	L: ↗ 0,60/5,5 s Ø 0,63/5,5 s ■<br>R: ↗ 0,58/4,5 s Ø 0,64/4,5 s ■	L: → 0,75/4,8 s Ø 0,73/4,7 s ▲<br>R: ↘ 0,78/5,7 s Ø 0,71/5,0 s ▲	↗ 136,02 m Ø 129,71 ■

Gleichgewicht	Balance-Test mit geöffneten Augen	Balance-Test mit geschlossenen Augen
Schwankungs-diagramm	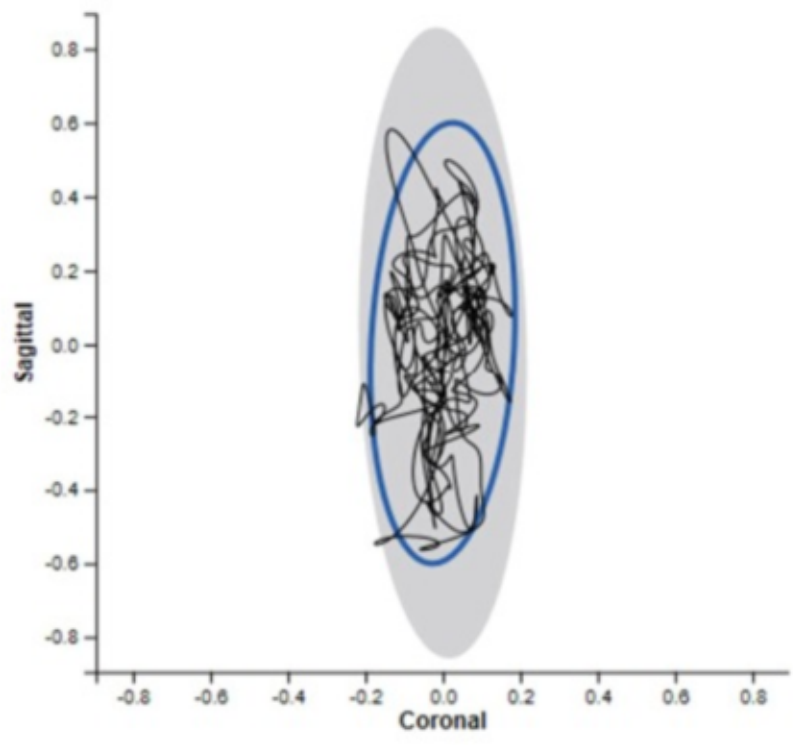	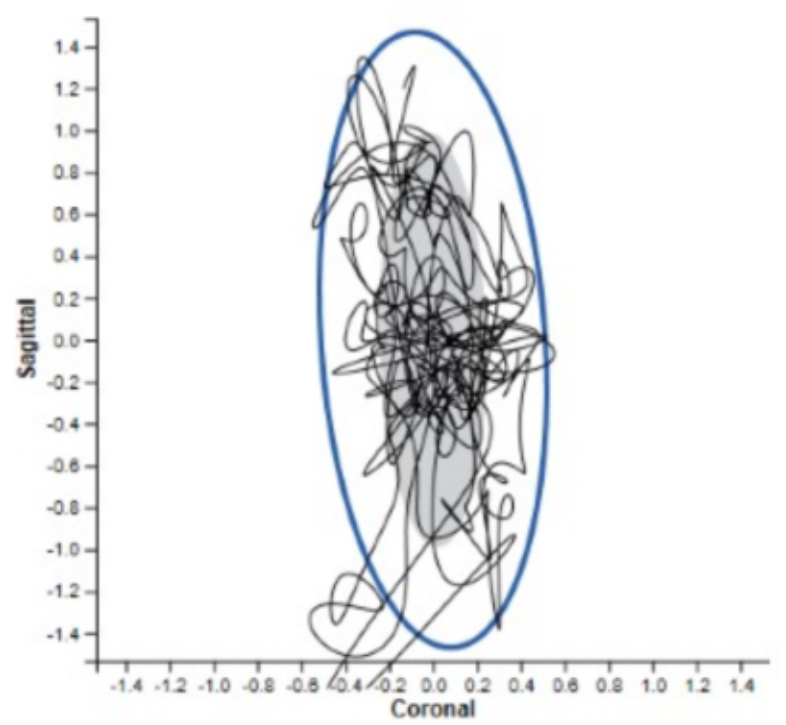
Schwankungsbereich	→ 0,34° Ø 0,34° ■	↗ 0,35° Ø 0,37° ■

- The overview also **highlights values** that were classified as **critical** ⚠ [Kritische Werte].
- Based on these findings, initial **recommendations** 💡 [Empfehlungen] for improvement are provided.

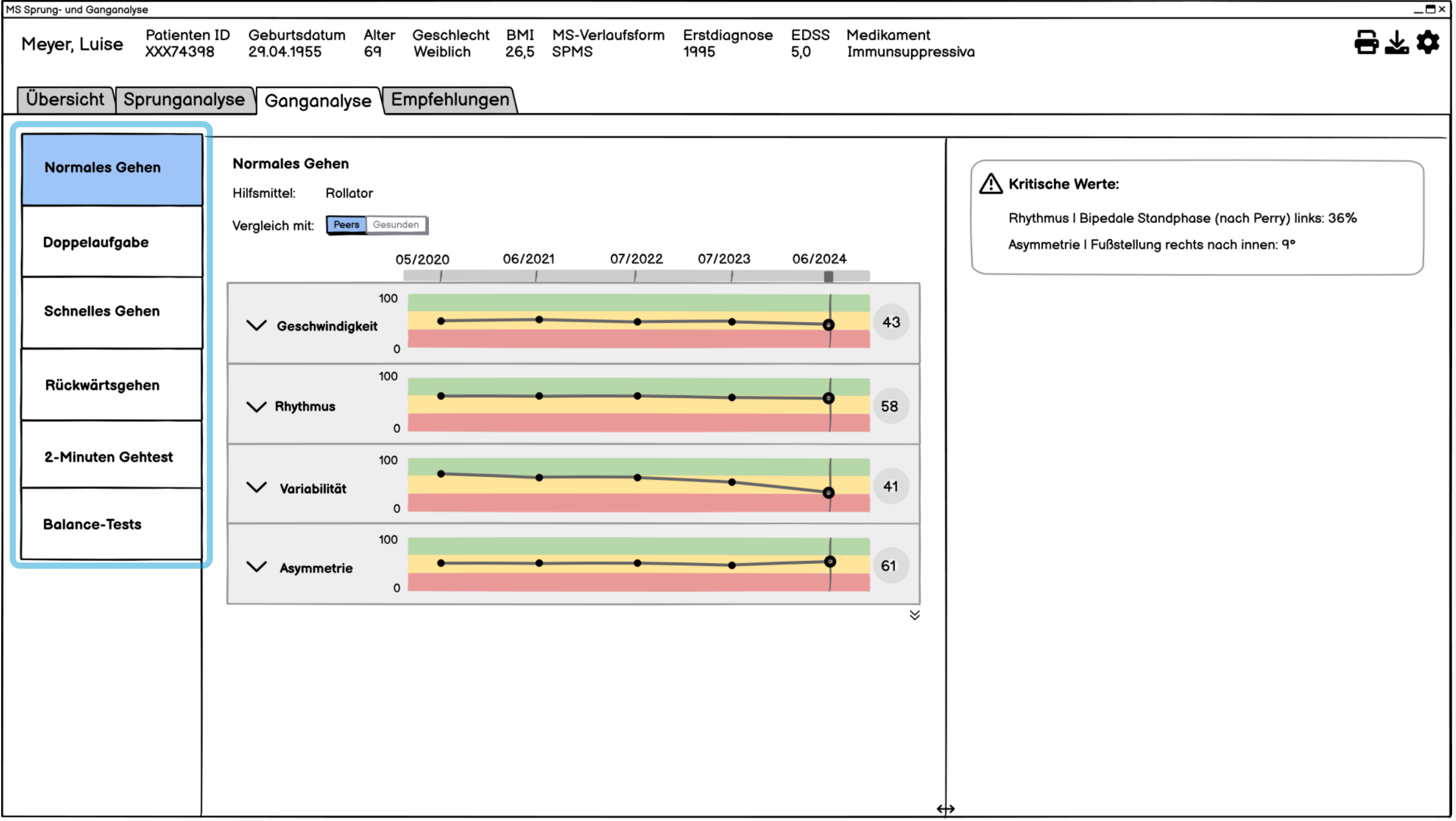

- Under the **„Gait Analysis“** [Ganganalyse] **tab**, detailed results of the gait tests can be viewed.
- Users can see the individual values for the selected test - in this example, **“Normal Walking.”** [Normales Gehen]
- On the left, each gait test can be selected via a button to display its corresponding values.
- The currently selected test, Normal Walking, is highlighted in blue.

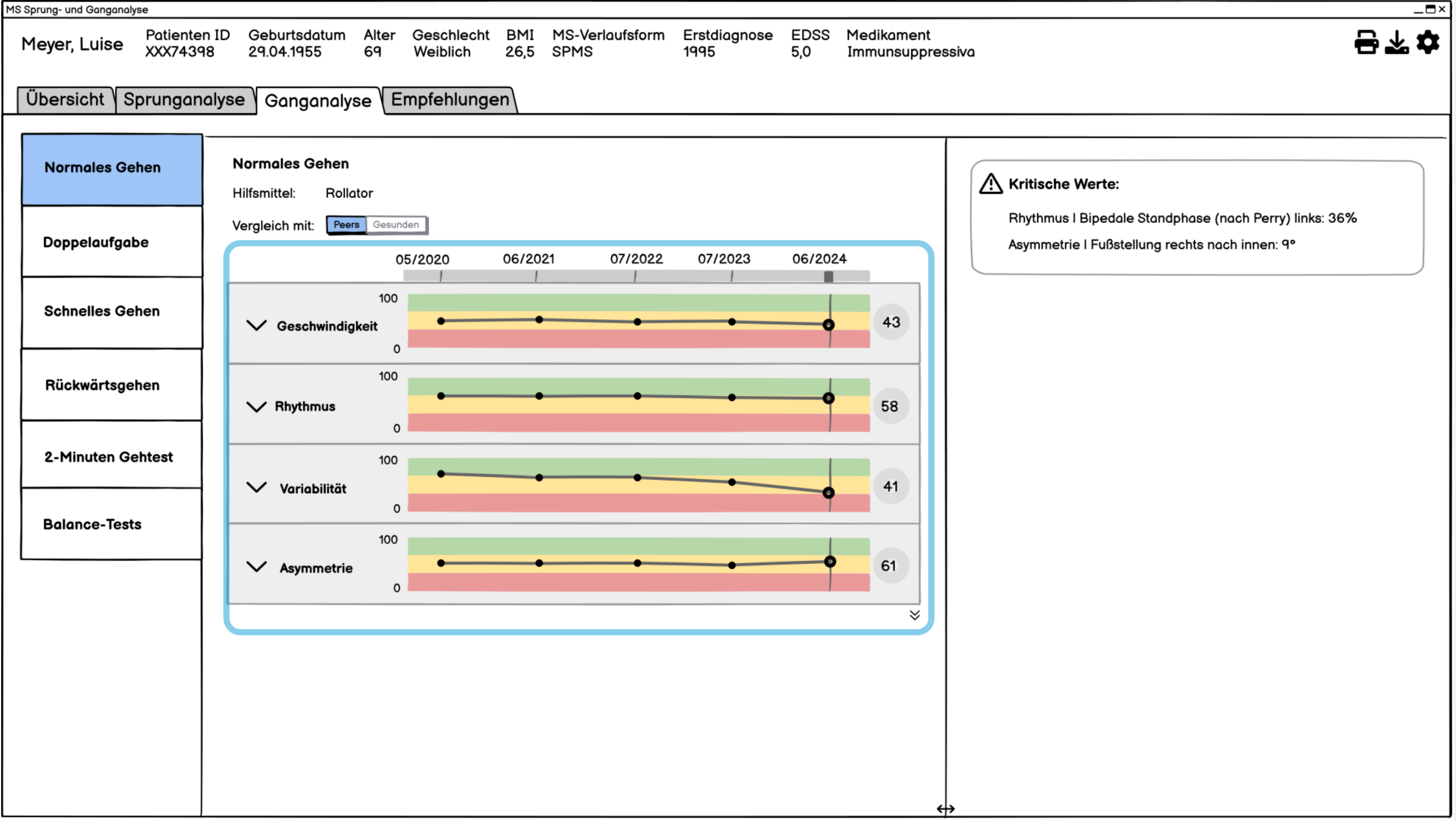

- In the center, the measured values for the selected test are displayed, **grouped by categories** and summarized in an **overall score.**
- In the first gray-shaded chart, the patient's performance across all speed-related parameters in the Normal Walking test over multiple visits is shown.
- The different measurement time points are indicated in a timeline at the top.

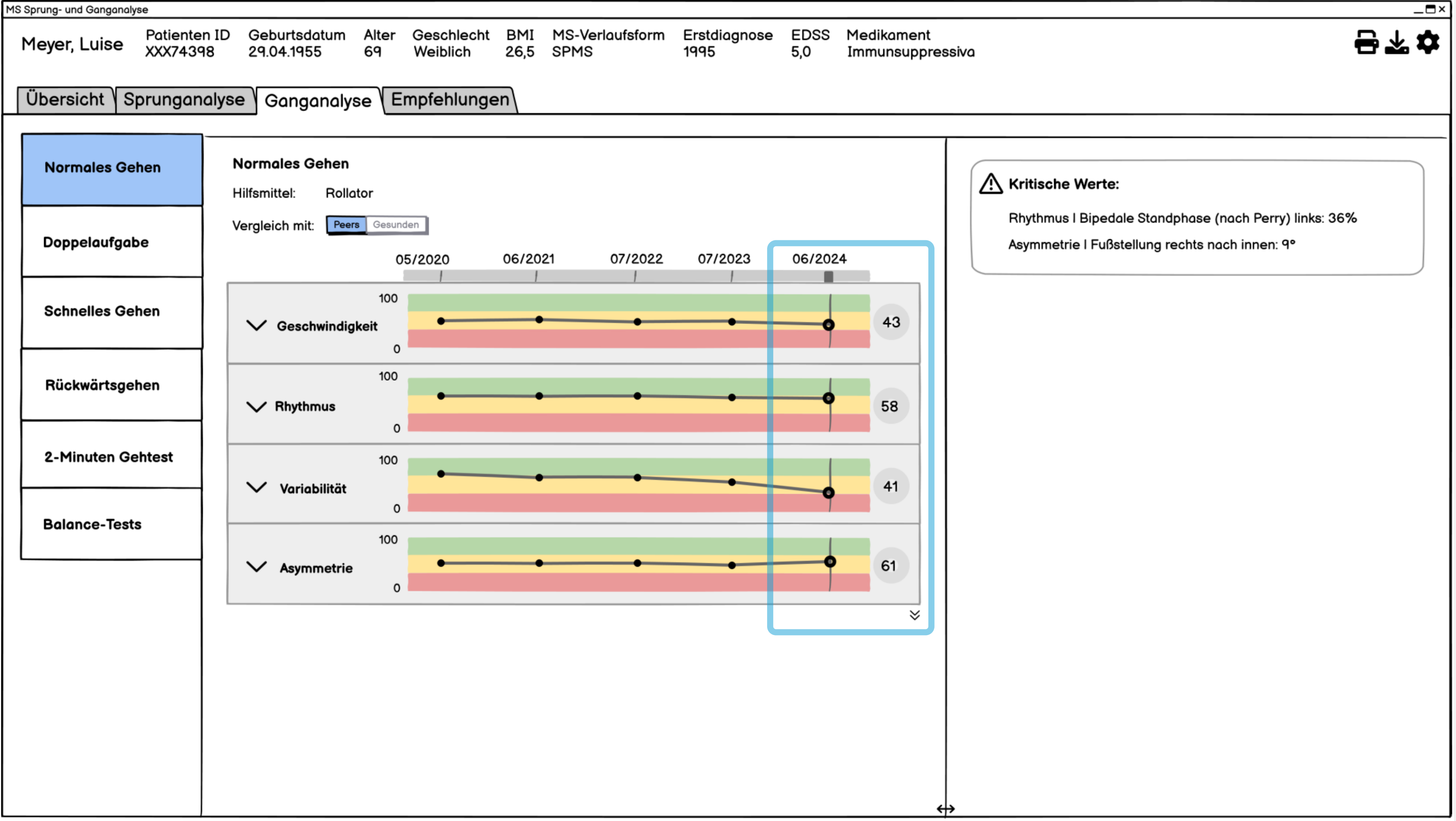

- In this timeline, a **slider** allows selecting the measurement time point from which values are displayed at the end of each trend line.
- By default, the **most recent value** is always selected.

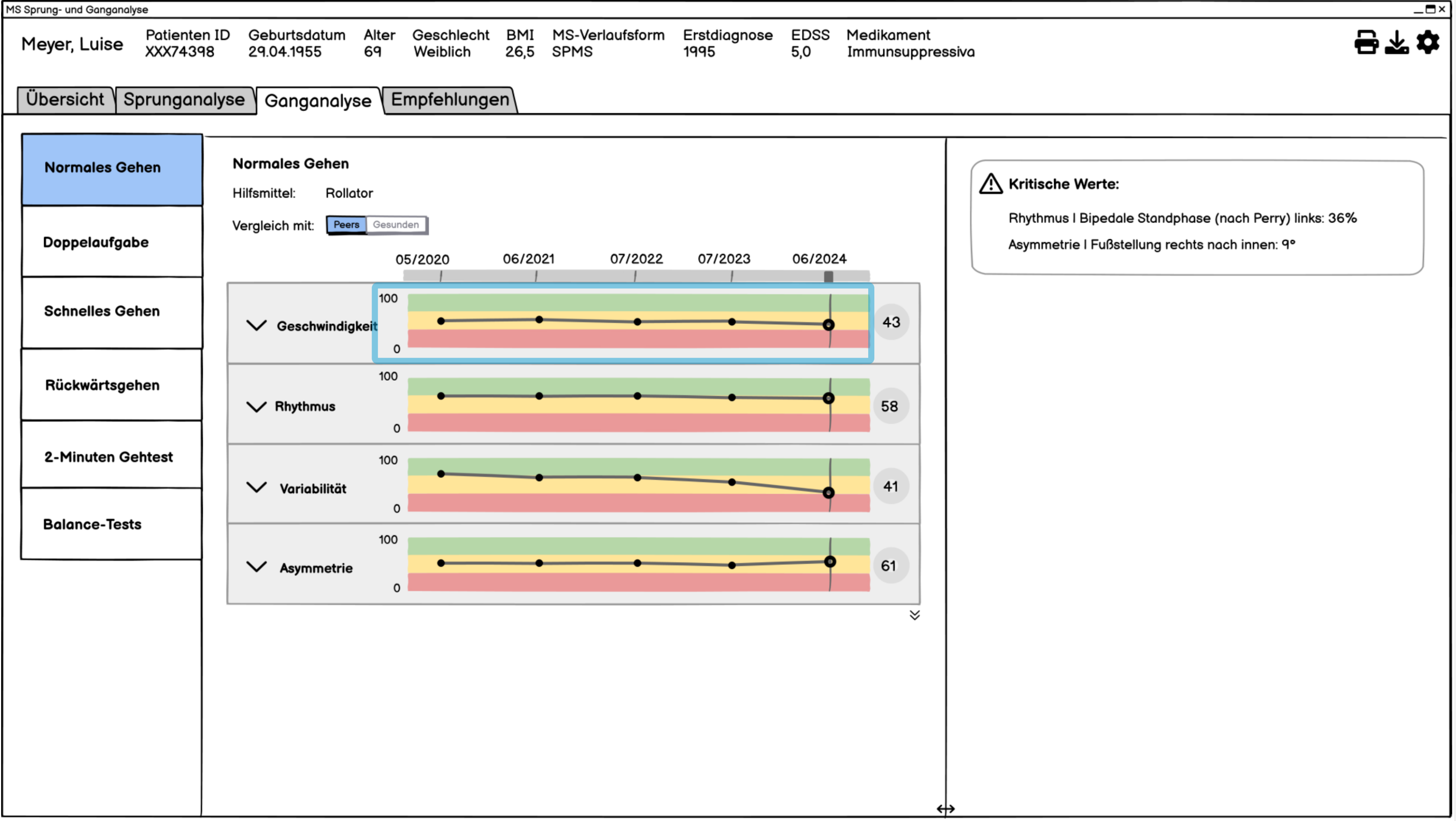

- The **colored areas** in the trend charts indicate how the value compares to peers.
- Green represents above-average to average performance, yellow indicates slightly below average, and red shows significantly below average / critical values.

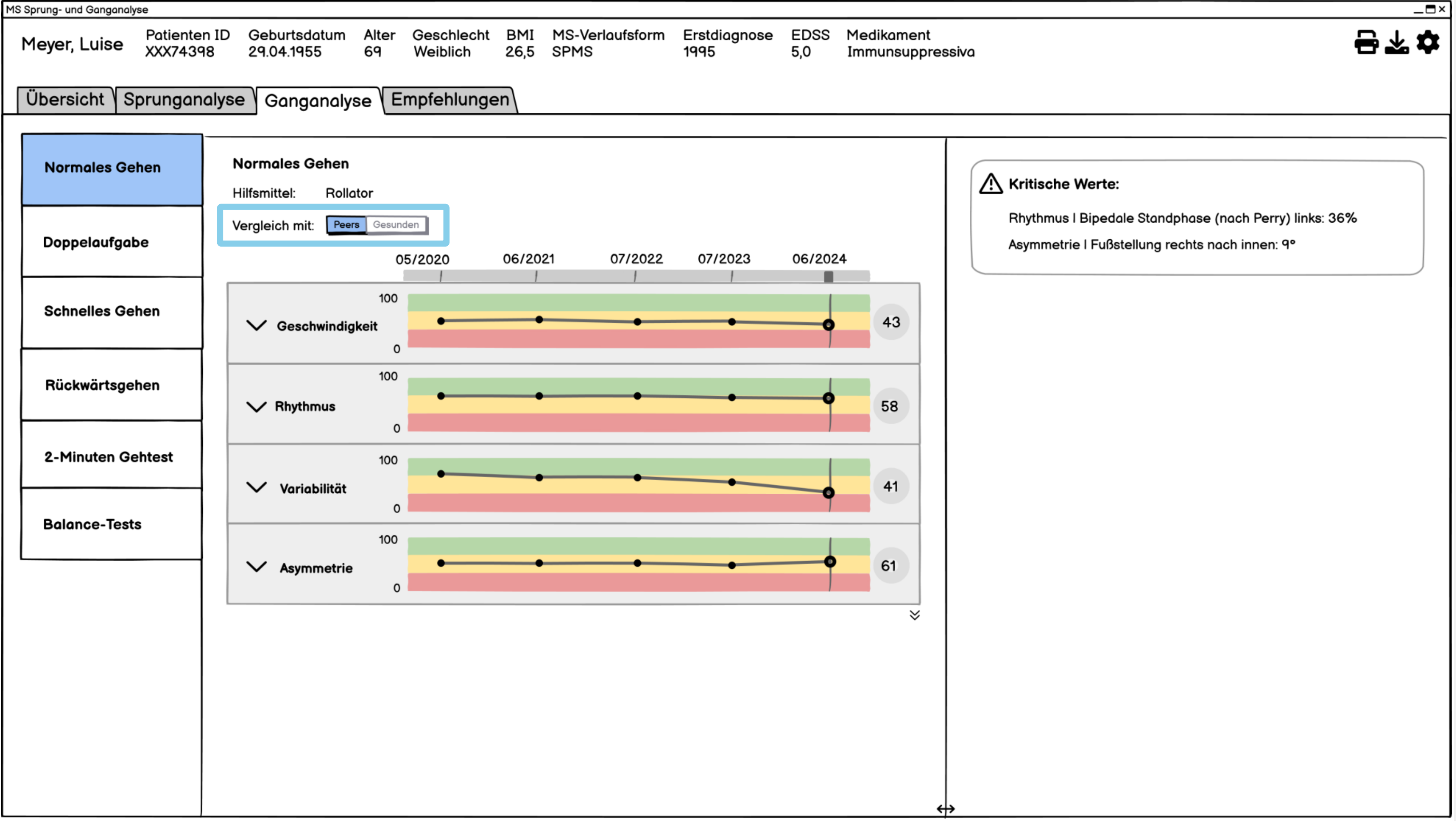

- The **switch** Peers | Gesunden **(Peers | Healthy)** allows changing the comparison group.
- The colored areas in the charts update accordingly.

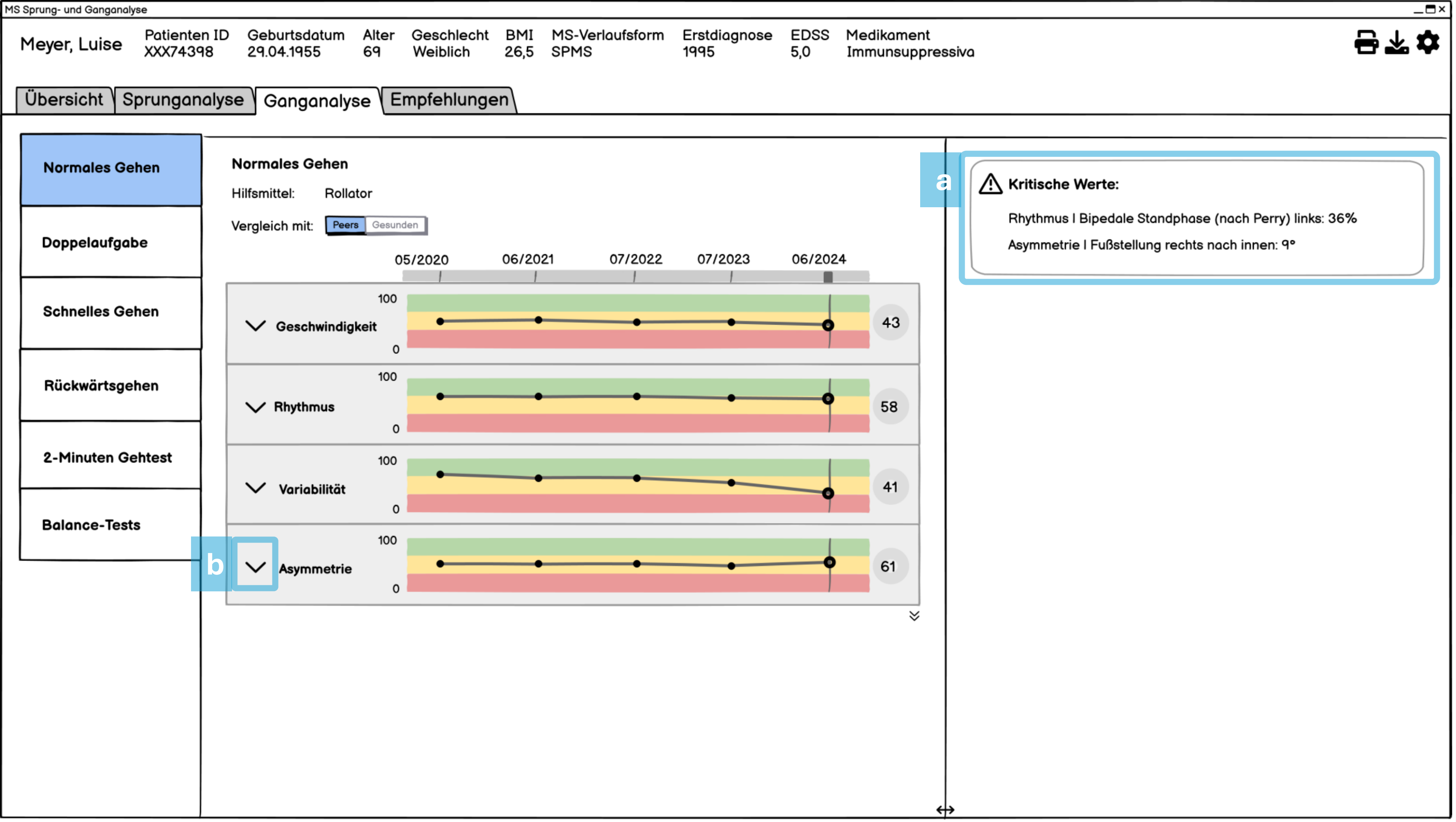

a

- This box displays the **critical values** ⚠ identified in the Normal Walking test.
- The value category is shown first, followed by the detailed value.

b

- To see how the overall score for a category (e.g., **Asymmetry**) is calculated, the section can be **expanded**.

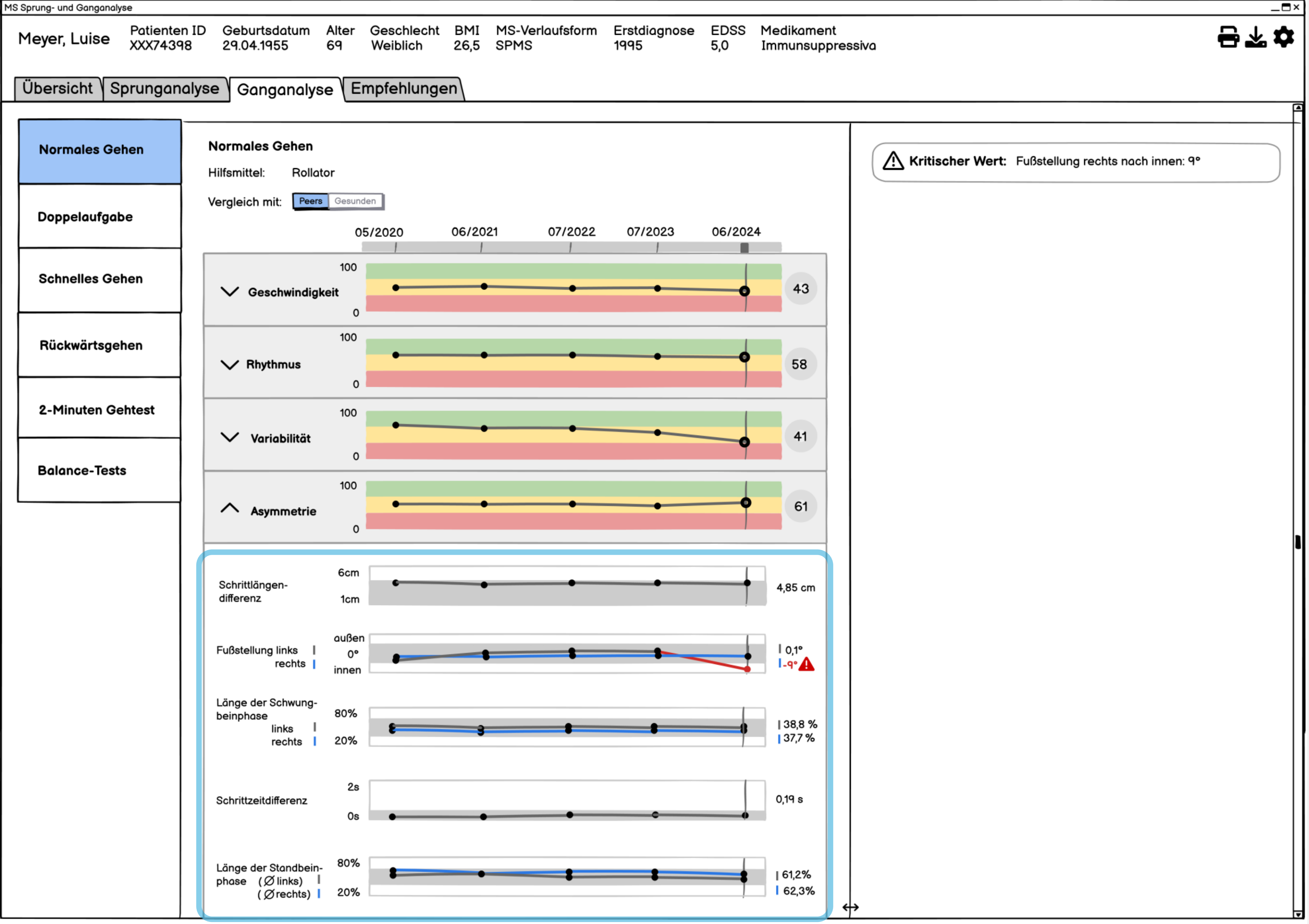

- The corresponding **measured values** are displayed and shown as numbers at the end of each trend line. 43
- For each parameter, the **upper and lower bounds** of the value range are indicated.
- The **gray-shaded area** marks the **normal range** for the selected comparison group.
- In the example of left foot position, the most recent value is **-9°**, which is **outside the normal range** and therefore marked **in red** with a **warning triangle.**
- Some values are measured for **both left** [links] **and right** [rechts] **sides**; in these cases, the trend lines are displayed in **different colors.**

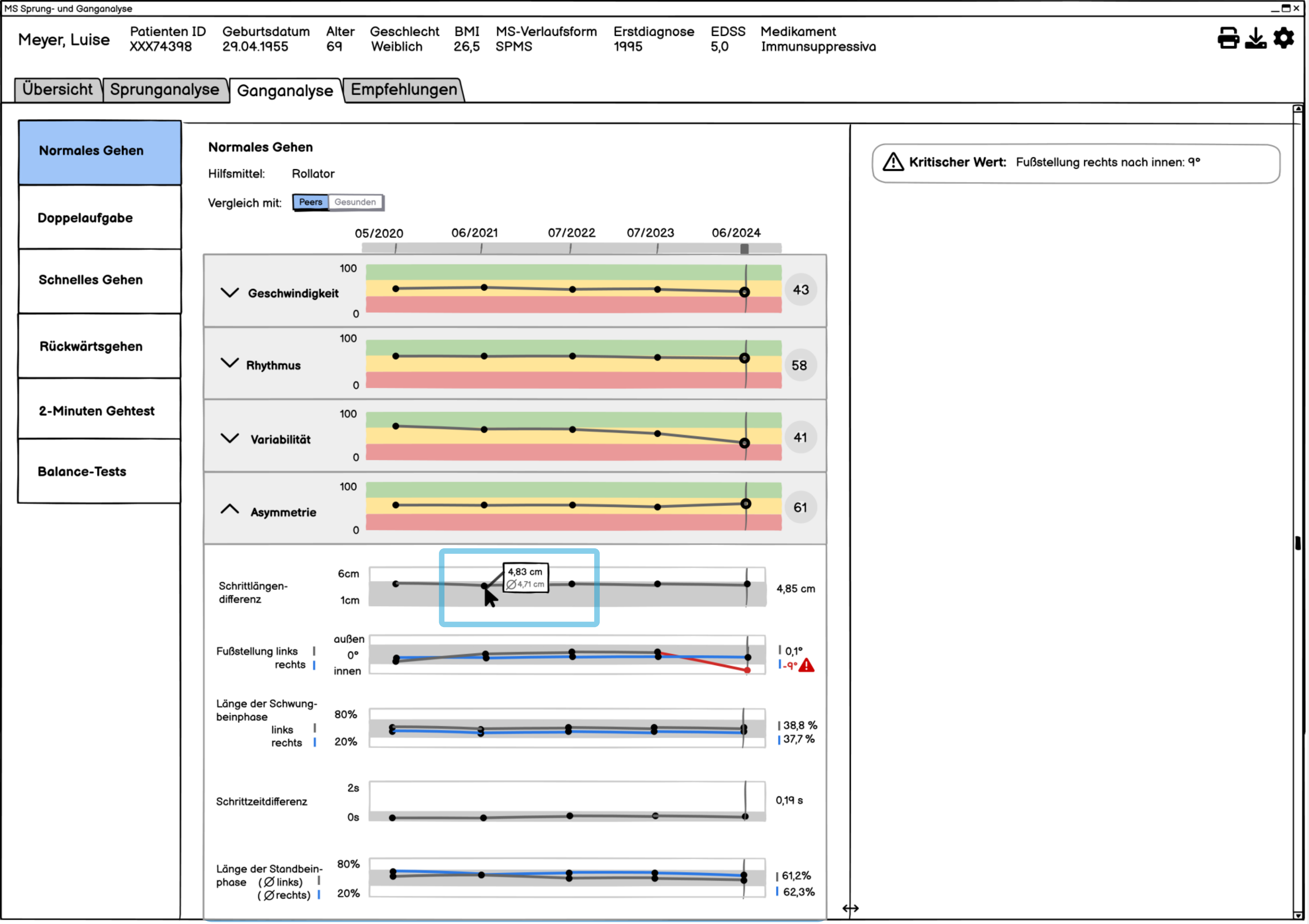

- Clicking on a **point in the trend curve** opens a small **pop-up** box.
- The box displays the **individual measurement value for that point**.
- Below it, the average value Ø of this parameter across all visits is shown.

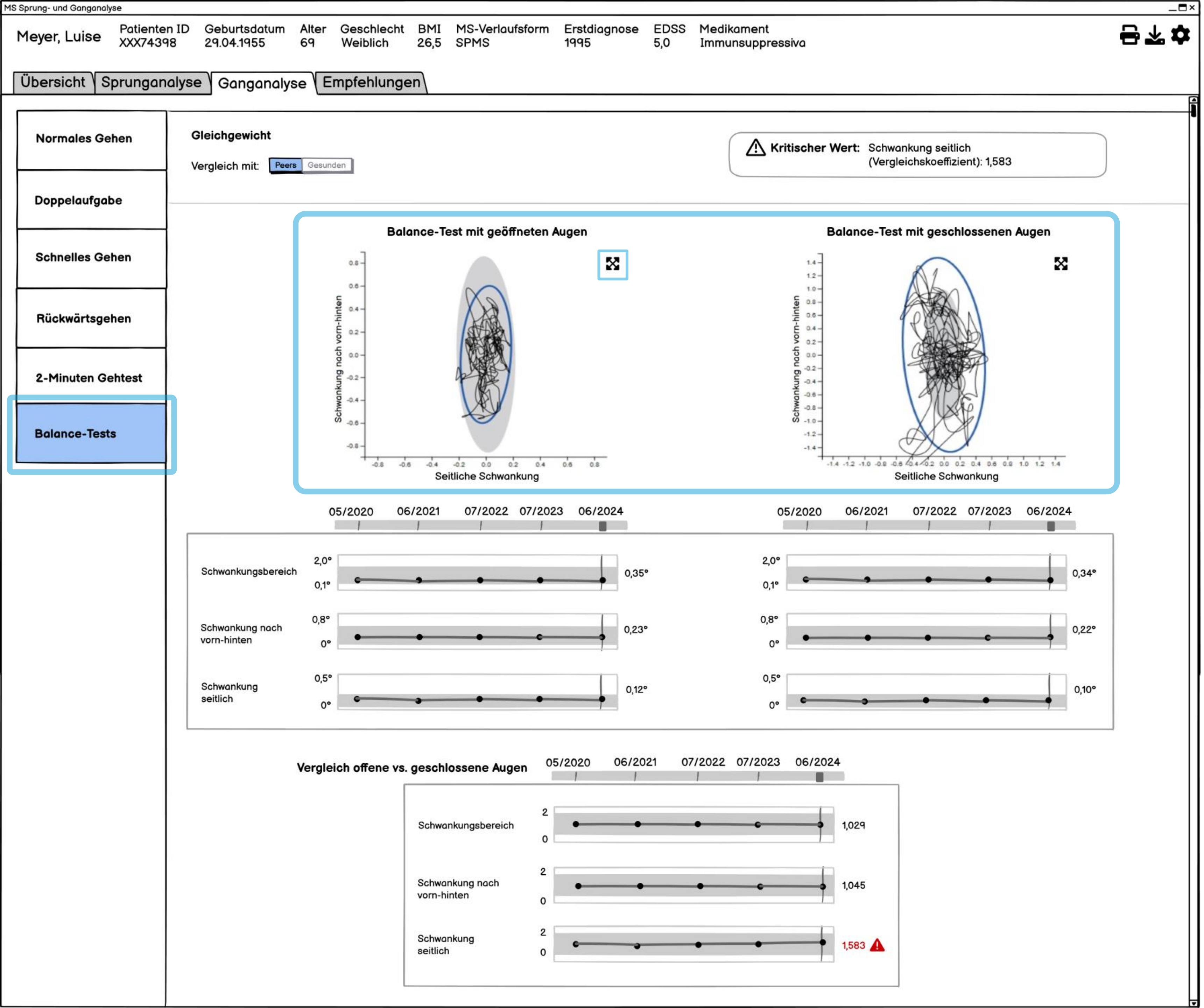

- Clicking on **“Balance Tests”** opens the section containing parameters related to balance.
- At the top, the **most recent sway diagrams** of the patient are shown - left for the test with eyes open, and right for the test with eyes closed.
- By clicking the enlarge icon , each diagram can be opened in a separate window.

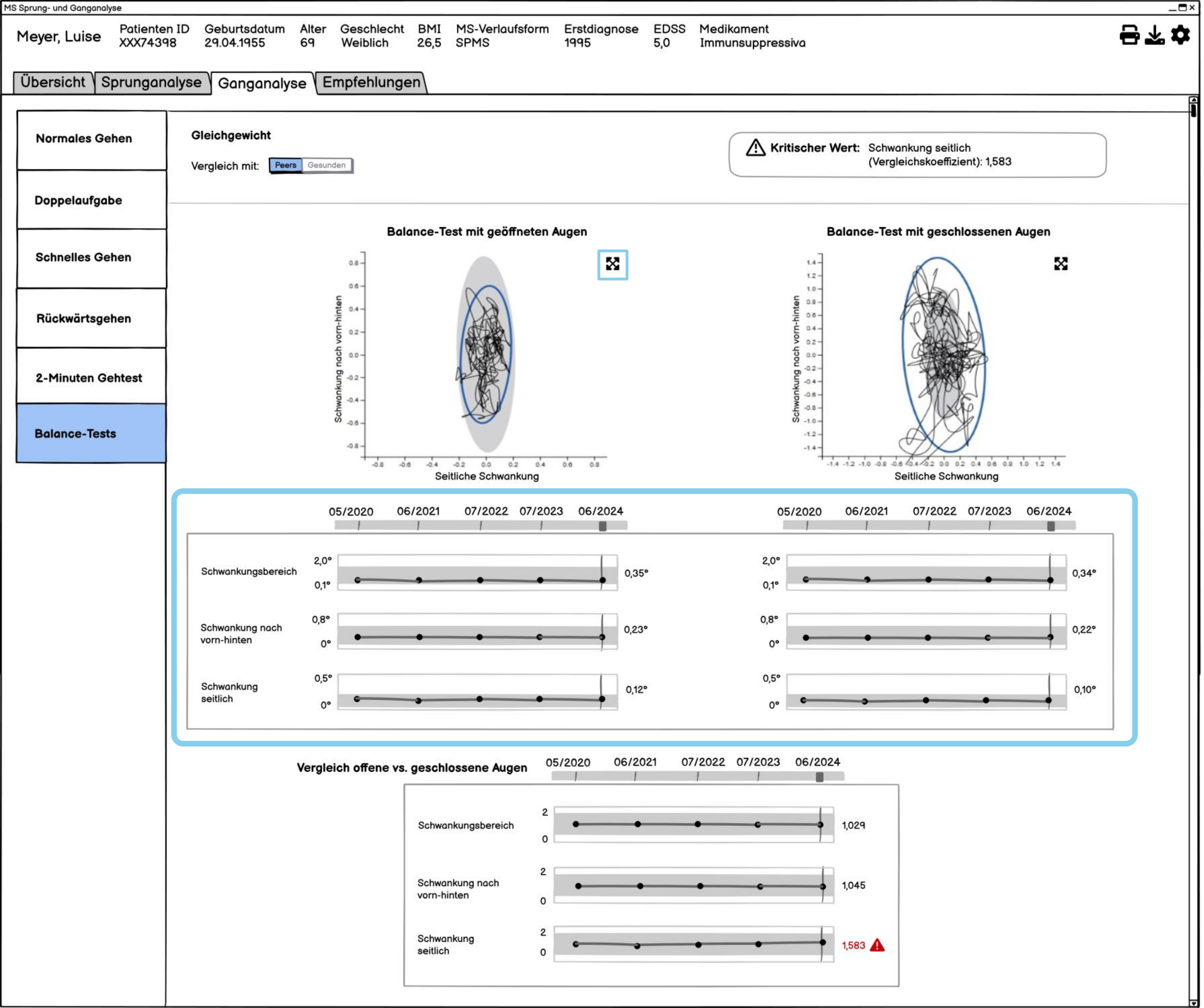

- Below the diagrams, a **timeline** is displayed for each test condition (eyes open vs. eyes closed).
- The **trend of the individual sway degree values** across different examination time points is shown underneath.
- At the end of each trend curve, the value selected via the **timeline slider** is displayed.

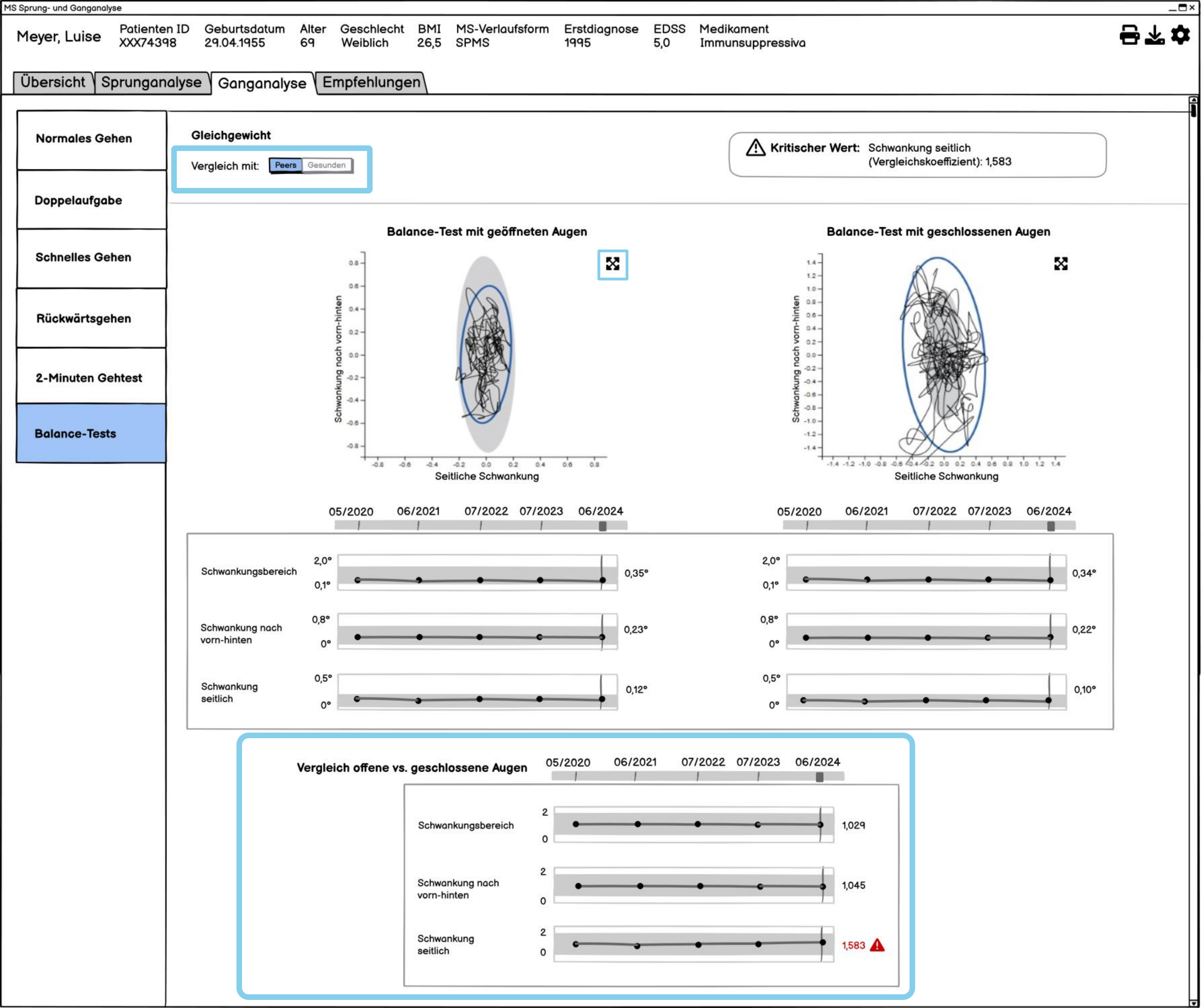

- A **switch toggle** Peers | Gesunden allows selecting whether the patient's values are compared with **peers** or **healthy individuals.**
- The **gray area** behind the trend curve automatically adjusts when switching between comparison groups.

- At the **bottom of the screen**, the **calculated comparison values** for the **balance tests with eyes open and eyes closed** are displayed.
- The **critical parameter value** (1.583) for lateral sway is **highlighted in red** and marked with **a warning symbol**.

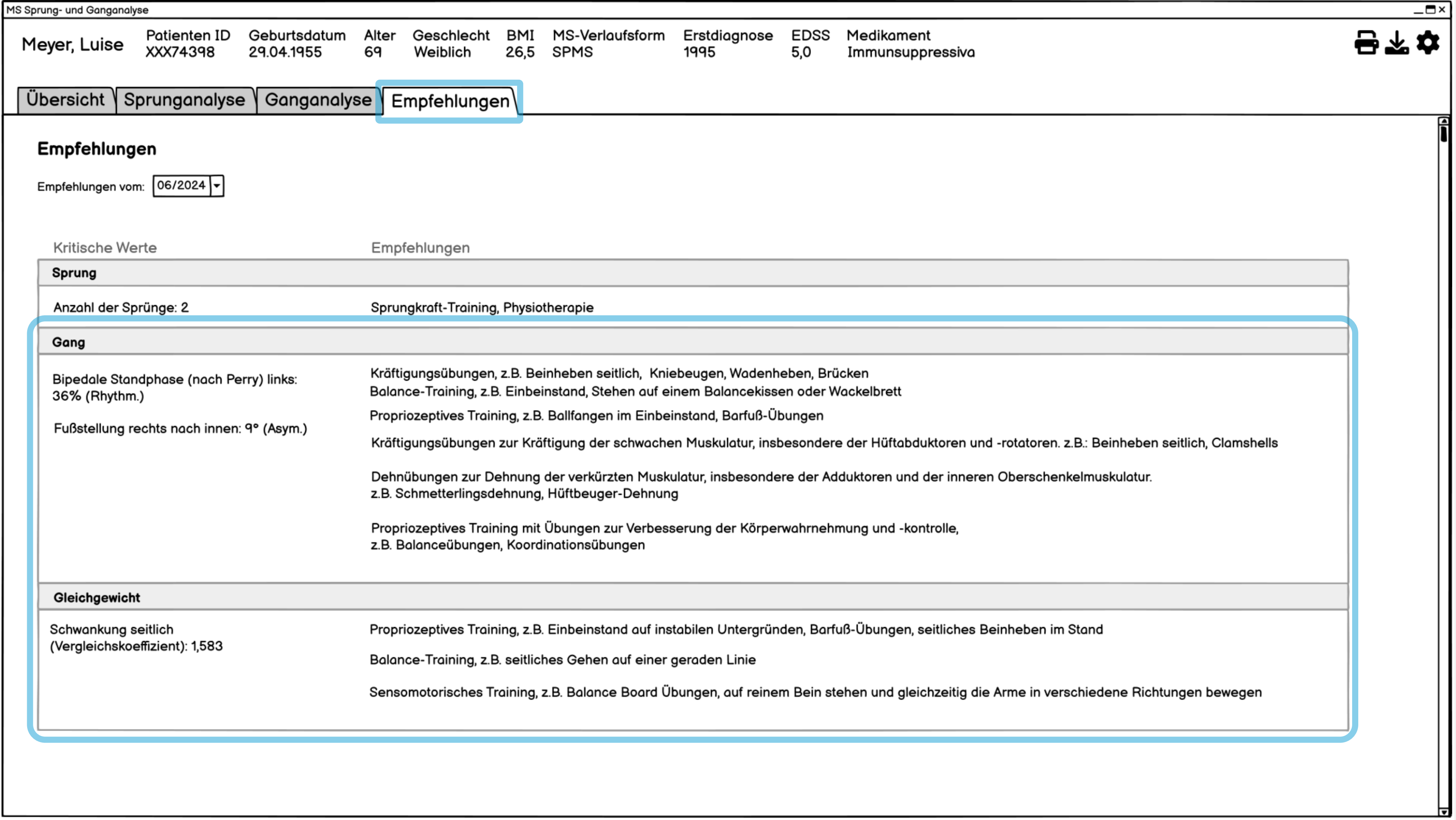

- The **"Recommendations" tab** [Empfehlungen] provides an overview of all critical values from a specific visit and the corresponding recommendations for improving these values.
- By default, **the results from the most recent gait analysis** are displayed.
- Through the **date selection, earlier analysis sessions** can also be **viewed.**
